# One-Pot Synthesis of Polynuclear Indole Derivatives by Friedel–Crafts Alkylation of γ-Hydroxybutyrolactams

**DOI:** 10.3390/molecules28073162

**Published:** 2023-04-02

**Authors:** Vladimir T. Abaev, Nicolai A. Aksenov, Dmitrii A. Aksenov, Elena V. Aleksandrova, Alesia S. Akulova, Igor A. Kurenkov, Alexander V. Leontiev, Alexander V. Aksenov

**Affiliations:** 1 Department of Chemistry, Biology and Biotechnology, North-Ossetian State University Named after K. L. Khetagurov, 46 Vatutin St., Vladikavkaz 362025, Russia; 2 Department of Chemistry, North Caucasus Federal University, 1a Pushkin St., Stavropol 355009, Russia

**Keywords:** polycyclic indoles, intramolecular Friedel–Crafts reaction, cascade transformations

## Abstract

The Friedel–Crafts reaction of novel 3,5-diarylsubstituted 5-hydroxy-1,5-dihydro-2H-pyrrol-2-ones was used for low cost, one-pot preparation of polycyclic indole derivatives structurally similar to Ergot alkaloids.

## 1. Introduction

**Figure 1:**
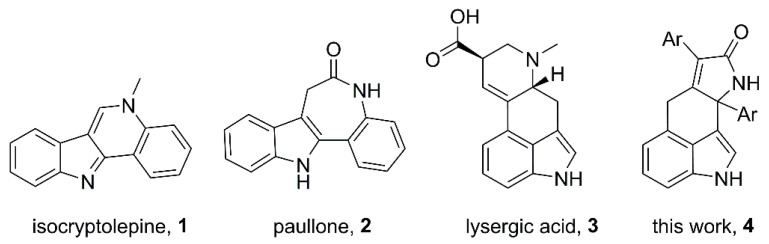
Examples of some polycyclic indole alkaloids.

A vast pool of indole derivatives, both naturally occurring [1] and synthetic [2], have long been a valuable source of potential leads for novel therapeutics, as the majority of indoles are biologically active exhibiting, among others, antitumor [3], anti-Alzheimer’s [4], antiviral [5], antituberculosis [6], antimalarial [7] and antibacterial [8] properties. In this context, a subclass of polynuclear indoles, such as cryptolepine [9], isocryptolepine **1** [10,11] and neocryptolepine [12] alkaloids or paullone **2** derivatives [13], stand somewhat alone, because their preparation usually involves time-consuming, multi-step synthetic procedures (Figure 1). Nevertheless, as such multicyclic skeletons are frequently part of bioactive natural substances, there is significant scientific interest toward them. Thus, Ergot alkaloids, for instance, have been the subject of various studies in recent years [14,15,16,17], including a number of total syntheses of Lysergic acid **3** [18,19,20,21]. Herein we would like to present a convenient route to a principally new type of polycyclic indole moiety **4**, which resembles the skeletons of the Ergot alkaloids and may possess some potent bioactivities as well.

## 2. Results and Discussion

**Scheme 1:**
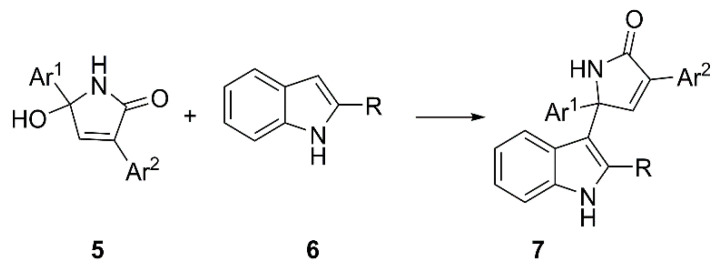
Speculative approach for novel indole butyramide **7**.

Our previous research has shown that (indol-3-yl)acetamides [22] and 2-(indol-3-yl)acetohydroxamic acids [23] demonstrate significant submicromolar in vitro anti-cancer activity against different types of tumor cell lines, while the latter compounds also showed the rare ability to reverse the differentiation of glioma cells, somewhat similar to the mechanism of astrocytes. Moreover, upon the evaluation of a mouse model, some of those indole hydroxamic acids were able to reduce melanoma xenotransplant growth [24]. Encouraged by such results, we turned our attention to 3,5-diarylsubstituted 5-hydroxy-1,5-dihydro-2H-pyrrol-2-one **5**, that was earlier found [25] to undergo a Friedel–Crafts reaction with anilines and phenols. We speculated that the interaction of **5** with indoles **6** would lead to previously unknown 4-(indol-3-yl)butyramide **7**, which are, basically, cyclic analogs of the above indole acetamides/hydroxamic acids (Scheme 1).

It should be noted that substituted γ–hydroxy-γ-butyrolactams [26], which the **5** belongs to, act as a convenient source of corresponding *N*-acyliminium ions upon treatment with acids [27]. The latter undergoes the electrophilic substitution with electron-rich arenes, including indole derivatives [28,29,30]. 

Keeping that in mind, we initially attempted to reproduce the original conditions of our work [25] using microwave heating for the xylene, only to obtain the desired product at a disappointing 11% yield (Table 1, entry 1). Next, we tried different acidic additives with or without solvents as activators (entries 2–7), but such a treatment produced no effect. However, implementing the conditions of *p*-Toluenesulfonic acid (TsOH) in ethanol (EtOH) in a microwave reactor showed the first sign of positive feedback, as the yield increased up to 17% (entry 8). Switching to dimethyl sulfoxide (DMSO) and conventional heating in an oil bath further improved the product outcome (37%, entry 9). After varying the amounts TsOH (entries 10–12), we have finally achieved the decent result using just one equivalent of TsOH (55%, entry 13). 

**Scheme 2:**
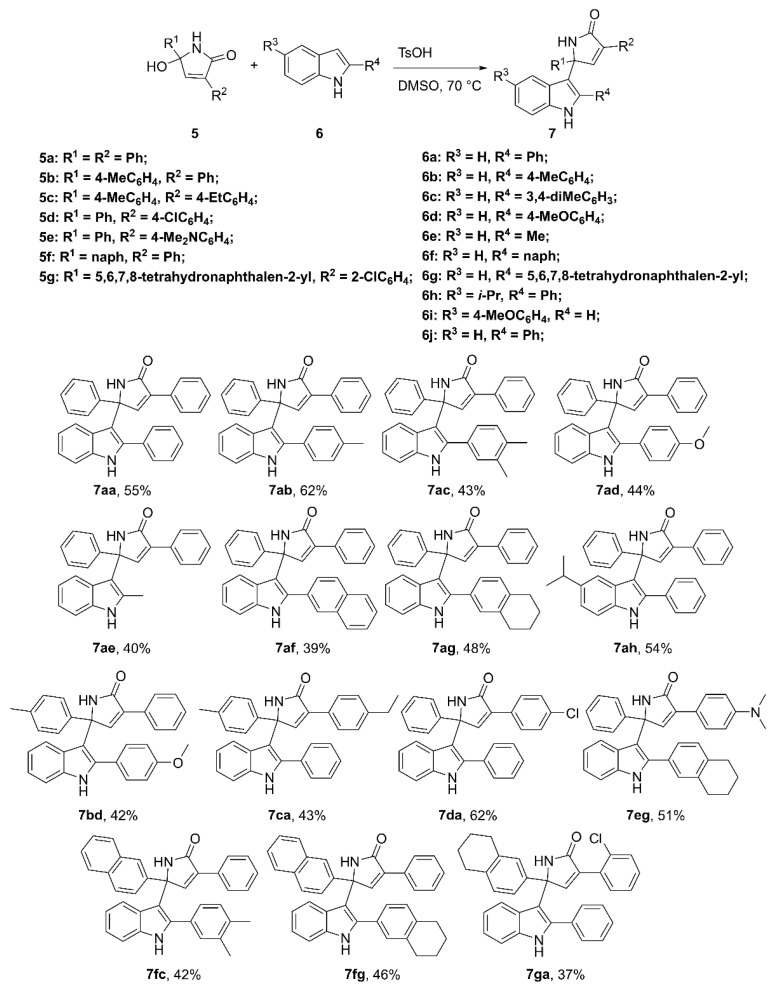
A library of synthesized 5-(1 H-indol-3-yl)-3,5-diaryl-1,5-dihydro-2H-pyrrol-2-ones **7**.

This finding allowed us to proceed with the synthesis of a small, focused library of indole butyramide **7**, while simultaneously evaluating the scope and limitations of this procedure (Scheme 2). As can be seen in the Scheme 2, the reaction appears to be quite tolerant to different substituents in both indoles **6** and 5-hydroxy-1,5-dihydro-2H-pyrrol-2-one **5**, providing reliable 37–55% yields across a range of tested reactants.

**Scheme 3:**
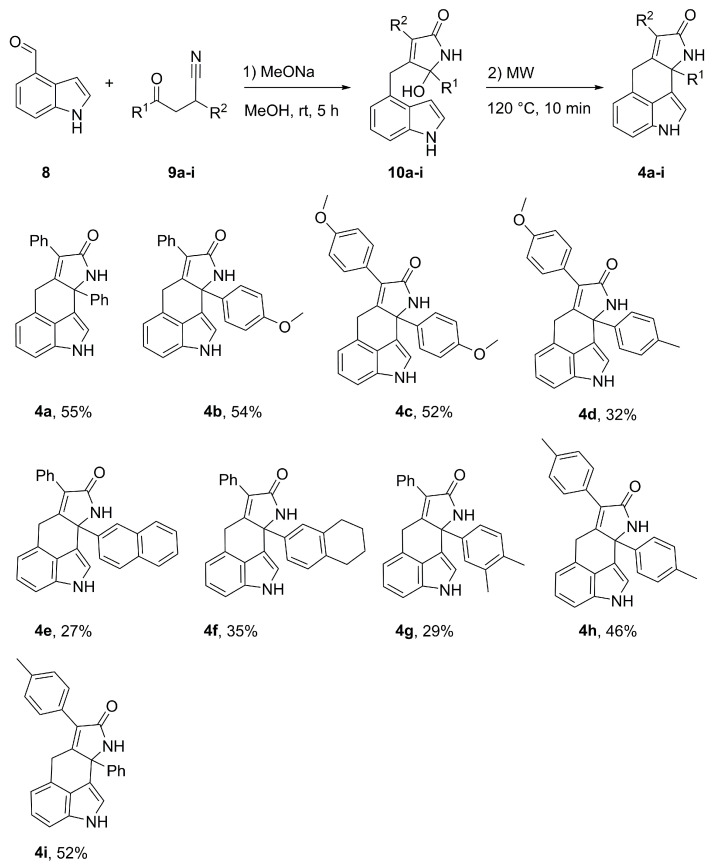
Preparation of 7,9a-diaryl-2,6,9,9a-tetrahydro-8H-indolo[7,6,5-cd]indol-8-one **4a**–**i**.

Once we have learned how to run the Friedel–Crafts reaction between pyrrol-2-one **5** and indole **6**, we were ready to start assembling the corresponding polycyclic indoles **4**. The key step here was to introduce pyrrol-2-one fragment **5** to the C-4 position of indole **6**, in order to then perform the desired intramolecular cyclization. At this point, we speculated that the Knoevenagel condensation of indole-4-carbaldehyde **8** with 2,4-diaryl-4-oxobutyronitrile **9a**–**i** should result in 4-((1H-indol-4-yl)methyl)-5-hydroxy-3,5-diaryl-1,5-dihydro-2H-pyrrol-2-one **10a**–**i**, which upon further heating will transform to 7,9a-diaryl-2,6,9,9a-tetrahydro-8H-indolo[7,6,5-cd]indol-8-one **4a**–**i** (Scheme 3). To our satisfaction, the desired polycyclic indole **4** were indeed obtained this way in a single step, at generally good yields and, importantly, without the need for isolating the intermediate **10**. At the same time, we were able to isolate and characterize indolyl hydroxypyrrolone **10a** (R^1^ = R^2^ = Ph), in agreement with the proposed reaction pathway. Finally, while the reaction turned out to be rather insensitive to the structural features of the starting cyanoketone **9** (Scheme 3), our attempts to use other substituents, R^1^ and R^2^, rather than the aromatic, were unfruitful. Thus, a cyanoketone **9** prepared from benzylideneacetone (R^1^ = Me, R^2^ = Ph) failed to produce both corresponding γ-hydroxybutyrolactam **5** and intermediate **10** under standard conditions.

**Scheme 4:**
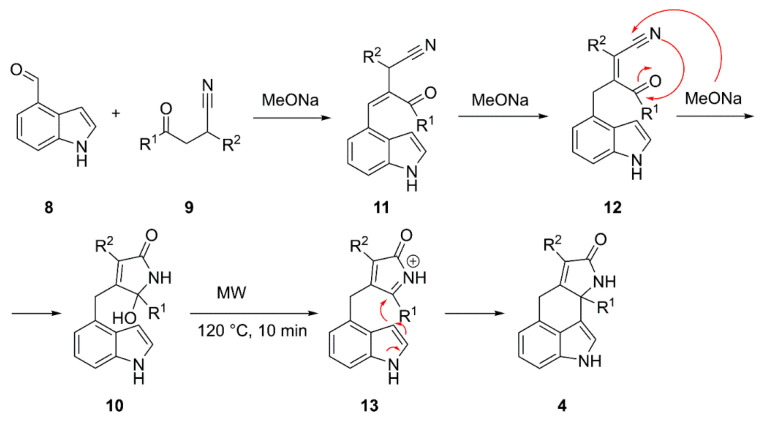
Proposed mechanistic rationale for the formation of polycyclic indoles **4**.

The plausible mechanism of this transformation (Scheme 4) should include the Knoevenagel condensation of indole-4-carbaldehyde **8** with 2,4-diaryl-4-oxobutyronitrile **9** to produce the expected adduct **11**, which further undergoes proton transfer, leading to acrylonitrile **12**. Nucleophilic attack of methoxide anion on the nitrile group, followed by a subsequent nucleophilic attack of the nitrile nitrogen atom on the carbonyl group, results in the formation of intermediate **10**, which gives the reactive iminium cation **13** upon heating. The latter attacks the C-3 position of indole to form the desired polynuclear structure **4**.

**Scheme 5:**
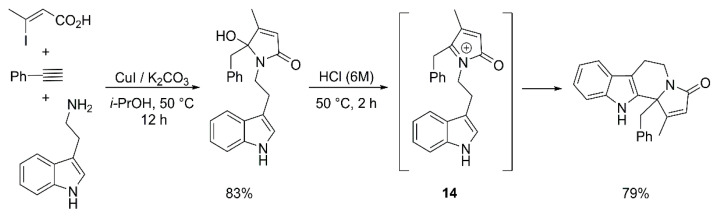
Synthesis of polycyclic indoles by acid-induced Friedel–Crafts alkylation of hydroxybutyrolactams.

One may notice that the given one-pot cascade transformation does not require any acid in order to form reactive *N*-acyliminium cation **13**. Meanwhile, the authors of somewhat conceptually similar work [28] generated such ionic species **14** separately by heating the corresponding 5-hydroxy-1H-pyrrol-2(5H)-one with a 6M HCl solution, at 50 °C for 2 h (Scheme 5). Assumingly, as in our case, the imine-type reactive intermediates **13** can be formed under thermal conditions as well, which makes the step of acid addition unnecessary. Additionally, the intramolecular characteristic of the following then Friedel–Crafts cyclization should greatly favor quenching of the reactive *N*-acyliminium ion, therefore forcing the reaction to reach completion.

## 3. Conclusions

An efficient protocol for the synthesis of potentially bioactive indole butyramide **7** was developed. Intramolecular variation of this reaction leads to the formation of polynuclear indole derivatives **4**, which are structurally similar to the ergot alkaloids and therefore represent a new class of potential pharmacophores. Investigation of biological activity of these compounds is currently under way.

## 4. Experimental Methods

### 4.1. General Information

NMR spectra, ^1^H and ^13^C, were measured in solutions of CDCl_3_ or DMSO-*d*_6_ the Bruker AVANCE-III HD instrument (at 400.40 or 100.61 MHz, respectively). Residual solvent signals were used as internal standards in DMSO*-d_6_* (2.50 ppm for ^1^H, and 40.45 ppm for ^13^C nuclei) or in CDCl_3_ (7.26 ppm for ^1^H, and 77.16 ppm for ^13^C nuclei). HRMS spectra were measured on the Bruker maXis impact (electrospray ionization in MeCN solutions, employing HCO_2_Na–HCO_2_H for calibration). IR spectra were measured on FT-IR spectrometer Shimadzu IRAffinity-1S, equipped with an ATR sampling module. Spectral data are provided in the Supplementary Materials (Figures S1–S50). Reaction progress, purity of isolated compounds, and R*_f_* values were monitored with TLC on Silufol UV-254 plates. Column chromatography was performed using silica gel (32–63 μm, 60 Å pore size). Melting points were measured with the Stuart SMP30 apparatus. All reagents and solvents were purchased from commercial venders, and were used as received.

### 4.2. Preparation of 4-(Indol-3-yl)butyramide ***7*** (General Procedure)

A 5 mL round-bottom flask was charged with indole **6** (1.0 mmol), 3,5-diarylsubstituted 5-hydroxy-1,5-dihydro-2H-pyrrol-2-one **5** (1.0 mmol), TsOH (1.0 mmol) and DMSO (1.0 mL), and the mixture was stirred at 70 °C in an oil bath for 1–2 h, while the reaction progress was monitored by TLC. After complete consumption of the starting material, the mixture was cooled down to room temperature and poured into 20 mL of water, then basified with concentrated aqueous ammonium hydroxide and washed with EtOAc (5 × 20 mL). The combined organic layer was concentrated and purified by column chromatography (EtOAc/Hex, 1:2), followed by recrystallization.

**3,5-Diphenyl-5-(2-phenyl-1H-indol-3-yl)-1,5-dihydro-2H-pyrrol-2-one (7aa):** colorless solid, mp (EtOAc) 261.1–265.3 °C, R*_f_* 0.4 (EtOAc/Hex, 1:2). Yield: 234 mg (0.55 mmol, 55%). ^1^H NMR (400 MHz, DMSO-*d*_6_) δ 11.36 (s, 1H), 9.50 (d, *J* = 1.9 Hz, 1H), 7.58–7.50 (m, 2H), 7.38 (dt, *J* = 7.7, 1.8 Hz, 4H), 7.35–7.29 (m, 5H), 7.28 (dd, *J* = 5.1, 1.8 Hz, 3H), 7.23 (t, *J* = 7.5 Hz, 2H), 7.19–7.14 (m, 1H), 7.05 (t, *J* = 7.4 Hz, 2H), 6.86–6.77 (m, 1H). ^13^C NMR (101 MHz, DMSO-*d*_6_) δ 170.9, 148.7, 142.5, 136.4, 135.7, 133.9, 131.4, 130.5 (2C), 130.1, 128.3 (2C), 128.2, 128.1 (2C), 128.0, 127.7 (2C), 127.1, 126.8 (2C), 126.6, 126.3 (2C), 121.4, 120.7, 118.8, 111.8, 111.1, 65.5. IR, v_max_/cm^−1^: 3261, 3042, 1773, 1686, 1558, 1515, 1417, 1242. HRMS (ES TOF) calculated for (M + Na)^+^ C_30_H_22_N_2_NaO 449.1624, found 449.1611 (3.0 ppm).

**3,5-Diphenyl-5-(2-(p-tolyl)-1H-indol-3-yl)-1,5-dihydro-2H-pyrrol-2-one (7ab):** colorless solid, mp (EtOAc) 262.6–264.7 °C, R*_f_* 0.47 (EtOAc/Hex, 1:2). Yield: 273 mg (0.62 mmol, 62%). ^1^H NMR (400 MHz, DMSO-*d*_6_) δ 11.31 (s, 1H), 9.48 (d, *J* = 2.0 Hz, 1H), 7.54–7.44 (m, 2H), 7.41–7.34 (m, 2H), 7.32–7.28 (m, 3H), 7.26 (dd, *J* = 8.3, 1.9 Hz, 5H), 7.24–7.21 (m, 1H), 7.18 (t, *J* = 7.1 Hz, 1H), 7.12 (d, *J* = 7.8 Hz, 2H), 7.08–6.97 (m, 2H), 6.79 (t, *J* = 7.6 Hz, 1H), 2.30 (s, 3H).^13^C NMR (101 MHz, DMSO-*d*_6_) δ 170.8, 148.7, 142.6, 137.5, 136.4, 135.6, 131.5, 130.9, 130.4 (2C), 129.9, 128.33 (2C), 128.32 (2C), 128.1, 128.0, 127.1, 126.8 (2C), 126.7, 126.2 (2C), 121.3, 120.6, 118.7, 112.0, 111.0, 65.4, 20.9. IR, v_max_/cm^−1^: 3432, 2927, 1684, 1475, 1455, 1364, 1228, 1114. HRMS (ES TOF) calculated for (M + Na)^+^ C_31_H_24_N_2_NaO 463.1781, found 463.1761 (4.4 ppm).

**3-(4-Methoxyphenyl)-5-(2-phenyl-1H-indol-3-yl)-5-(5,6,7,8-tetrahydronaphthalen-2-yl)-1,5-dihydro-2H-pyrrol-2-one (7ac):** colorless solid, mp (EtOAc) 242.1–243.9 °C, R*_f_* 0.31 (EtOAc/Hex, 1:2). Yield: 195 mg (0.43 mmol, 43%). ^1^H NMR (400 MHz, DMSO-*d*_6_) δ 11.28 (s, 1H), 9.50 (s, 1H), 7.54–7.49 (m, 2H), 7.38–7.35 (m, 2H), 7.32 (d, *J* = 6.8 Hz, 1H), 7.30–7.26 (m, 3H), 7.26–7.23 (m, 2H), 7.20–7.17 (m, 2H), 7.14–7.08 (m, 2H), 7.07–7.04 (m, 1H), 7.04–7.00 (m, 1H), 6.97 (d, *J* = 8.6 Hz, 1H), 6.78 (ddd, *J* = 8.1, 7.0, 1.1 Hz, 1H), 2.18 (s, 3H), 2.00 (s, 3H).^13^C NMR (101 MHz, DMSO-*d*_6_) δ 170.89, 148.69, 142.61, 136.51, 136.27, 135.54, 135.46, 132.02, 131.43, 131.13, 129.78, 128.78, 128.3 (2C), 128.1, 128.0 (2C), 127.66, 127.01, 126.7 (2C), 126.2 (2C), 121.16, 120.58, 118.66, 111.82, 110.99, 65.33, 19.20, 19.14. IR, v_max_/cm^−1^: 3384, 2951, 1702, 1684, 1556, 1493, 1459. HRMS (ES TOF) calculated for (M + Na)^+^ C_32_H_26_N_2_NaO 477.1937, found 477.1941 (−0.8 ppm).

**5-(2-(3-Methoxyphenyl)-1H-indol-3-yl)-3,5-diphenyl-1,5-dihydro-2H-pyrrol-2-one (7ad):** colorless solid, mp (EtOAc) 168.8–170.7 °C, R*_f_* 0.38 (EtOAc/Hex, 1:2). Yield: 201 mg (0.44 mmol, 44%). ^1^H NMR (400 MHz, DMSO-*d*_6_) δ 11.37 (s, 1H), 9.52 (d, *J* = 1.9 Hz, 1H), 7.63–7.56 (m, 2H), 7.39–7.35 (m, 2H), 7.35–7.32 (m, 2H), 7.29 (m, *J* = 4.6, 2.7, 2.3 Hz, 3H), 7.24 (m, *J* = 7.9, 6.9, 2.1 Hz, 3H), 7.20–7.15 (m, 1H), 7.08–7.03 (m, 2H), 6.98 (m, *J* = 7.4, 1.3 Hz, 1H), 6.91–6.84 (m, 2H), 6.81 (td, *J* = 7.4, 7.0, 1.1 Hz, 1H), 3.62 (s, 3H). ^13^C NMR (101 MHz, DMSO-*d*_6_) δ 170.9, 158.5, 148.6, 142.5, 136.2, 135.6, 135.1, 131.4, 130.2, 128.8, 128.3 (2C), 128.2, 128.1 (2C), 127.08, 126.7 (2C), 126.6, 126.3 (2C), 122.8, 121.4, 120.6, 118.8, 116.0, 114.0, 111.7, 111.1, 65.4, 54.9. IR, v_max_/cm^−1^: 3197, 2970, 1701, 1687, 1654, 1538, 1455, 1246. HRMS (ES TOF) calculated for (M + Na)^+^ C_31_H_24_N_2_NaO_2_ 479.1730, found 479.1740 (−2.0 ppm).

**5-(2-Methyl-1H-indol-3-yl)-3,5-diphenyl-1,5-dihydro-2H-pyrrol-2-one (7ae):** colorless solid, mp (EtOAc) 183.4–185.6 °C, R*_f_* 0.46 (EtOAc/Hex, 1:1). Yield: 146 mg (0.40 mmol, 40%). ^1^H NMR (400 MHz, DMSO-*d*_6_) δ 11.01 (s, 1H), 9.46 (s, 1H), 8.15 (d, *J* = 2.0 Hz, 1H), 8.09–8.01 (m, 2H), 7.52–7.39 (m, 3H), 7.39–7.33 (m, 4H), 7.32–7.27 (m, 1H), 7.25 (d, *J* = 7.9 Hz, 1H), 6.99 (d, *J* = 7.6 Hz, 1H), 6.97–6.92 (m, 1H), 6.76 (ddd, *J* = 8.2, 7.1, 1.2 Hz, 1H), 2.13 (s, 3H). ^13^C NMR (101 MHz, DMSO-*d*_6_) δ 170.8, 148.0, 142.9, 134.9, 133.2, 131.7, 130.9, 128.5 (2C), 128.4 (2C), 128.3, 127.3, 127.02, 126.95 (2C), 126.6 (2C), 120.1, 119.4, 118.5, 110.6, 109.6, 65.8, 13.7. IR, v_max_/cm^−1^: 3217, 2943, 1702, 1654, 1509, 1455, 1376, 1240. HRMS (ES TOF) calculated for (M + Na)^+^ C_25_H_20_N_2_NaO 387.1468, found 387.1473 (−1.2 ppm).

**5-(2-(Naphthalen-2-yl)-1H-indol-3-yl)-3,5-diphenyl-1,5-dihydro-2H-pyrrol-2-one (7af):** bright yellow solid, mp (EtOAc) 218.0–220.4 °C, R*_f_* 0.33 (EtOAc/Hex, 1:2). Yield: 186 mg (0.39 mmol, 39%). ^1^H NMR (400 MHz, DMSO-*d*_6_) δ 11.47 (s, 1H), 9.53 (d, *J* = 1.9 Hz, 1H), 7.95 (s, 1H), 7.90 (d, *J* = 7.2 Hz, 1H), 7.85–7.78 (m, 2H), 7.57–7.45 (m, 3H), 7.39 (m, *J* = 6.9, 1.4 Hz, 5H), 7.35 (d, *J* = 7.9 Hz, 1H), 7.23–7.13 (m, 3H), 7.13–7.02 (m, 5H), 6.84 (t, *J* = 7.6 Hz, 1H). ^13^C NMR (101 MHz, DMSO-*d*_6_) δ 170.8, 148.7, 142.5, 136.3, 135.8, 132.3, 132.2, 131.3, 131.2, 130.3, 129.7, 128.2 (2C), 128.13, 128.07, 128.0, 127.9 (2C), 127.5, 126.99, 126.97, 126.74, 126.69 (2C), 126.5, 126.33, 126.31 (2C), 121.5, 120.7, 118.9, 112.2, 111.2, 65.5. IR, v_max_/cm^−1^: 3217, 2927, 2851, 1733, 1652, 1507, 1477, 1387, 1248, 1163. HRMS (ES TOF) calculated for (M + Na)^+^ C_34_H_24_N_2_NaO 449.1781, found 449.1777 (0.9 ppm).

**3,5-Diphenyl-5-(2-(5,6,7,8-tetrahydronaphthalen-2-yl)-1H-indol-3-yl)-1,5-dihydro-2H-pyrrol-2-one (7ag):** yellowish solid, mp (EtOAc) 189.9–192.4 °C, R*_f_* 0.51 (EtOAc/Hex, 1:2). Yield: 230 mg (0.48 mmol, 48%). ^1^H NMR (400 MHz, DMSO-*d*_6_) δ 11.27 (s, 1H), 9.52 (d, *J* = 1.9 Hz, 1H), 7.55–7.48 (m, 2H), 7.41–7.35 (m, 2H), 7.31–7.26 (m, 5H), 7.25 (d, *J* = 7.7 Hz, 1H), 7.22–7.17 (m, 2H), 7.15 (dd, *J* = 7.6, 1.8 Hz, 1H), 7.02 (m, *J* = 9.4, 4.9, 2.5 Hz, 2H), 6.94 (d, *J* = 1.8 Hz, 1H), 6.90 (d, *J* = 8.1 Hz, 1H), 6.81–6.72 (m, 1H), 2.71–2.56 (m, 4H), 1.69–1.57 (m, 4H).^13^C NMR (101 MHz, DMSO-*d*_6_) δ 170.9, 148.8, 142.6, 136.8, 136.6, 135.8, 135.5, 131.6, 131.5, 130.7, 129.7, 128.30 (2C), 128.26, 128.0, 127.98 (2C), 127.2, 127.0, 126.7, 126.6 (2C), 126.2 (2C), 121.1, 120.6, 118.6, 111.8, 111.0, 65.3, 28.7, 28.6, 22.7, 22.6. IR, v_max_/cm^−1^: 3141, 2931, 2851, 1773, 1651, 1507, 1461, 1248. HRMS (ES TOF) calculated for (M + Na)^+^ C_34_H_28_N_2_NaO 503.2094, found 503.2084 (2.0 ppm).

**5-(5-Isopropyl-2-phenyl-1H-indol-3-yl)-3,5-diphenyl-1,5-dihydro-2H-pyrrol-2-one (7ah):** yellow solid, mp (EtOAc) 202.2–205.4 °C, R*_f_* 0.34 (EtOAc/Hex, 1:2). Yield: 253 mg (0.54 mmol, 54%). ^1^H NMR (400 MHz, DMSO-*d*_6_) δ 11.20 (s, 1H), 9.56 (d, *J* = 2.0 Hz, 1H), 7.57–7.55 (m, 1H), 7.55 (d, *J* = 2.0 Hz, 1H), 7.41–7.38 (m, 2H), 7.36–7.33 (m, 3H), 7.32 (d, *J* = 1.8 Hz, 1H), 7.30–7.28 (m, 3H), 7.27 (d, *J* = 1.8 Hz, 2H), 7.23–7.20 (m, 2H), 7.20–7.15 (m, 2H), 6.93 (dd, *J* = 8.3, 1.6 Hz, 1H), 6.84 (s, 1H), 2.71 (p, *J* = 7.0 Hz, 1H), 1.05 (dd, *J* = 13.6, 6.9 Hz, 6H). ^13^C NMR (101 MHz, DMSO-*d*_6_) δ 170.86, 148.77, 142.52, 138.56, 136.51, 134.3 (2C), 133.98, 131.50, 130.4 (2C), 130.24, 128.1 (2C), 128.10, 128.0 (2C), 127.87, 127.7 (2C), 126.94, 126.8 (2C), 126.4 (2C), 120.52, 117.70, 111.44, 110.65, 65.43, 33.55, 24.55, 24.28. IR, v_max_/cm^−1^: 3193, 2938, 1733, 1654, 1538, 1509, 1431, 1244. HRMS (ES TOF) calculated for (M + Na)^+^ C_33_H_28_N_2_NaO 491.2094, found 491.2103 (−2.0 ppm).

**3-(4-Methoxyphenyl)-5-(2-phenyl-1H-indol-3-yl)-5-(*p*-tolyl)-1,5-dihydro-2H-pyrrol-2-one (7bd):** colorless solid, mp (EtOAc/Hex) 231.0–233.2 °C, R*_f_* 0.30 (EtOAc/Hex, 1:2). Yield: 197 mg (0.42 mmol, 42%). ^1^H NMR (400 MHz, DMSO-*d*_6_) δ 11.33 (s, 1H), 9.37 (s, 1H), 7.50 (d, *J* = 8.2 Hz, 2H), 7.38–7.27 (m, 6H), 7.22 (d, *J* = 7.8 Hz, 2H), 7.12–7.00 (m, 5H), 6.86–6.79 (m, 3H), 3.74 (s, 3H), 2.21 (s, 3H). ^13^C NMR (101 MHz, DMSO) δ 171.2, 159.1, 146.6, 139.7, 136.2, 136.1, 135.7, 134.0, 130.6 (2C), 129.4, 128.8 (2C), 128.1 (2C), 128.0, 127.7 (2C), 126.7, 126.2 (2C), 124.0, 121.3, 120.8, 118.8, 113.5 (2C), 112.3, 111.0, 65.1, 55.1, 20.6. IR, v_max_/cm^−1^: 3221, 1678, 1501, 1240, 1037. HRMS (ES TOF) calculated for (M + Na)^+^ C_32_H_26_N_2_ NaO_2_ 493.1886, found 493.1896 (−2.0 ppm).

**3-(4-Ethylphenyl)-5-(2-phenyl-1H-indol-3-yl)-5-(p-tolyl)-1,5-dihydro-2H-pyrrol-2-one (7ca):** colorless solid, mp (EtOAc) 234.4–236.6 °C, R*_f_* 0.32 (EtOAc/Hex, 1:2). Yield: 197 mg (0.43 mmol, 43%). ^1^H NMR (400 MHz, DMSO-*d*_6_) δ 11.33 (s, 1H), 9.38 (s, 1H), 7.44 (d, *J* = 8.3 Hz, 2H), 7.39–7.33 (m, 4H), 7.33–7.27 (m, 2H), 7.22 (d, *J* = 8.1 Hz, 2H), 7.18 (s, *J* = 1.8 Hz, 1H), 7.11 (dd, *J* = 8.2, 3.7 Hz, 3H), 7.03 (dd, *J* = 7.7, 5.7 Hz, 3H), 6.82 (t, *J* = 7.6 Hz, 1H), 2.57 (q, *J* = 7.6 Hz, 2H), 2.21 (s, 3H), 1.15 (t, *J* = 7.6 Hz, 3H).^13^C NMR (101 MHz, DMSO-*d*_6_) δ 171.0, 147.8, 143.8, 139.6, 136.2, 136.1, 135.7, 134.0, 130.5 (2C), 129.9, 128.9, 128.8 (2C), 128.0, 127.7 (2C), 127.4 (2C), 126.7 (2C), 126.66, 126.2 (2C), 121.3, 120.8, 118.8, 112.1, 111.0, 65.2, 28.0, 20.6, 15.6. IR, v_max_/cm^−1^: 3193, 2931, 1702 1656, 1509, 1463, 1419, 1395, 1250. HRMS (ES TOF) calculated for (M + Na)^+^ C_33_H_28_N_2_NaO 491.2094, found 491.2094 (−0.1 ppm).

**3-(4-Chlorophenyl)-5-phenyl-5-(2-phenyl-1H-indol-3-yl)-1,5-dihydro-2H-pyrrol-2-one (7da):** colorless solid, mp (EtOAc) 269.6–270.4 °C, R*_f_* 0.39 (EtOAc/Hex, 1:2). Yield: 285 mg (0.62 mmol, 62%). ^1^H NMR (400 MHz, DMSO-*d*_6_) δ 11.40 (s, 1H), 9.53 (d, *J* = 1.9 Hz, 1H), 7.63–7.56 (m, 2H), 7.37 (m, *J* = 8.3, 2.0 Hz, 3H), 7.34 (m, *J* = 4.1 Hz, 4H), 7.32–7.28 (m, 5H), 7.27–7.23 (m, 2H), 7.13–7.03 (m, 2H), 6.86 (td, *J* = 7.5, 7.0, 1.1 Hz, 1H). ^13^C NMR (101 MHz, DMSO-*d*_6_) δ 170.8, 148.3, 141.4 (2C), 136.7 (2C), 135.7, 133.7, 131.6, 131.3, 130.5 (2C), 128.3 (2C), 128.3, 128.2 (2C), 128.1 (2C), 128.0, 127.7 (2C), 126.8 (2C), 126.5, 121.5, 120.4, 119.0, 111.2, 65.1. IR, v_max_/cm^−1^: 3399, 3233, 1685, 1558, 1490, 1457, 1242. HRMS (ES TOF) calculated for (M + Na)^+^ C_30_H_21_ClN_2_NaO 483.1235, found 483.1236 (−0.3 ppm).

**3-(4-(Dimethylamino)phenyl)-5-phenyl-5-(2-(5,6,7,8-tetrahydronaphthalen-2-yl)-1H-indol-3-yl)-1,5-dihydro-2H-pyrrol-2-one (7eg):** yellow solid, mp (EtOAc) 214.2–215.4 °C, R*_f_* 0.28 (EtOAc/Hex, 1:1). Yield: 267 mg (0.51 mmol, 51%). ^1^H NMR (400 MHz, DMSO-*d*_6_) δ 11.27 (s, 1H), 9.23 (d, *J* = 1.9 Hz, 1H), 7.53–7.45 (m, 2H), 7.34–7.28 (m, 5H), 7.28–7.21 (m, 2H), 7.09–7.02 (m, 2H), 7.01–6.92 (m, 2H), 6.89–6.79 (m, 2H), 6.66–6.57 (m, 2H), 2.90 (s, 6H), 2.57 (s, 4H), 1.68–1.60 (m, 4H). ^13^C NMR (101 MHz, DMSO-*d*_6_) δ 171.58, 150.01, 144.4 (2C), 139.67, 136.36, 135.94, 135.63, 135.02, 134.05, 130.4 (2C), 129.64, 128.67, 127.65, 127.6 (2C), 127.5 (2C), 126.73, 123.64, 121.27, 120.77, 119.29, 118.78, 112.44, 111.5 (2C), 111.02, 64.92, 28.9 (2C), 28.4 (2C), 22.76, 22.73. IR, v_max_/cm^−1^: 3221, 2927, 2851, 1688, 1560, 1461, 1435, 1353. HRMS (ES TOF) calculated for (M + Na)^+^ C_36_H_33_N_3_NaO 546.2516, found 546.2488 (5.1 ppm).

**5-(2-(3,4-Dimethylphenyl)-1H-indol-3-yl)-5-(naphthalen-2-yl)-3-phenyl-1,5-dihydro-2H-pyrrol-2-one (7fc):** colorless solid, mp (EtOAc) 173.9–176.4 °C, R*_f_* 0.4 (EtOAc/Hex, 1:2). Yield: 212 mg (0.42 mmol, 42%). ^1^H NMR (400 MHz, DMSO-*d*_6_) δ 11.32 (s, 1H), 9.63 (d, J = 1.9 Hz, 1H), 7.95 (d, *J* = 1.9 Hz, 1H), 7.87–7.78 (m, 2H), 7.72 (d, *J* = 8.7 Hz, 1H), 7.69–7.53 (m, 2H), 7.49–7.44 (m, 2H), 7.42–7.34 (m, 2H), 7.31 (d, *J* = 4.3 Hz, 1H), 7.30–7.27 (m, 3H), 7.15 (dd, *J* = 7.7, 1.8 Hz, 1H), 7.03 (dd, *J* = 7.1, 1.5 Hz, 3H), 7.00 (s, 1H), 6.78–6.70 (m, 1H), 2.08 (s, 3H), 1.91 (s, 3H). ^13^C NMR (101 MHz, DMSO-*d*_6_) δ 171.0, 148.5, 140.1, 136.9, 136.2, 135.5, 135.3, 132.7 132.1, 132.0, 131.5, 131.0, 130.2, 128.6, 128.1, 128.0 (3C), 127.8, 127.6, 127.4, 126.7 (2C), 126.7, 126.2, 126.0, 125.5, 123.8, 121.2, 120.4, 118.8, 111.4, 111.1, 65.5, 19.1, 19.0. IR, v_max_/cm^−1^: 3750, 2931, 1704, 1686, 1523, 1461, 1364. HRMS (ES TOF) calculated for (M + Na)^+^ C_36_H_28_N_2_NaO 527.2094, found 527.2095 (−0.3 ppm).

**5-(Naphthalen-2-yl)-3-phenyl-5-(2-(5,6,7,8-tetrahydronaphthalen-2-yl)-1H-indol-3-yl)-1,5-dihydro-2H-pyrrol-2-one (7fg):** colorless solid, mp (EtOAc) 227.4–229.2 °C, R*_f_* 0.47 (EtOAc/Hex, 1:2). Yield: 244 mg (0.46 mmol, 46%). ^1^H NMR (400 MHz, DMSO-*d*_6_) δ 11.31 (s, 1H), 9.65 (d, *J* = 1.9 Hz, 1H), 7.95 (d, *J* = 1.9 Hz, 1H), 7.85–7.79 (m, 2H), 7.71 (d, *J* = 8.7 Hz, 1H), 7.64–7.60 (m, 2H), 7.48–7.44 (m, 3H), 7.40 (dd, *J* = 8.6, 1.9 Hz, 1H), 7.33–7.26 (m, 4H), 7.15 (dd, *J* = 7.7, 1.8 Hz, 1H), 7.04–6.96 (m, 2H), 6.95–6.89 (m, 2H), 6.74 (ddd, *J* = 8.1, 7.0, 1.1 Hz, 1H), 2.59–2.51 (m, 2H), 2.50–2.40 (m, 1H), 2.33–2.22 (m, 1H), 1.59–1.41 (m, 4H).^13^C NMR (101 MHz, DMSO-*d*_6_) δ 170.98, 148.59, 140.02, 136.53, 135.59, 135.52, 132.63, 132.06, 131.55, 131.53, 130.54, 130.20, 128.10, 128.1 (2C), 128.0 (2C), 127.97, 127.74, 127.36, 127.15, 126.7 (2C), 126.69, 126.16, 125.92, 125.55, 123.82, 121.16, 120.36, 118.74, 111.24, 111.08, 59.81, 28.60, 28.46, 22.58, 22.50. IR, v_max_/cm^−1^: 3245, 2982, 1775, 1718, 1654, 1560, 1507, 1455, 1242. HRMS (ES TOF) calculated for (M + Na)^+^ C_38_H_30_N_2_NaO 553.2250, found 553.2250 (0.1 ppm).

**3-(2-Chlorophenyl)-5-(2-phenyl-1H-indol-3-yl)-5-(5,6,7,8-tetrahydronaphthalen-2-yl)-1,5-dihydro-2H-pyrrol-2-one (7ga):** orange solid, mp (EtOAc/Hex) 154.1–156.7 °C, R*_f_* 0.31 (EtOAc/Hex, 1:2). Yield: 190 mg (0.37 mmol, 37%). ^1^H NMR (400 MHz, DMSO-*d*_6_) δ 11.35 (s, 1H), 9.37 (s, 1H), 7.46–7.41 (m, 1H), 7.35–7.25 (m, 11H), 7.11–7.01 (m, 3H), 6.92–6.84 (m, 2H), 2.65–2.52 (m, 4H), 1.69–1.62 (m, 4H). ^13^C NMR (101 MHz, DMSO-*d*_6_) δ 170.1, 152.2, 138.9, 136.5, 136.1, 135.7, 135.5, 133.9, 132.4, 131.2, 130.7, 130.6, 130.4 (2C), 129.7, 129.6, 128.8, 127.9, 127.6 (2C), 127.2, 126.8, 126.7, 123.8, 121.4, 120.8, 118.9, 111.2, 111.1, 66.3, 29.0, 28.5, 22.8, 22.7. IR, v_max_/cm^−1^: 3273, 2919, 1682, 1499, 1055, 930. HRMS (ES TOF) calculated for (M + Na)^+^ C_34_H_27_ClN_2_NaO 537.1704, found 537.1716 (−2.3 ppm).

### 4.3. Preparation of Polycyclic Indole ***4*** (General Procedure)

A 10 mL vessel for the microwave reaction was charged with indole-4-carbaldehyde **8** (1.0 mmol), cyanoketone **9** (1.0 mmol), MeONa (4.0 mmol) and methanol (1.0 mL). The mixture was stirred at room temperature for 5 h, then heated in a microwave reactor at 120 °C for 10 min. The reaction mixture was concentrated in vacuo and the residue was purified by preparative column chromatography (eluent EtOAc/Hex, 2:1), followed by recrystallization from dichloromethane.

**7,9a-diphenyl-9,9a-dihydro-2H-indolo[7,6,5-cd]indol-8(6H)-one (4a):** light gray solid, mp (CH_2_Cl_2_) 182.9–185.1 °C, R*_f_* 0.23 (EtOAc/Hex, 2:1). Yield: 199 mg (0.55 mmol, 55%). ^1^H NMR (400 MHz, DMSO-*d*_6_) δ 11.21 (s, 1H), 9.61 (s, 1H), 7.43 (d, *J* = 4.4 Hz, 4H), 7.39–7.35 (m, 2H), 7.31–7.22 (m, 4H), 7.09–7.00 (m, 3H), 6.81 (d, *J* = 7.0 Hz, 1H), 4.02 (d, *J* = 17.6 Hz, 1H), 3.52 (d, *J* = 17.6 Hz, 1H). ^13^C NMR (101 MHz, DMSO-*d*_6_) δ 170.9, 158.7, 142.6, 133.1, 131.4, 129.4 (2C), 129.1, 128.6 (2C), 128.3 (2C), 127.9, 127.5, 127.4, 126.3, 125.9 (2C), 122.8, 120.4, 116.1, 114.1, 109.6, 64.2, 27.2. IR, _vmax_/cm^−1^: 1678, 1495, 1445, 1332, 1256, 1181, 1161, 1101, 1025. HRMS (ES TOF) calculated for (M + Na)^+^ C_25_H_18_N_2_NaO 385.1311, found 385.1315 (−1.0 ppm).

**9a-(4-methoxyphenyl)-7-phenyl-9,9a-dihydro-2H-indolo[7,6,5-cd]indol-8(6H)-one (4b):** light gray solid, mp (CH_2_Cl_2_) 175.3–177.5 °C, R*_f_* 0.20 (EtOAc/Hex, 2:1). Yield: 212 mg (0.54 mmol, 54%). ^1^H NMR (400 MHz, CDCl_3_) δ ^1^H NMR (400 MHz, CDCl_3_) δ 8.60 (s, 1H), 7.63 (s, 1H), 7.39 (d, *J* = 8.8 Hz, 2H), 7.25 (d, *J* = 8.2 Hz, 1H), 7.20–7.13 (m, 5H), 7.10–7.00 (m, 2H), 6.94 (d, *J* = 8.8 Hz, 2H), 6.86 (d, *J* = 7.1 Hz, 1H), 4.14 (d, *J* = 17.4 Hz, 1H), 3.81 (s, 3H), 3.56 (d, *J* = 17.1 Hz, 1H). ^13^C NMR (101 MHz, CDCl_3_) δ 173.5, 159.6, 159.1, 141.4, 133.3, 130.9 (2C), 128.9, 128.7 (2C), 128.4, 127.9, 126.8, 126.3 (2C), 123.8, 123.5, 119.5, 117.0, 115.0, 114.0 (2C), 109.4, 65.1, 55.4, 27.7. IR, _vmax_/cm^−1^: 1678, 1608, 1513, 1443, 1334, 1296, 1248, 1179, 1027. HRMS (ES TOF) calculated for (M + Na)^+^ C_26_H_20_N_2_NaO_2_ 415.1417, found 415.1422 (−1.2 ppm).

**7,9a-bis(4-methoxyphenyl)-9,9a-dihydro-2H-indolo[7,6,5-cd]indol-8(6H)-one (4c):** light gray solid, mp (CH_2_Cl_2_) 190.2–192.9 °C, R*_f_* 0.17 (EtOAc/Hex, 2:1). Yield: 219 mg (0.52 mmol, 52%). ^1^H NMR (400 MHz, DMSO-*d*_6_) δ 11.16 (s, 1H), 9.50 (s, 1H), 7.38 (d, *J* = 7.7 Hz, 2H), 7.34 (s, 1H), 7.26 (d, *J* = 8.2 Hz, 1H), 7.05 (t, *J* = 7.6 Hz, 1H), 7.00 (d, *J* = 8.8 Hz, 2H), 6.91 (d, *J* = 9.0 Hz, 2H), 6.87–6.76 (m, 3H), 4.00 (d, *J* = 17.5 Hz, 1H), 3.78 (s, 3H), 3.68 (s, 3H), 3.51 (d, *J* = 18.0 Hz, 1H). ^13^C NMR (101 MHz, DMSO-*d*_6_) δ 171.0, 158.9, 158.5, 157.5, 134.5, 133.1, 130.6 (2C), 128.2, 127.5, 127.1 (2C), 126.3, 123.7, 122.7, 120.1, 115.9, 114.4, 113.8 (2C), 113.7 (2C), 109.5, 63.6, 55.2, 55.1, 27.2. IR, _vmax_/cm^−1^: 2056, 1895, 1682, 1602, 1513, 1439, 1340, 1298, 1246, 1175, 1033. HRMS (ES TOF) calculated for (M + Na)^+^ C_27_H_22_N_2_NaO_3_ 445.1523, found 445.1530 (−1.6 ppm).

**7-(4-Methoxyphenyl)-9a-(p-tolyl)-2,6,9,9a-tetrahydro-8H-indolo[7,6,5-cd]indol-8-one (4d):** yellowish solid, mp (CH_2_Cl_2_) 163.5–165.1 °C, R*_f_* 0.22 (EtOAc/Hex, 1:1). Yield: 123 mg (0.32 mmol, 32%). ^1^H NMR (400 MHz, DMSO-*d*_6_) δ 11.15 (d, *J* = 2.3 Hz, 1H), 9.51 (s, 1H), 7.40–7.35 (m, 2H), 7.33 (d, *J* = 2.3 Hz, 1H), 7.26 (d, *J* = 8.1 Hz, 1H), 7.09–7.05 (m, 3H), 7.01–6.97 (m, 2H), 6.92–6.85 (m, 2H), 6.79 (d, *J* = 7.1 Hz, 1H), 4.00 (d, *J* = 17.7 Hz, 1H), 3.78 (s, 3H), 3.51 (d, *J* = 17.5 Hz, 1H), 2.23 (s, 3H). ^13^C NMR (101 MHz, DMSO-*d*_6_) δ 171.1, 158.9, 157.3, 139.8, 136.58, 133.1, 130.6 (2C), 129.0 (2C), 128.3, 127.5, 126.3, 125.8 (2C), 123.7, 122.6, 120.2, 115.9, 114.3, 113.7 (2C), 109.5, 63.8, 55.2, 27.2, 20.6. IR, _vmax_/cm^−1^:1702, 1684, 1674, 1466, 1445, 1327, 1256, 1171, 1161, 1015. HRMS (ES TOF) calculated for (M + Na)^+^ C_27_H_22_N_2_NaO_2_ 429.1573, found 429.1584 (2.6 ppm).

**9a-(Naphthalen-2-yl)-7-phenyl-2,6,9,9a-tetrahydro-8H-indolo[7,6,5-cd]indol-8-one (4e):** pale gray solid, mp (CH_2_Cl_2_) 187.7–190.3 °C, R*_f_* 0.25 (EtOAc/Hex, 1:1). Yield: 111 mg (0.27 mmol, 27%). ^1^H NMR (400 MHz, DMSO-*d*_6_) δ 11.26 (d, *J* = 2.4 Hz, 1H), 9.72 (s, 1H), 7.91–7.82 (m, 2H), 7.75 (d, *J* = 9.7 Hz, 1H), 7.49–7.43 (m, 6H), 7.42–7.37 (m, 3H), 7.31 (d, *J* = 8.1 Hz, 1H), 7.26 (dd, *J* = 8.7, 1.9 Hz, 1H), 7.10–7.05 (m, 1H), 6.81 (d, *J* = 7.1 Hz, 1H), 4.04 (d, *J* = 17.7 Hz, 1H), 3.55 (d, *J* = 17.5 Hz, 1H). ^13^C NMR (101 MHz, DMSO-*d*_6_) δ 170.8, 158.7, 140.0, 133.2, 132.7, 132.2, 131.4, 129.44, 129.41 (2C), 128.4, 128.3 (2C), 128.01, 128.0, 127.44, 127.40, 126.4, 126.33, 126.32, 125.1, 123.8, 122.7, 120.6, 116.1, 113.8, 109.6, 64.3, 27.3. IR, _vmax_ /cm^−1^: 1961, 1777, 1668, 1495, 1434, 1332, 1246, 1181, 1137, 1117. HRMS (ES TOF) calculated for (M + Na)^+^ C_29_H_20_N_2_NaO 435.1468, found 435.1455 (−3.0 ppm).

**7-Phenyl-9a-(5,6,7,8-tetrahydronaphthalen-2-yl)-2,6,9,9a-tetrahydro-8H-indolo[7,6,5-cd]indol-8-one (4f):** yellowish solid, mp (CH_2_Cl_2_) 192.0–194.5 °C, R*_f_* 0.66 (EtOAc). Yield: 146 mg (0.35 mmol, 35%). ^1^H NMR (400 MHz, DMSO-*d*_6_) δ 11.16 (d, *J* = 2.4 Hz, 1H), 9.52 (s, 1H), 7.43 (d, *J* = 4.4 Hz, 4H), 7.40–7.34 (m, 1H), 7.32 (d, *J* = 2.3 Hz, 1H), 7.26 (d, *J* = 8.2 Hz, 1H), 7.09–7.01 (m, 1H), 6.94 (d, *J* = 8.1 Hz, 1H), 6.79 (d, *J* = 7.2 Hz, 1H), 6.75 (d, *J* = 2.2 Hz, 1H), 6.68 (dd, *J* = 8.0, 2.3 Hz, 1H), 4.01 (d, *J* = 19.1 Hz, 1H), 3.57 (d, *J* = 17.5 Hz, 1H), 2.66–2.54 (m, 4H), 1.70–1.62 (m, 4H). ^13^C NMR (101 MHz, DMSO-*d*_6_) δ 170.8, 158.9, 139.6, 136.6, 136.0, 133.1, 131.5, 129.4 (2C), 129.2, 128.9, 128.3 (2C), 127.9, 127.5, 126.3, 126.2, 123.3, 122.7, 120.2, 116.0, 114.4, 109.6, 64.0, 29.1, 28.4, 27.3, 22.7 (2C). IR, _vmax_/cm^−1^: 2027, 1733, 1678, 1483, 1332, 1240, 1181, 1163, 1117. HRMS (ES TOF) calculated for (M + Na)^+^ C_29_H_24_N_2_NaO 439.1781, found 439.1789 (1.8 ppm).

**9a-(3,4-Dimethylphenyl)-7-phenyl-2,6,9,9a-tetrahydro-8H-indolo[7,6,5-cd]indol-8-one (4g):** yellowish solid, mp (CH_2_Cl_2_) 185.3–187.1 °C, R*_f_* 0.57 (EtOAc). Yield: 113 mg (0.29 mmol, 29%). ^1^H NMR (400 MHz, DMSO-*d*_6_) δ 11.17 (s, 1H), 9.53 (s, 1H), 7.38–7.19 (m, 6H), 7.13–7.00 (m, 3H), 6.89 (t, *J* = 8.2 Hz, 2H), 6.83–6.74 (m, 1H), 4.00 (d, *J* = 22.9 Hz, 1H), 3.52 (d, *J* = 17.9 Hz, 1H), 2.33 (s, 3H), 2.23 (s, 3H). ^13^C NMR (101 MHz, DMSO-*d*_6_) δ 170.94, 158.17, 139.71, 137.21, 136.63, 133.09, 129.2 (2C), 129.1 (2C), 128.8 (2C), 128.7, 128.5, 127.5, 126.3, 125.8 (2C), 122.7, 120.2, 115.9, 114.3, 109.5, 63.9, 27.2, 20.9, 20.6. IR, _vmax_/cm^−1^: 2047, 1704, 1686, 1654, 1538, 1455, 1349, 1266, 1147, 1118, 1027. HRMS (ES TOF) calculated for (M + Na)^+^ C_27_H_22_N_2_NaO 413.1624, found 413.1617 (−1.7 ppm).

**7,9a-di-p-tolyl-9,9a-dihydro-2H-indolo[7,6,5-cd]indol-8(6H)-one (4h):** light gray solid, mp (CH_2_Cl_2_) 175.8–177.5 °C, R*_f_* 0.26 (EtOAc/Hex, 2:1). Yield: 179 mg (0.46 mmol, 46%). ^1^H NMR (400 MHz, DMSO-*d*_6_) δ 11.16 (d, *J* = 2.3 Hz, 1H), 9.52 (s, 1H), 7.32 (d, *J* = 7.2 Hz, 3H), 7.27–7.22 (m, 3H), 7.06 (dd, *J* = 15.7, 8.5 Hz, 3H), 6.89 (d, *J* = 8.3 Hz, 2H), 6.79 (d, *J* = 7.1 Hz, 1H), 4.00 (d, *J* = 17.7 Hz, 1H), 3.50 (d, *J* = 17.5 Hz, 1H), 2.33 (s, 3H), 2.23 (s, 3H). ^13^C NMR (101 MHz, DMSO-*d*_6_) δ 170.9, 158.2, 139.7, 137.2, 136.6, 133.1, 129.2 (2C), 129.1 (2C), 128.8 (2C), 128.7, 128.5, 127.5, 126.3, 125.8 (2C), 122.7, 120.2, 115.9, 114.3, 109.5, 63.9, 27.2, 20.9, 20.6.IR, _vmax_/cm^−1^: 1905, 1678, 1509, 1443, 1408, 1331, 1257, 1185, 1092, 1030. HRMS (ES TOF) calculated for (M + Na)^+^ C_27_H_22_N_2_NaO 413.1624, found 413.1615 (2.2 ppm).

**9a-Phenyl-7-(p-tolyl)-9,9a-dihydro-2H-indolo[7,6,5-cd]indol-8(6H)-one (4i):** light gray solid, mp (EtOH) 185–187 °C, R*_f_* 0.26 (EtOAc/Hex, 2:1). Yield: 196 mg (0.52 mmol, 52%). ^1^H NMR (400 MHz, DMSO-*d*_6_) δ 11.21 (d, *J* = 1.9 Hz, 1H), 9.59 (s, 1H), 7.38 (d, *J* = 2.2 Hz, 1H), 7.34 (d, *J* = 8.1 Hz, 2H), 7.30–7.22 (m, 6H), 7.08–7.02 (m, 3H), 6.80 (d, *J* = 7.1 Hz, 1H), 4.02 (d, *J* = 17.7 Hz, 1H), 3.52 (d, *J* = 17.6 Hz, 1H), 2.32 (s, 3H). ^13^C NMR (101 MHz, DMSO-*d*_6_) δ 171.0, 158.0, 142.7, 137.3, 133.1, 129.3 (2C), 129.0, 128.8 (2C), 128.54 (2C), 128.49, 127.5, 127.4, 126.3, 125.9 (2C), 122.7, 120.3, 115.99, 114.1, 109.5, 64.1, 27.2, 20.9. IR, _vmax_/cm^−1^: 3399, 1674, 1515, 1485, 1443, 1336, 1094, 1094, 1024, 997. HRMS (ES TOF) calculated for (M + Na)^+^ C_26_H_20_N_2_NaO 399.1468, found 399.1464 (1.0 ppm).

### 4.4. Preparation of 4-((1H-Indol-4-yl)methyl)-5-hydroxy-3,5-diphenyl-1H-pyrrol-2(5H)-one ***10a***

A 5 mL round-bottom flask was charged with indole-4-carbaldehyde **8** (154 mg, 1.0 mmol), cyanoketone **9a** (235 mg, 1.0 mmol), MeONa (216, 4.0 mmol) and methanol (1.0 mL). The mixture was stirred at room temperature for 5 h, while the reaction progress was monitored by TLC. Upon complete conversion, the mixture was diluted with EtAcO (20 mL), washed with water (2 × 5 mL) and concentrated in vacuo. The residue was purified by preparative column chromatography (eluent EtOAc/Hex, 1:1), followed by recrystallization from ethanol. White solid, mp (EtOH) 147–149 °C, R*_f_* 0.45 (EtOAc/Hex, 2:1). Yield: 196 mg (0.52 mmol, 52%). ^1^H NMR (400 MHz, DMSO-*d*_6_) δ 10.95 (s, 1H), 9.09 (s, 1H), 7.45 (d, *J* = 7.3 Hz, 2H), 7.42–7.37 (m, 2H), 7.25 (t, *J* = 7.4 Hz, 2H), 7.22–7.17 (m, 5H), 7.04 (d, *J* = 7.9 Hz, 1H), 6.72–6.64 (m, 1H), 6.61 (s, 1H), 6.40 (d, *J* = 6.7 Hz, 1H), 6.22 (s, 1H), 3.88 (d, *J* = 16.5 Hz, 1H), 3.75 (d, *J* = 16.4 Hz, 1H). ^13^C NMR (101 MHz, DMSO-*d*_6_) δ 171.3, 156.9, 140.5, 135.3, 131.5, 131.1, 128.7 (2C), 128.5, 128.00 (2C), 127.7 (3C), 127.6, 126.9, 126.0 (2C), 124.5, 120.5, 118.0, 109.2, 99.3, 88.4, 28.7. IR, _vmax_/cm^−1^: 3306, 1968, 1891, 1697, 1493, 1346, 1266, 1107, 1051. HRMS (ES TOF) calculated for (M + Na)^+^ C_25_H_20_N_2_NaO_2_ 403.1417, found 403.1422 (−1.2 ppm).

## Figures and Tables

**Table 1:** Screening of reaction conditions for the reaction of 2-phenylindole **6a** with 5-hydroxy-3,5-diphenyl-1,5-dihydro-2H-pyrrol-2-one **5a**.

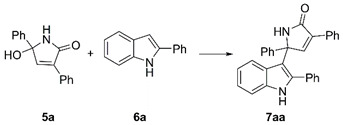				
#	Acid (eq.)	Solvent	Temperature, °C	Yield **7aa**, % ^a^
1	-	Xylene	MW, 100	11
2	H_2_SO_4_ (1)	CH_2_Cl_2_	Reflux	0
3	AcOH (1)	-	70	0
4	H_3_PO_4_ (1)	-	70	0
5	MsOH (1)	-	70	0
6	TsOH (1)	-	70–110	0
7	TsOH (1)	EtOH	Reflux	0
8	TsOH (4)	EtOH	MW, 100	17
9	TsOH (8)	DMSO	50	37
10	TsOH (6)	DMSO	80	38
11	TsOH (4)	DMSO	80	35
12	TsOH (2)	DMSO	70	45
13	TsOH (1)	DMSO	70	55 ^b^

^a^ NMR yields are reported, unless specified otherwise. ^b^ Isolated yield of purified product is provided.

## Data Availability

Supporting information data include NMR and HRMS spectral charts.

## Supplementary Materials

The following supporting information can be downloaded at: https://www.mdpi.com/article/10.3390/molecules28073162/s1, Figures S1–S18: ^1^H and ^13^C NMR spectral charts for polycyclic indoles **4a**–**i**; Figures S19–S48: ^1^H and ^13^C NMR spectral charts for indole butyramides **7aa**–**ah**,**bd**,**ca**,**da**,**eg**,**fc**,**fg**,**ga**; Figures S49 and S50: ^1^H and ^13^C NMR spectral charts for indolyl hydroxypyrrolone **10a**.

## Sample Availability

Samples of the compounds are not available from the authors.
