# Supplementary material for: One-Pot Synthesis of Polynuclear Indole Derivatives by Friedel–Crafts Alkylation of γ-Hydroxybutyrolactams
Source: Molecules. 2023 Apr 2;28(7):3162. doi: 10.3390/molecules28073162 (PMC10095734; doi:10.3390/molecules28073162)
Supplement: Supplementary file 1 [file molecules-28-03162-s001.zip › molecules-2283577-supplementary.pdf]

Electronic Supplementary Material (ESI)

Supporting Information

NMR Spectral Charts…………………………………………………………………………………………………….S2
^{1}H and ^{13}C NMR spectral charts for polycyclic indoles **4a-i**…...…………………………………………………………......S2
^{1}H and ^{13}C NMR spectral charts for indole butyramides **7aa-ah,bd,ca,da,eg,fc,fg,ga.**.………..… …………………………S20
^{1}H and ^{13}C NMR spectral charts for indolyl hydroxypyrrolone **10a**……………………...………..…..……..…………………...S50

^{1}H and ^{13}C NMR spectral charts for 7,9a-diaryl-2,6,9,9a-tetrahydro-8*H*-indolo[7,6,5-*cd*]indol-8-ones **4a-i**

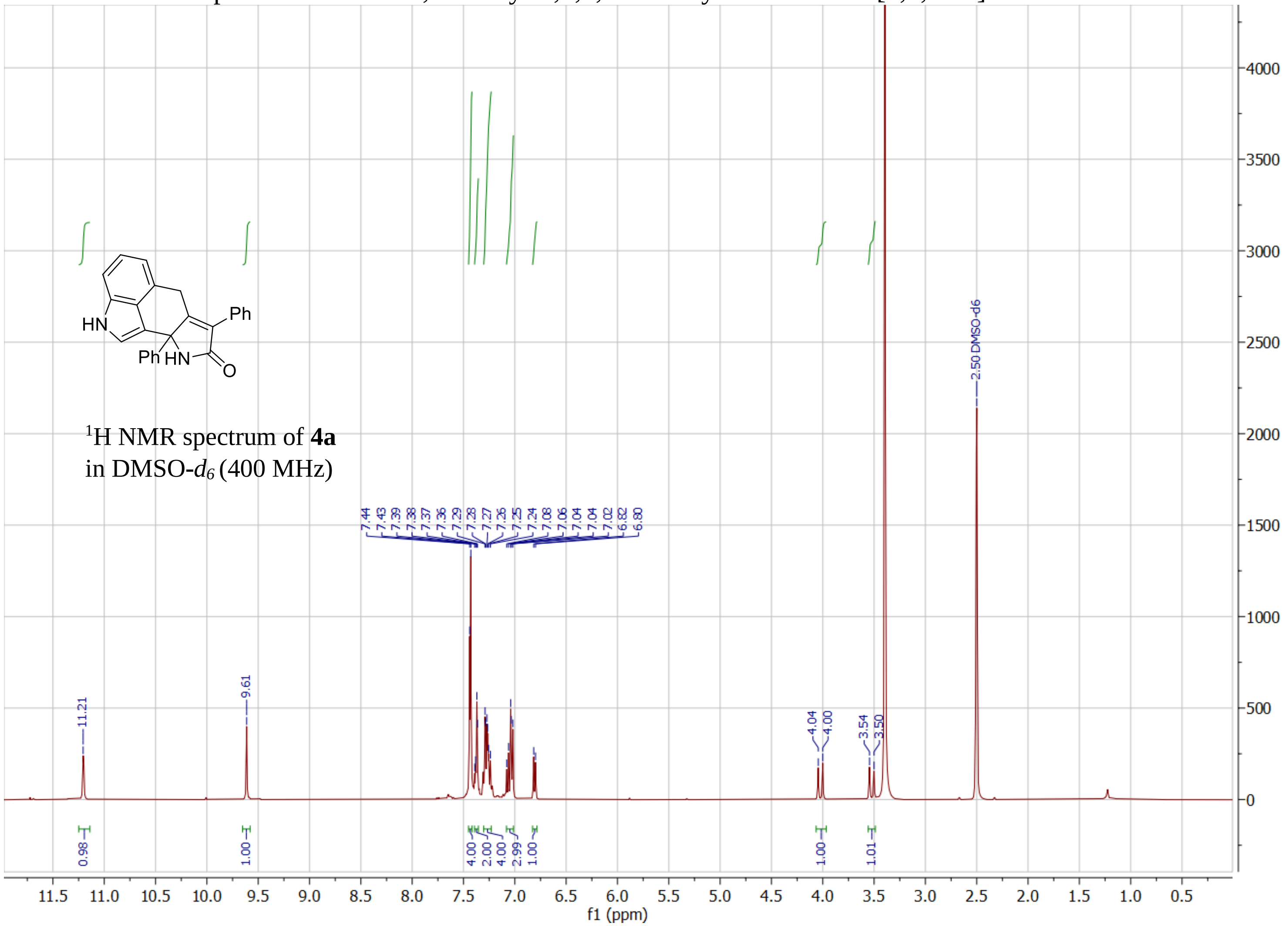

Figure S1. ^{1}H NMR spectrum of **4a** in DMSO-d_6 (400 MHz)

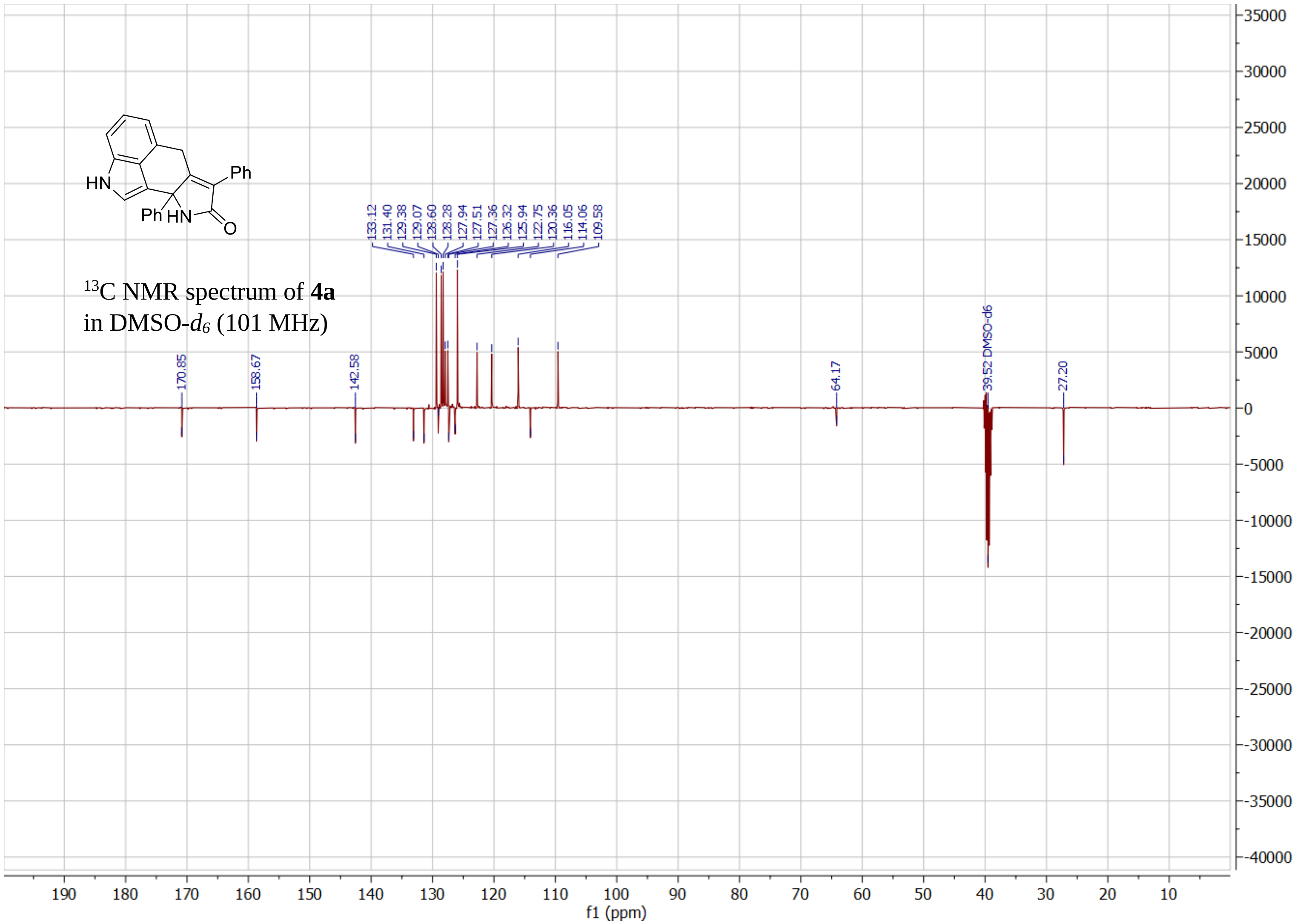

Figure S2. ^{13}C NMR spectrum of **4a** in DMSO-d_6 (101 MHz)

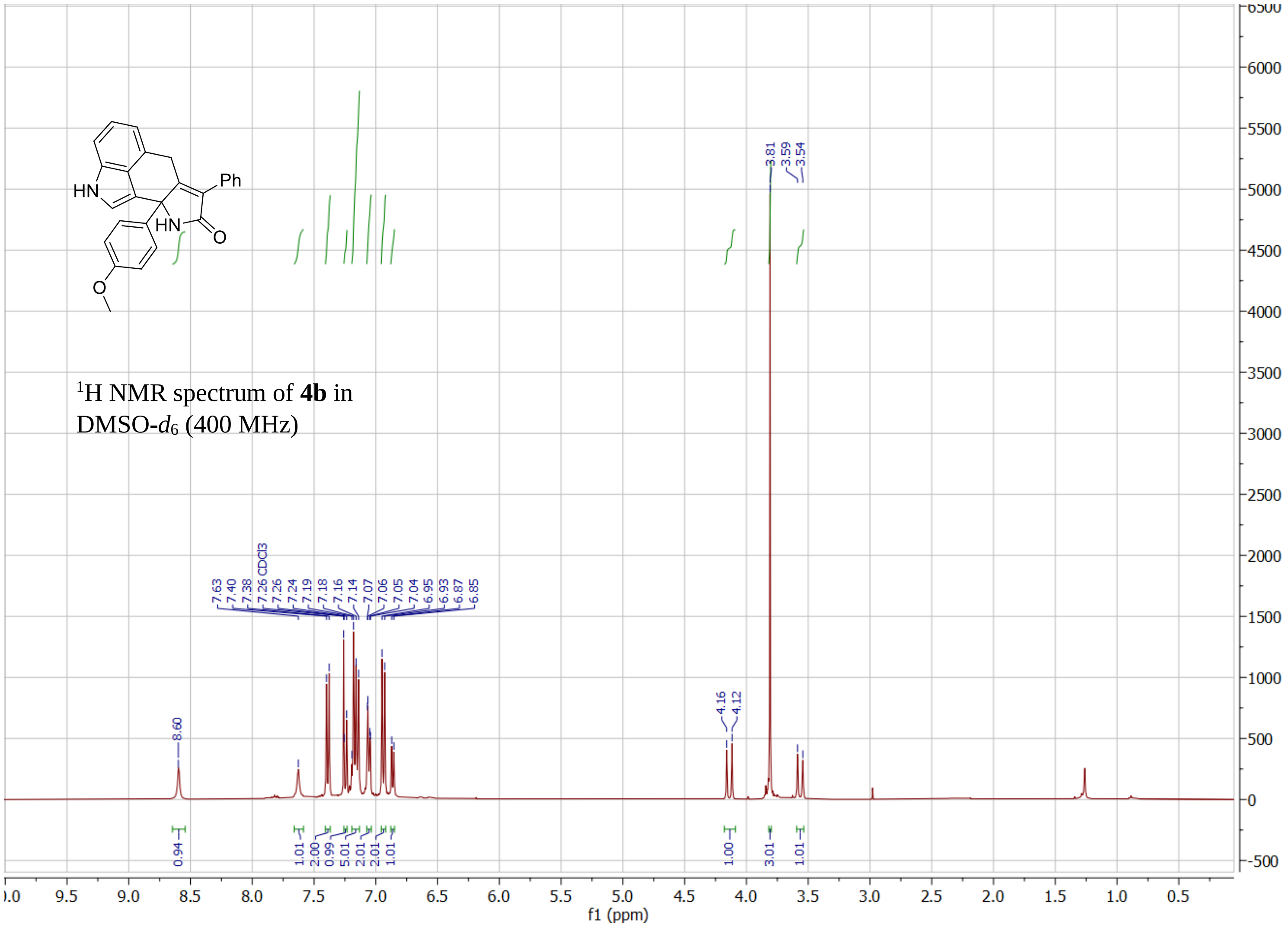

Figure S3. ^{1}H NMR spectrum of **4b** in DMSO-d_6 (400 MHz)

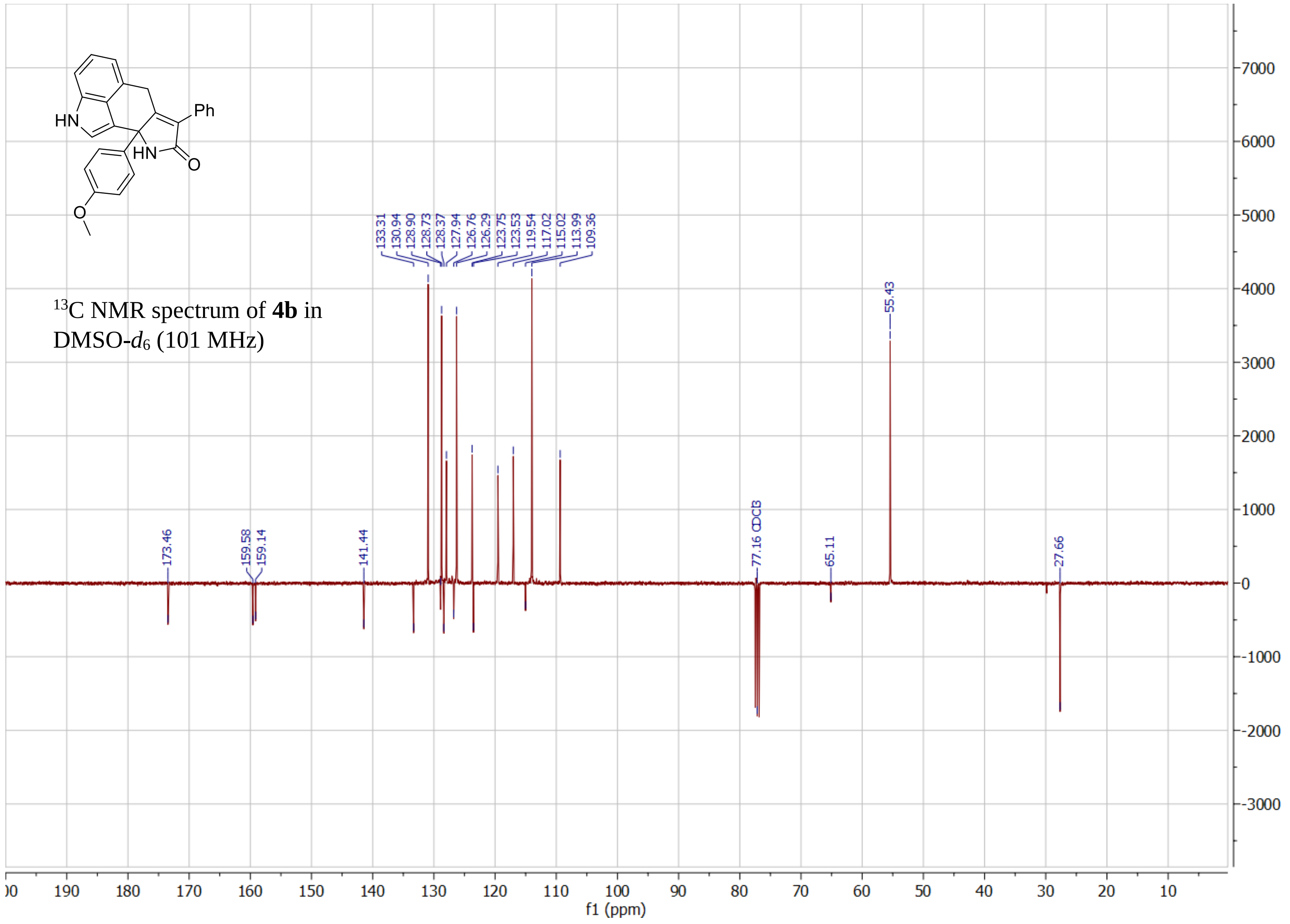

Figure S4. ^{13}C NMR spectrum of **4b** in DMSO-d_6 (101 MHz)

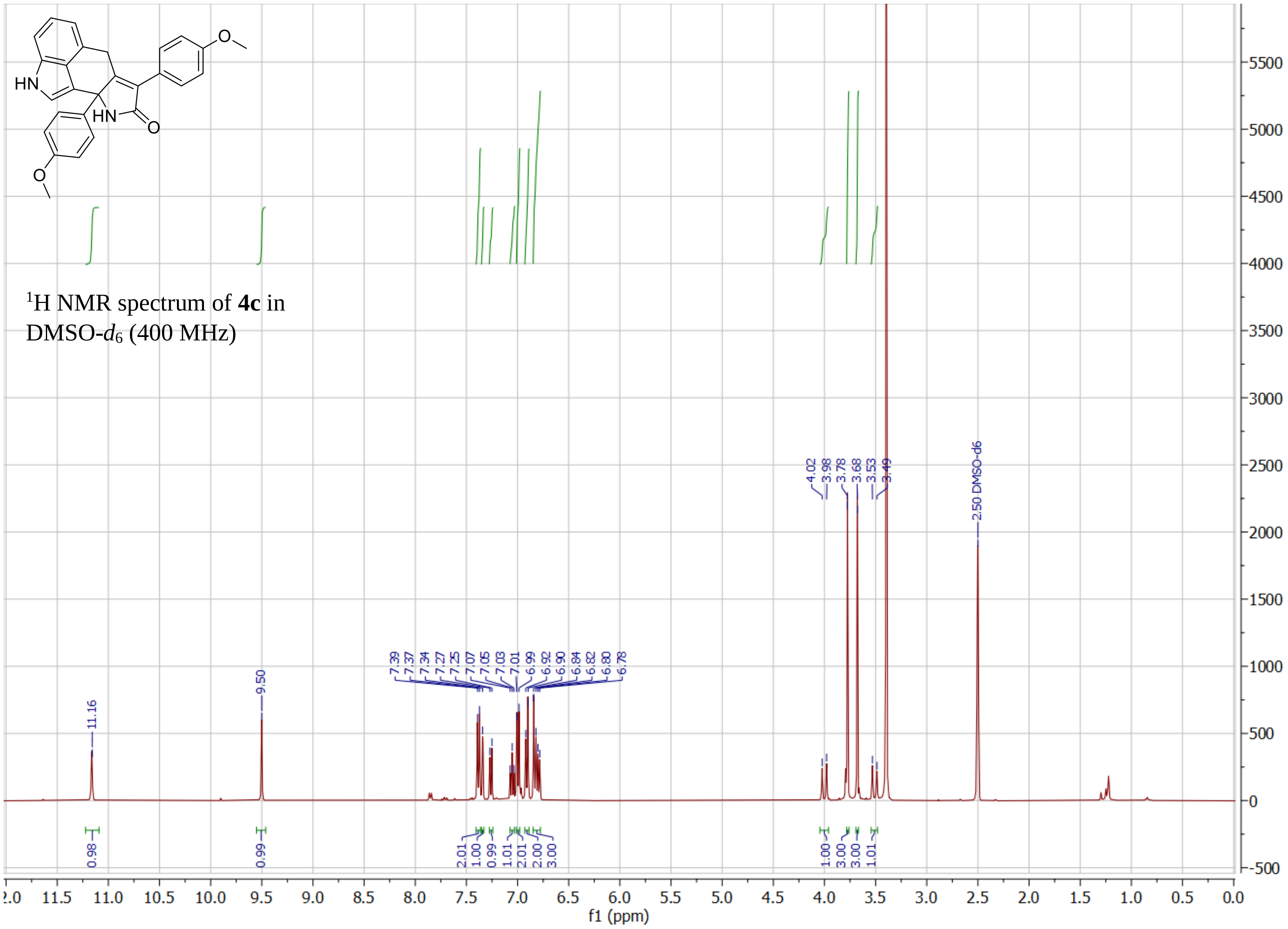

Figure S5. ^{1}H NMR spectrum of **4c** in DMSO-d_6 (400 MHz)

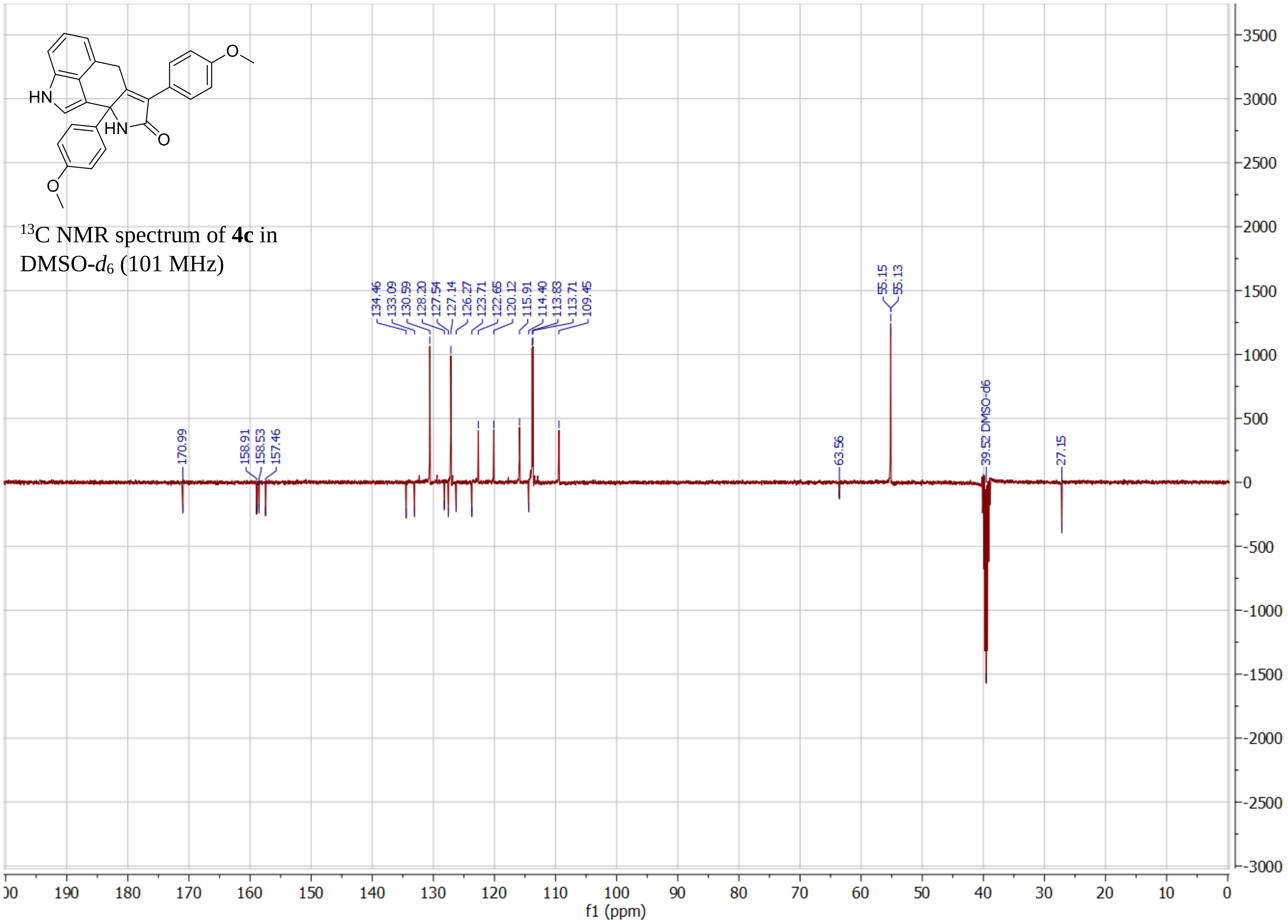

Figure S6. ^{13}C NMR spectrum of **4c** in DMSO-d_6 (101 MHz)

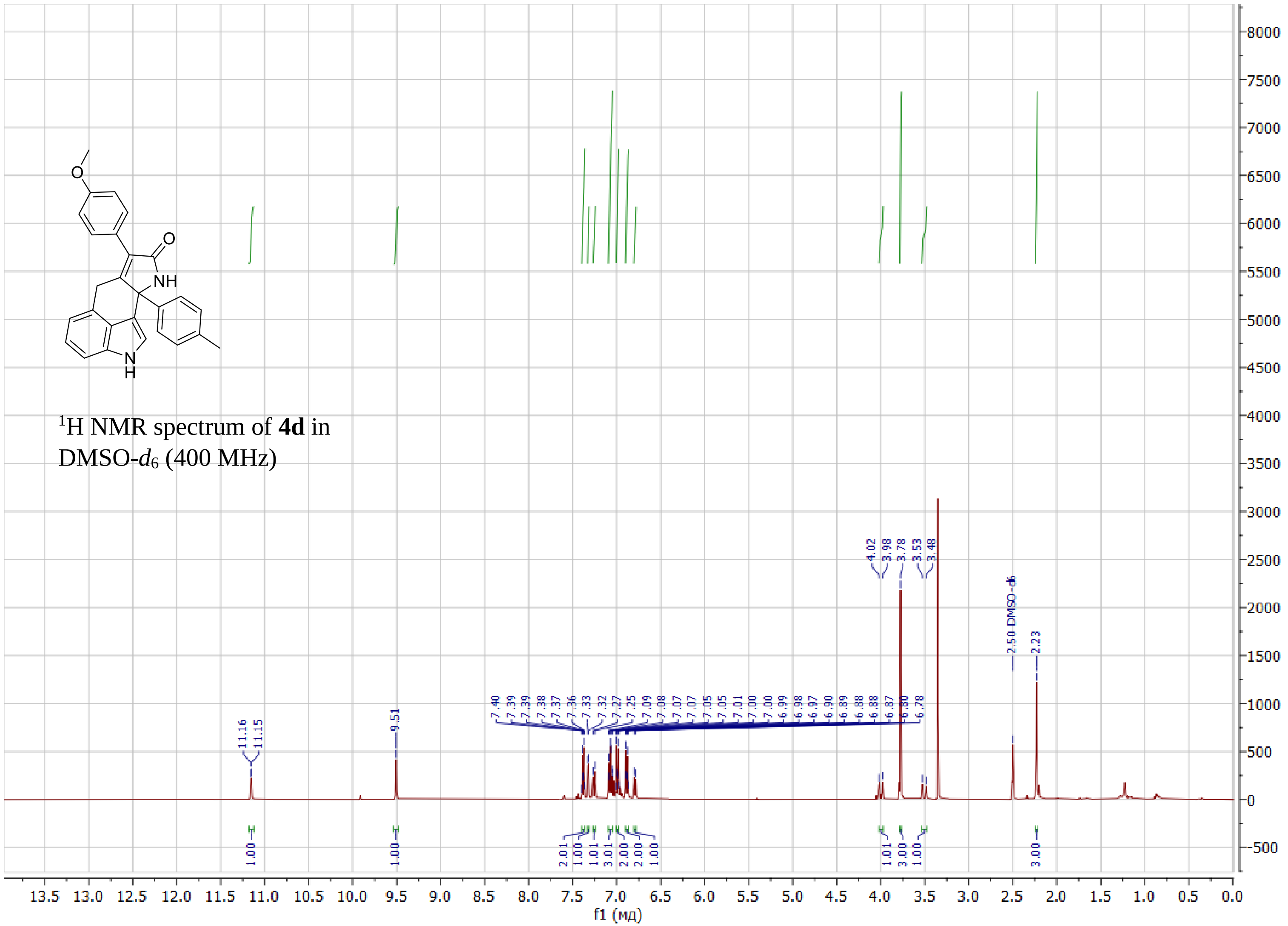

Figure S7. ^{1}H NMR spectrum of **4d** in DMSO-d_6 (400 MHz)

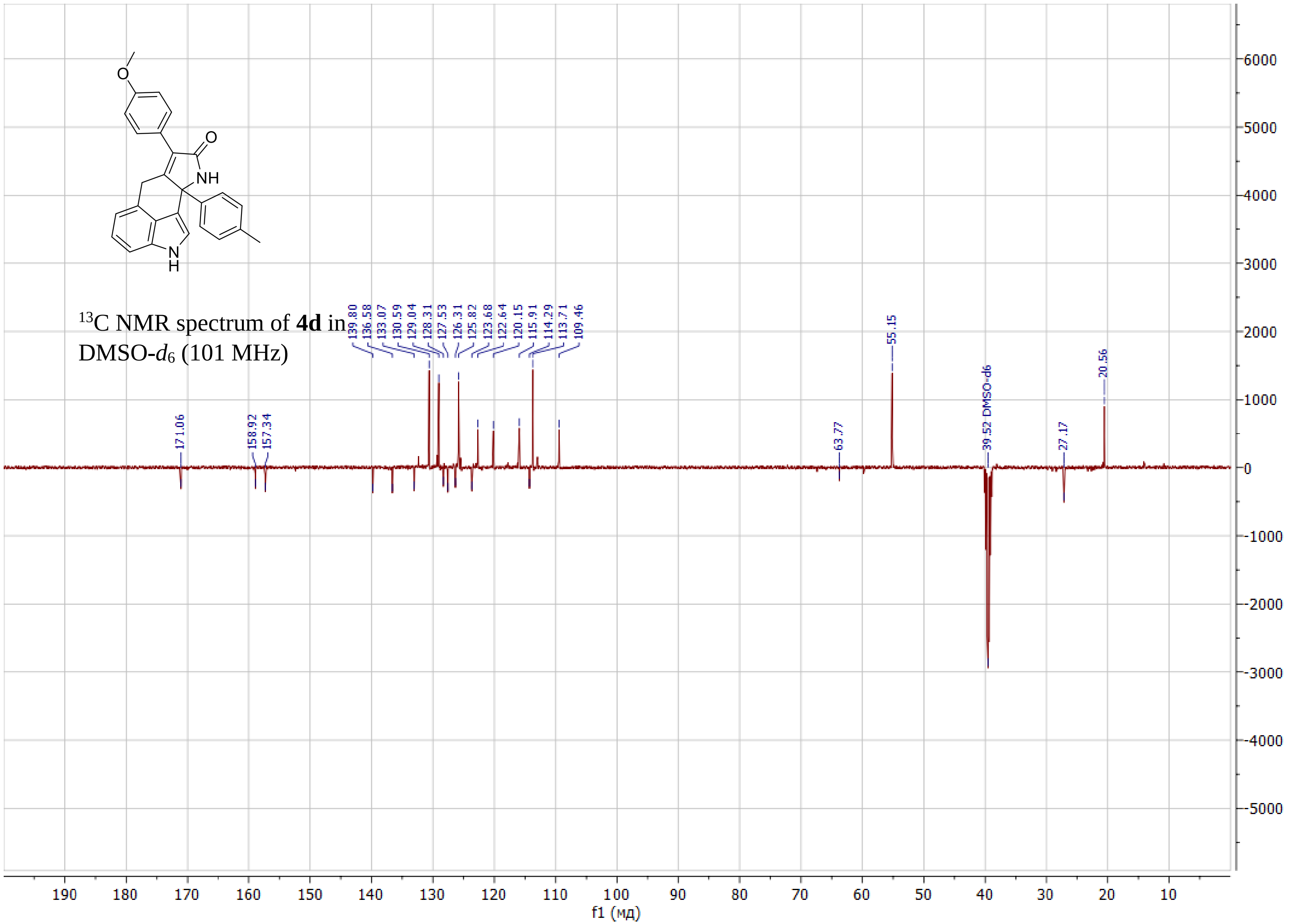

Figure S8. ^{13}C NMR spectrum of **4d** in DMSO-d_6 (101 MHz)

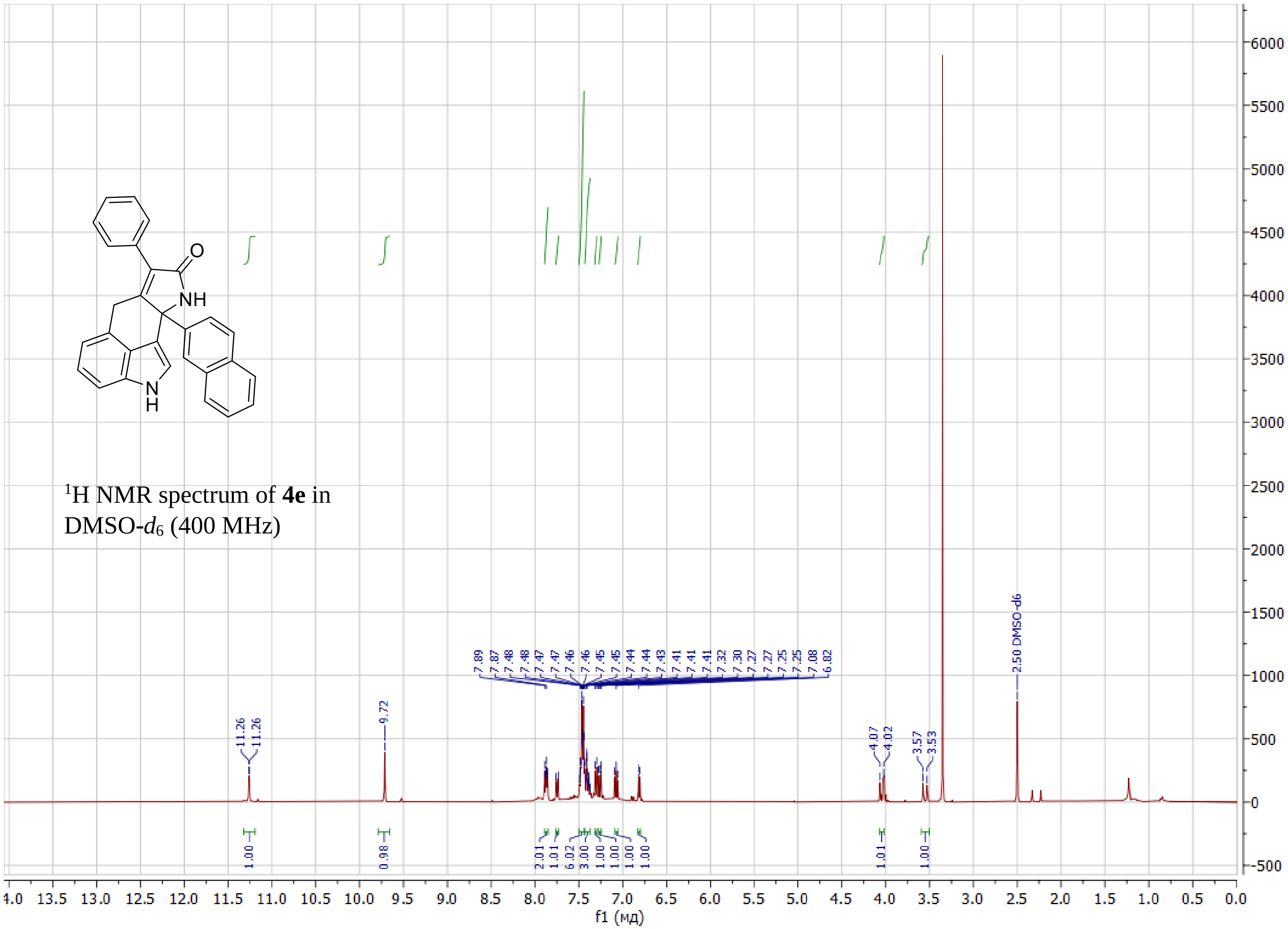

Figure S9.· ^{1}H NMR spectrum of **4e** in DMSO-d_6 (400 MHz)

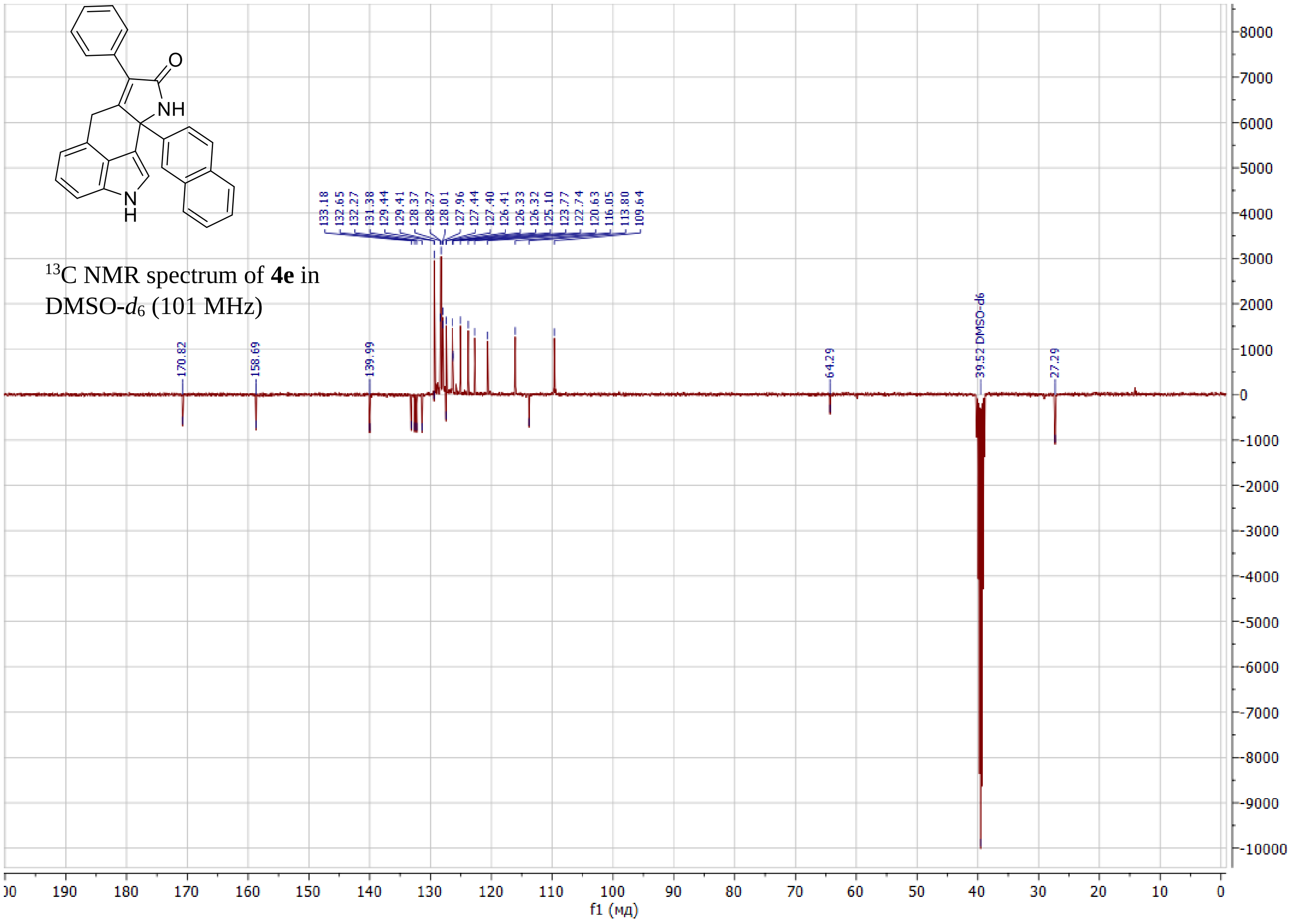

Figure S10. ^{13}C NMR spectrum of **4e** in DMSO-d_6 (101 MHz)

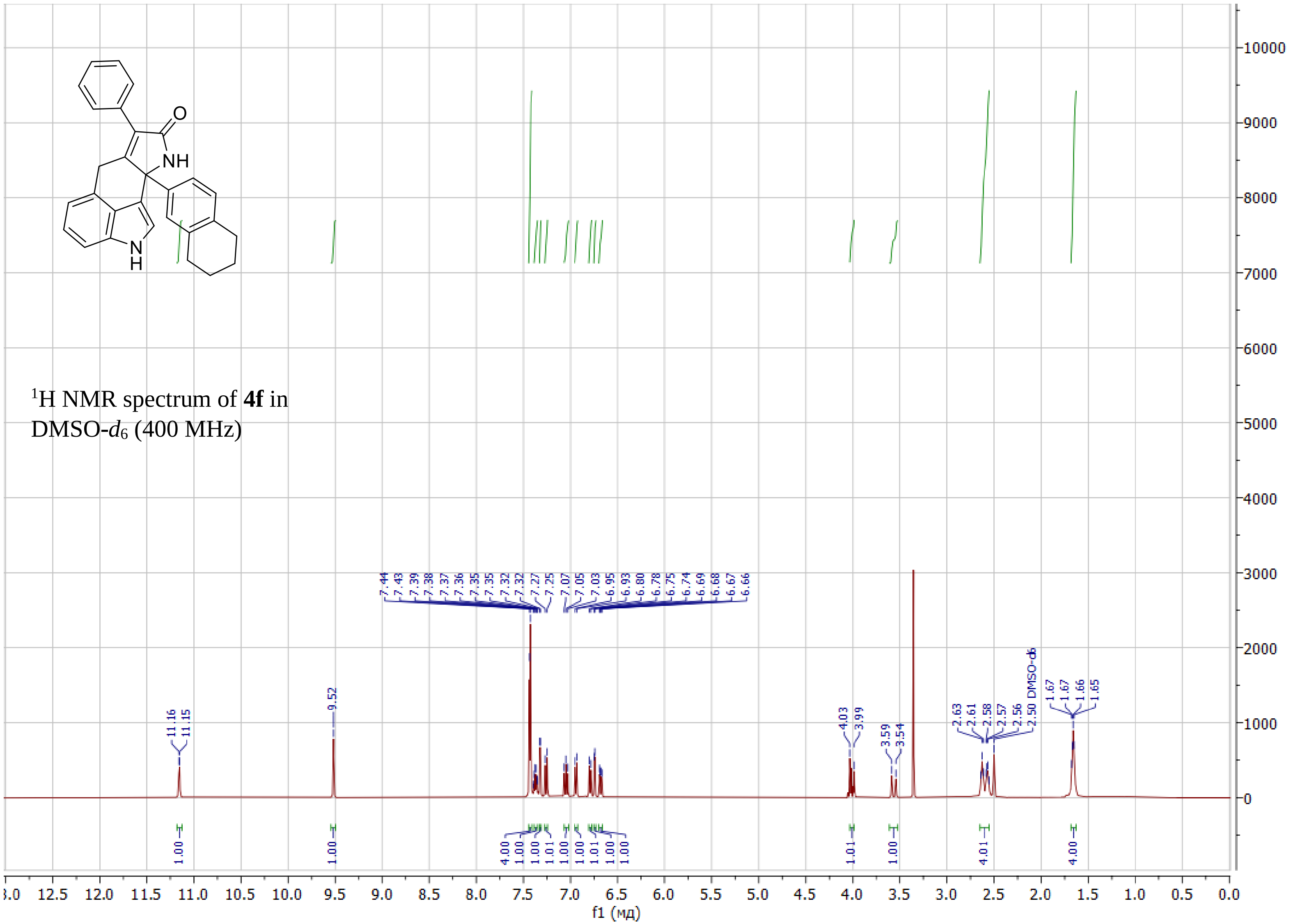

Figure S11. ^{1}H NMR spectrum of **4f** in DMSO-d_6 (400 MHz)

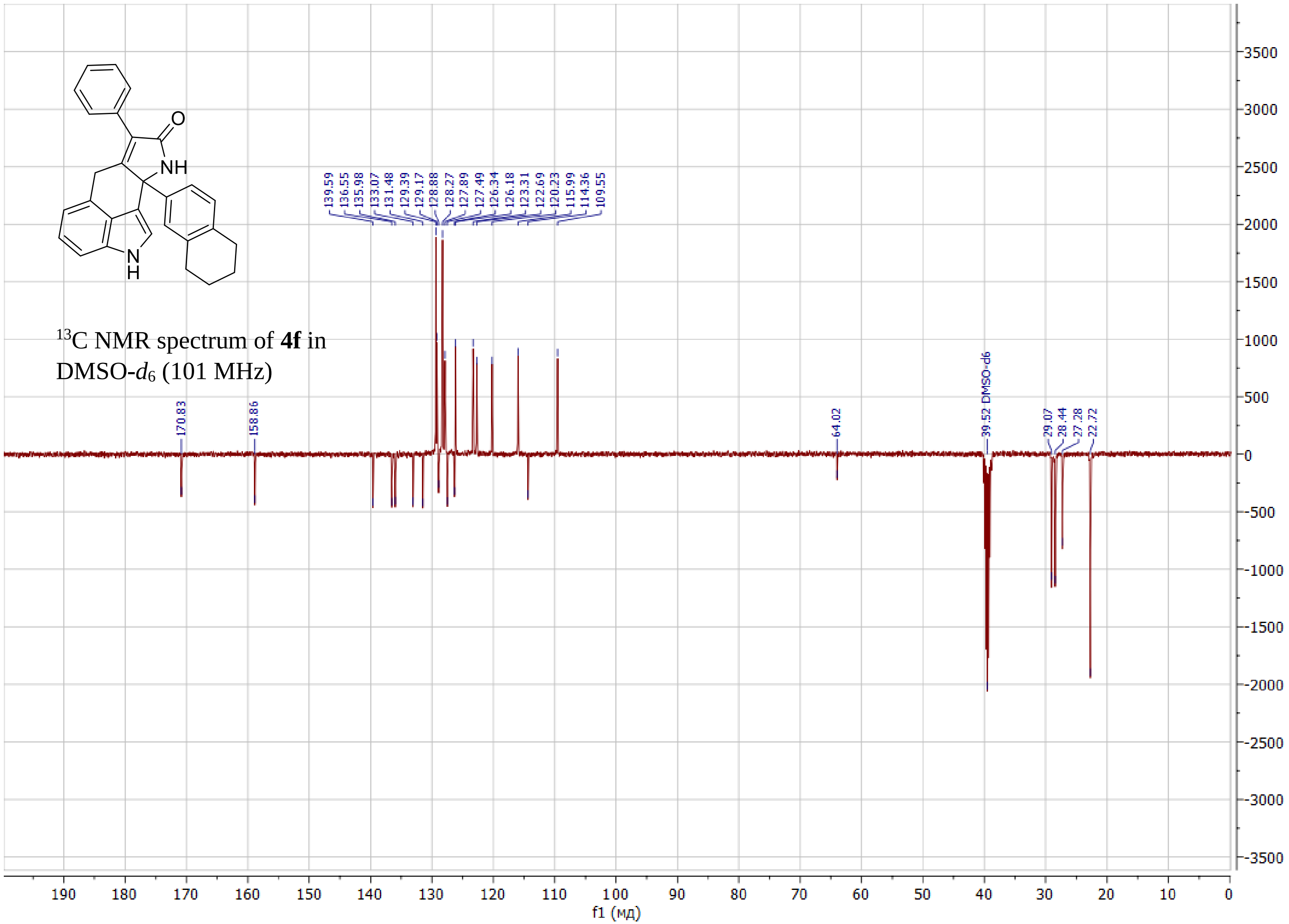

Figure S12. ^{13}C NMR spectrum of **4f** in DMSO-d_6 (101 MHz)

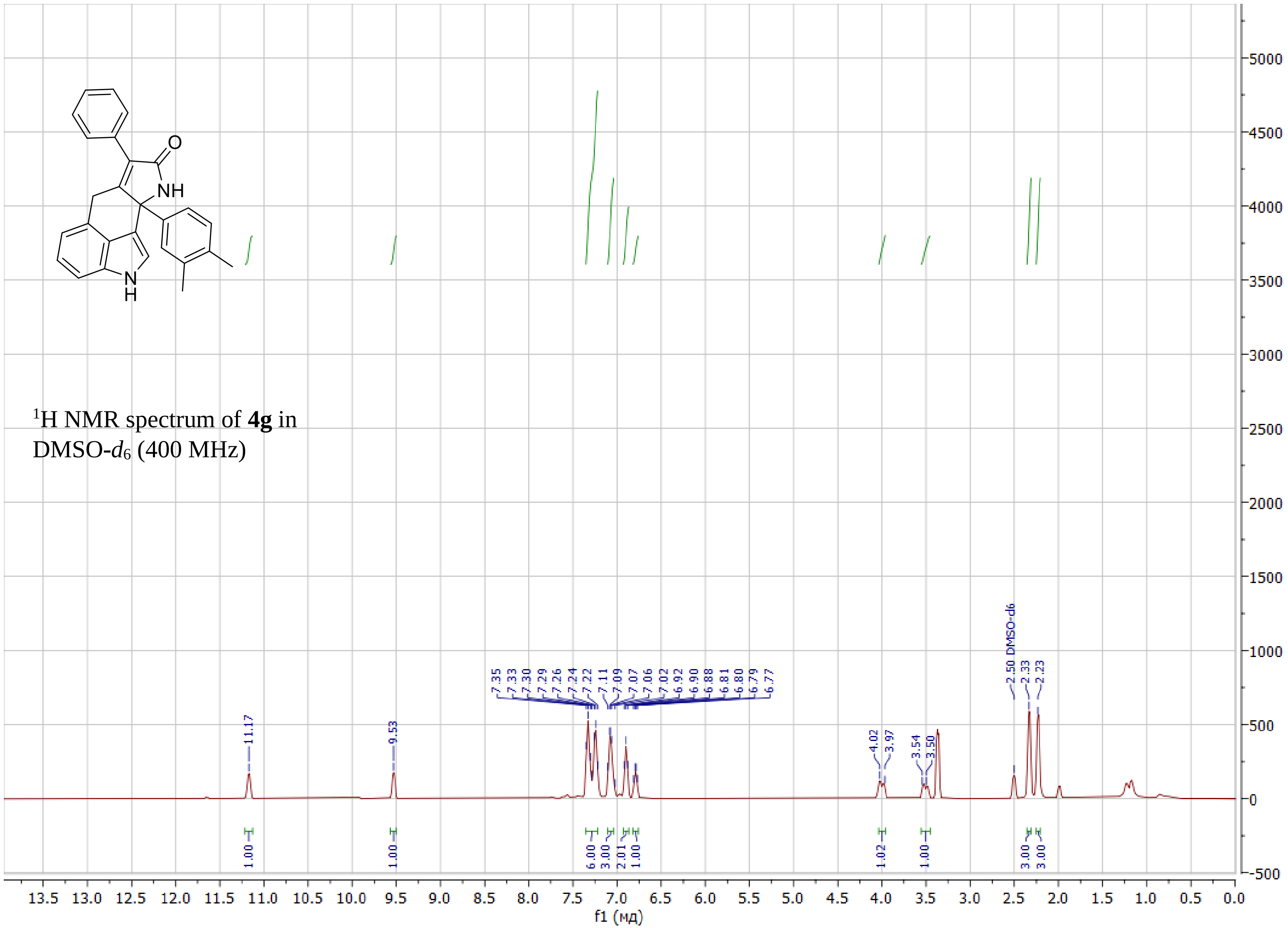

Figure S13. ^{1}H NMR spectrum of **4g** in DMSO-d_6 (400 MHz)

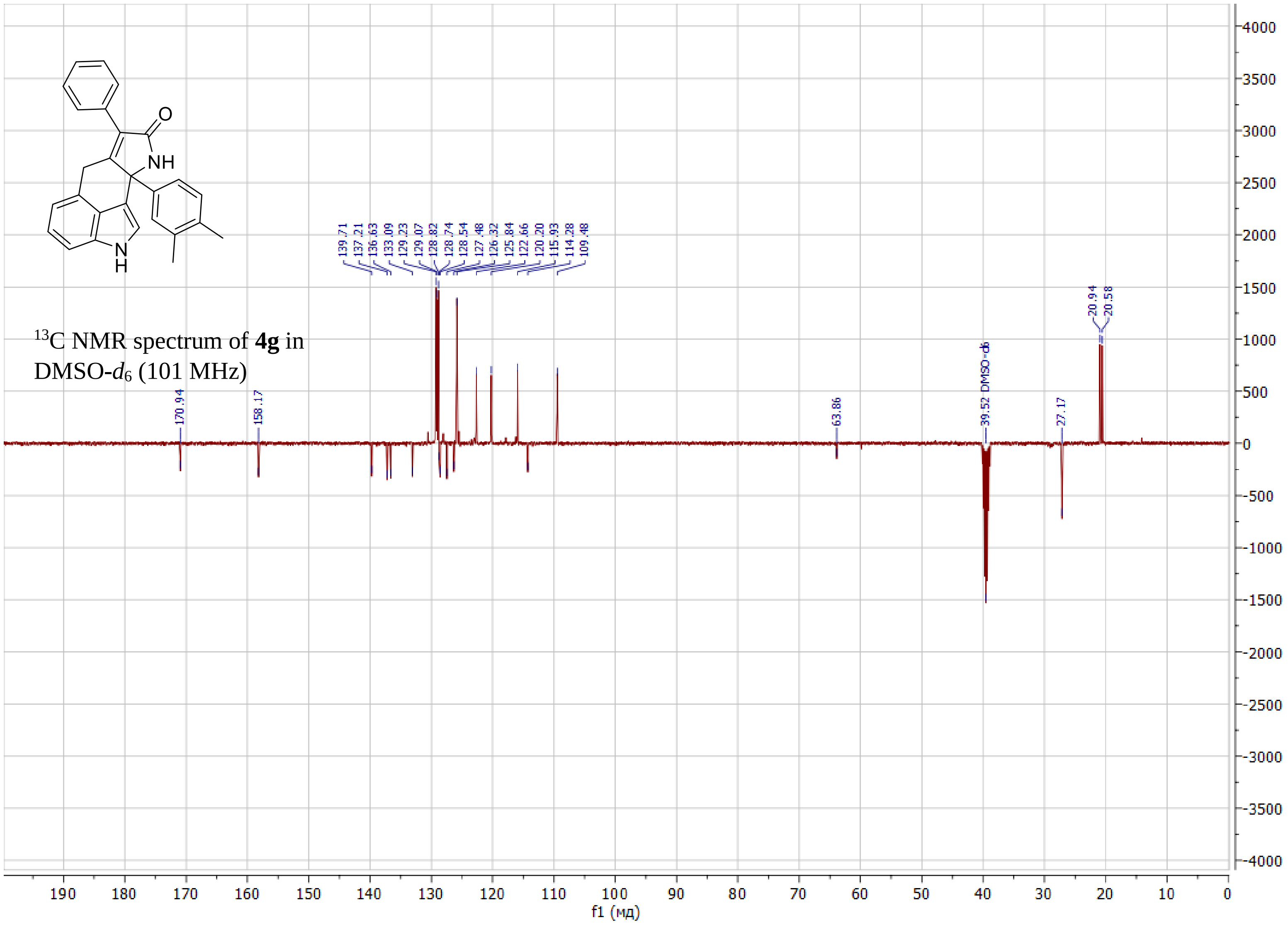

Figure S14. ^{13}C NMR spectrum of **4g** in DMSO-d_6 (101 MHz)

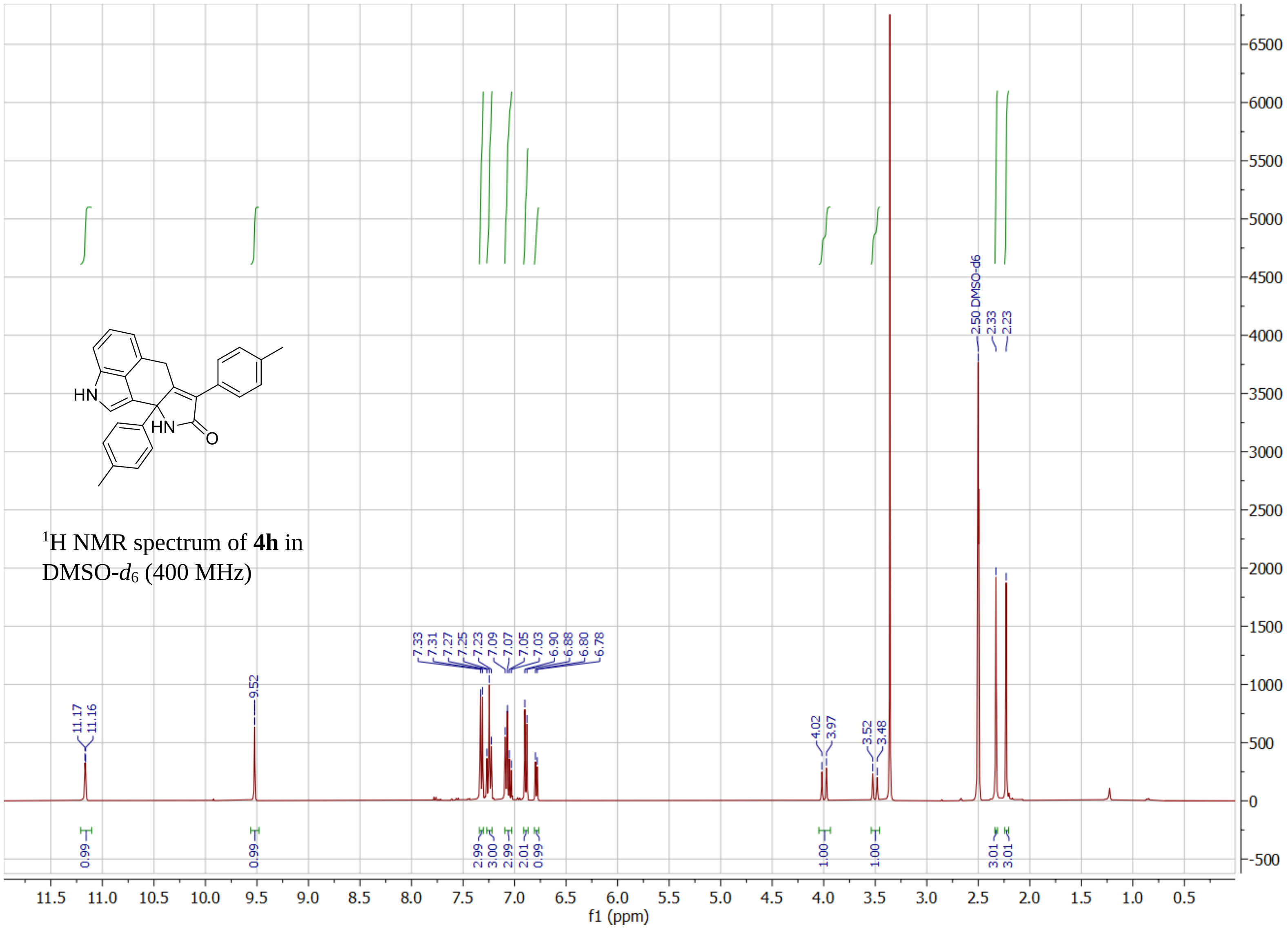

Figure S15. ^{1}H NMR spectrum of **4h** in DMSO-d_6 (400 MHz)

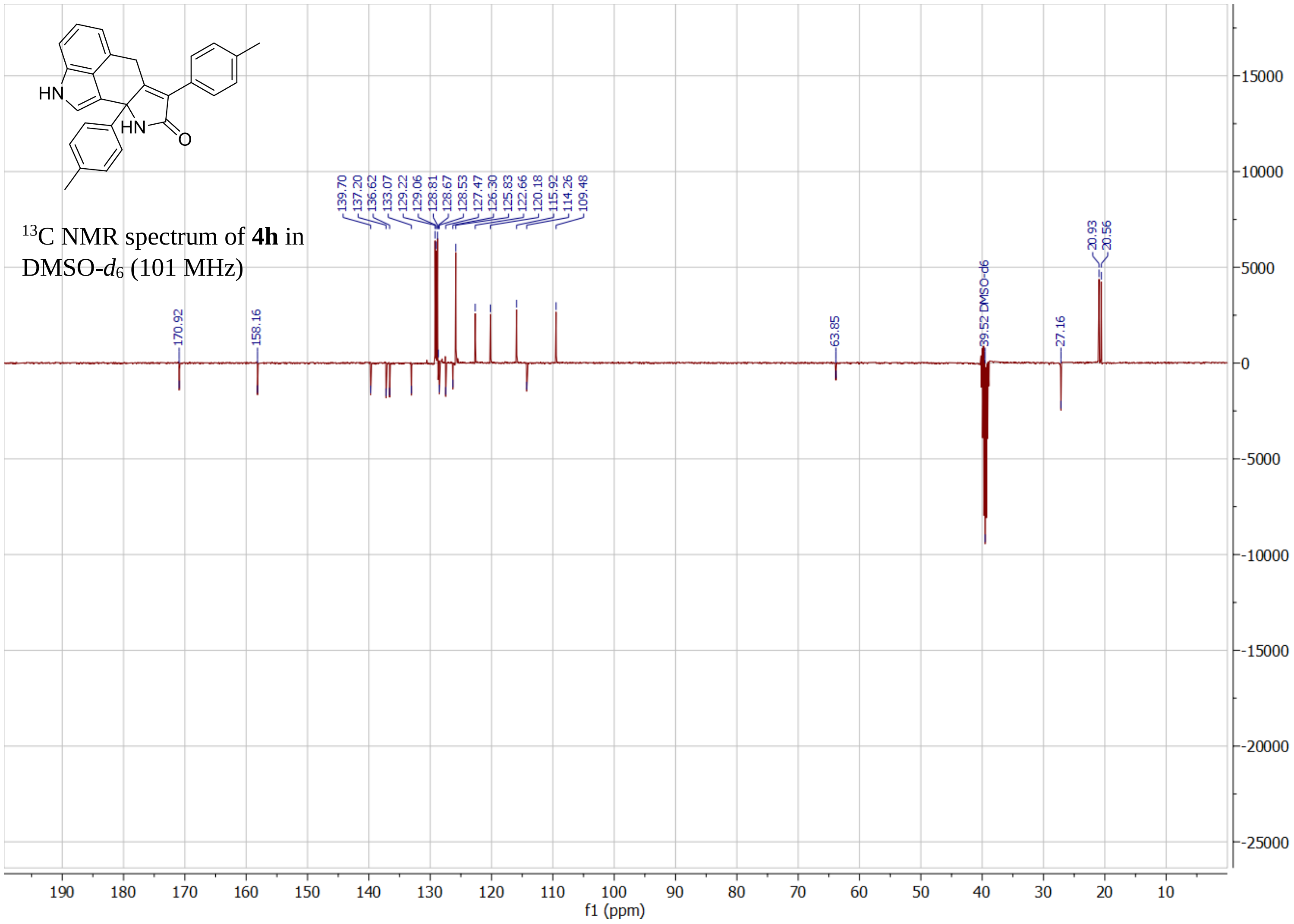

Figure S16 ^{13}C NMR spectrum of **4h** in DMSO-d_6 (101 MHz)

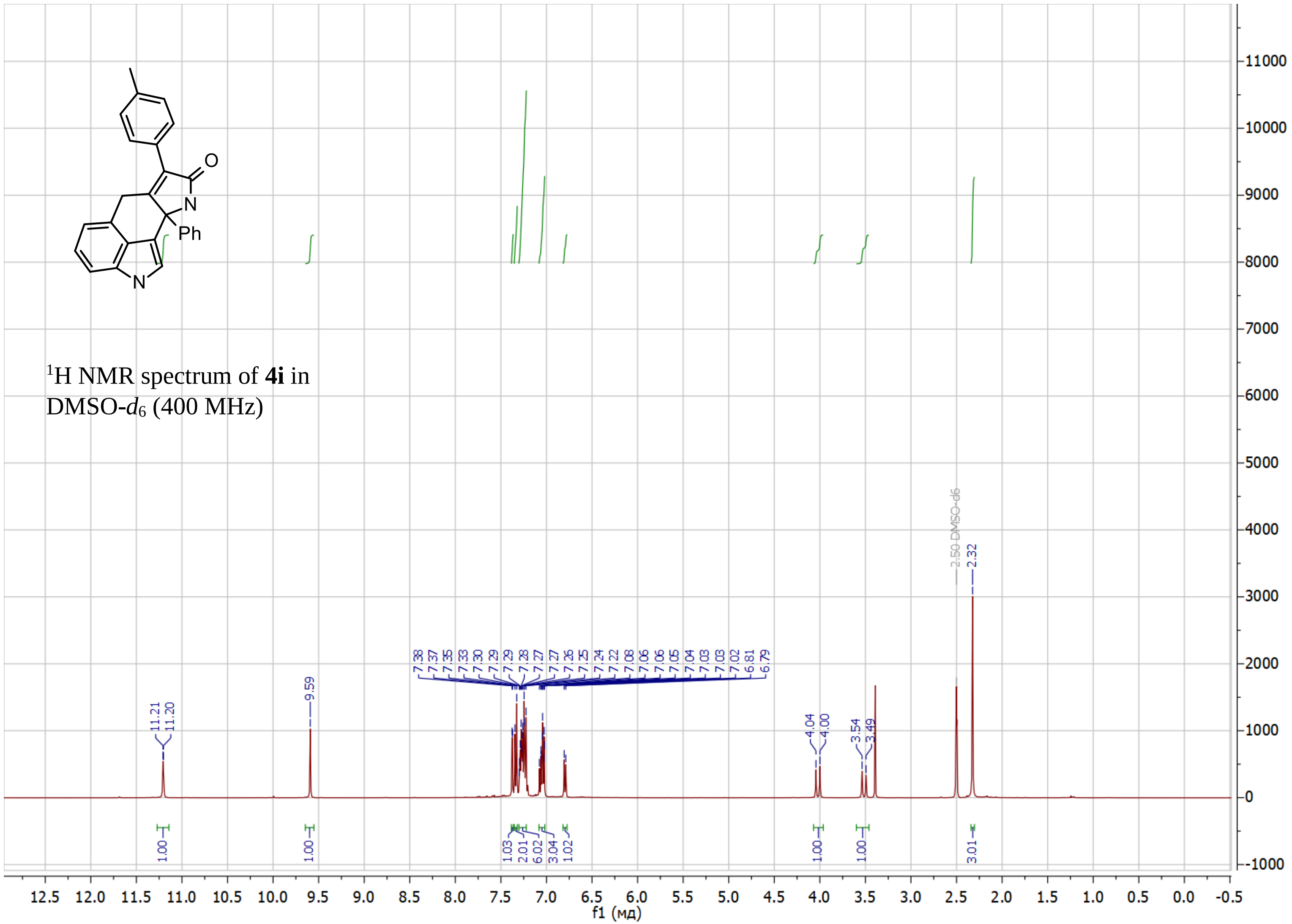

Figure S17 ^{1}H NMR spectrum of **4i** in DMSO-d_6 (400 MHz)

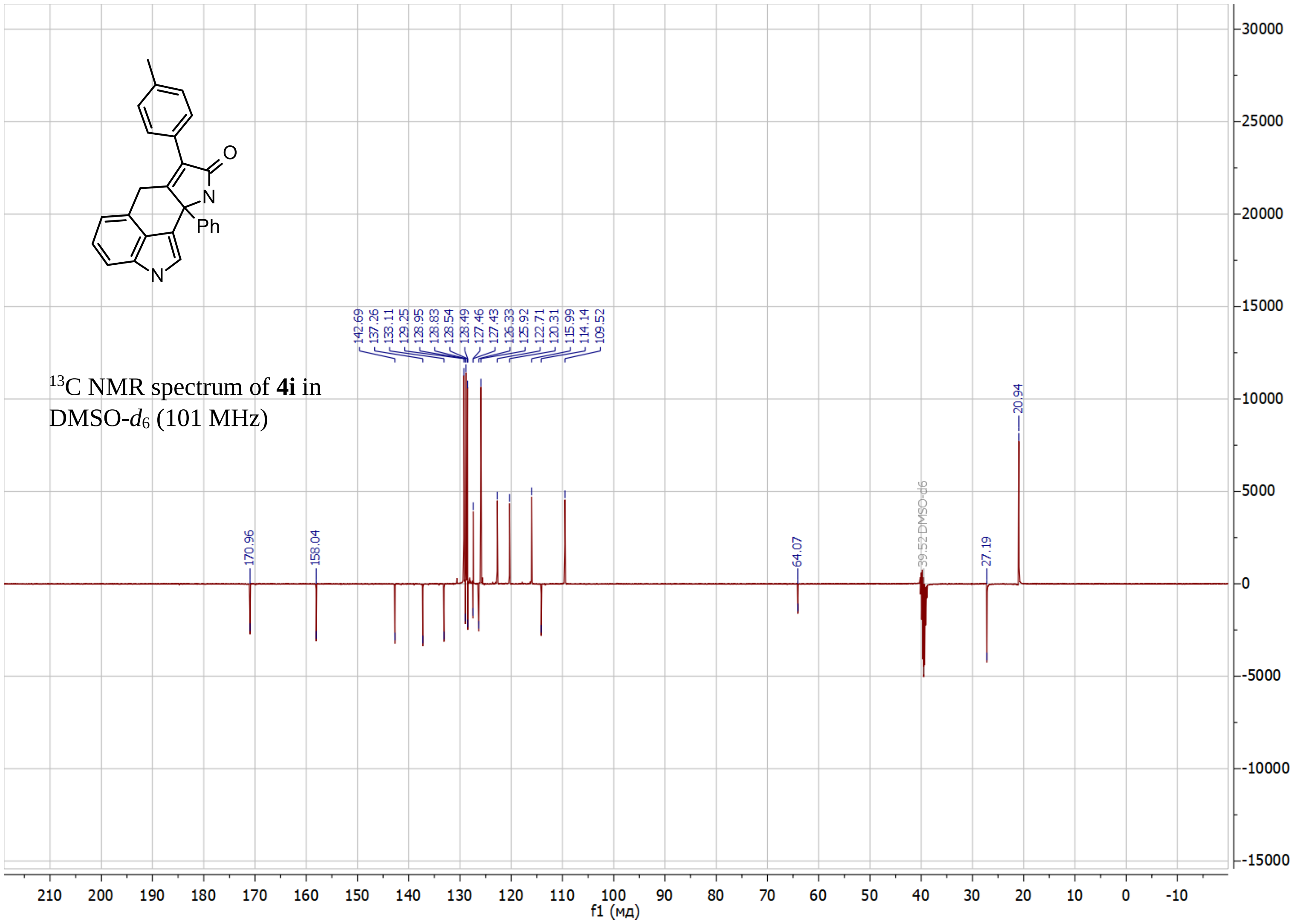

Figure S18. ^{13}C NMR spectrum of **4i** in DMSO-d_6 (101 MHz)

^{1}H and ^{13}C NMR spectral charts for 5-(1H-indol-3-yl)-3,5-diaryl-1,5-dihydro-2H-pyrrol-2-ones **7aa-ah,bd,ca,da,eg,fc,fg,ga**

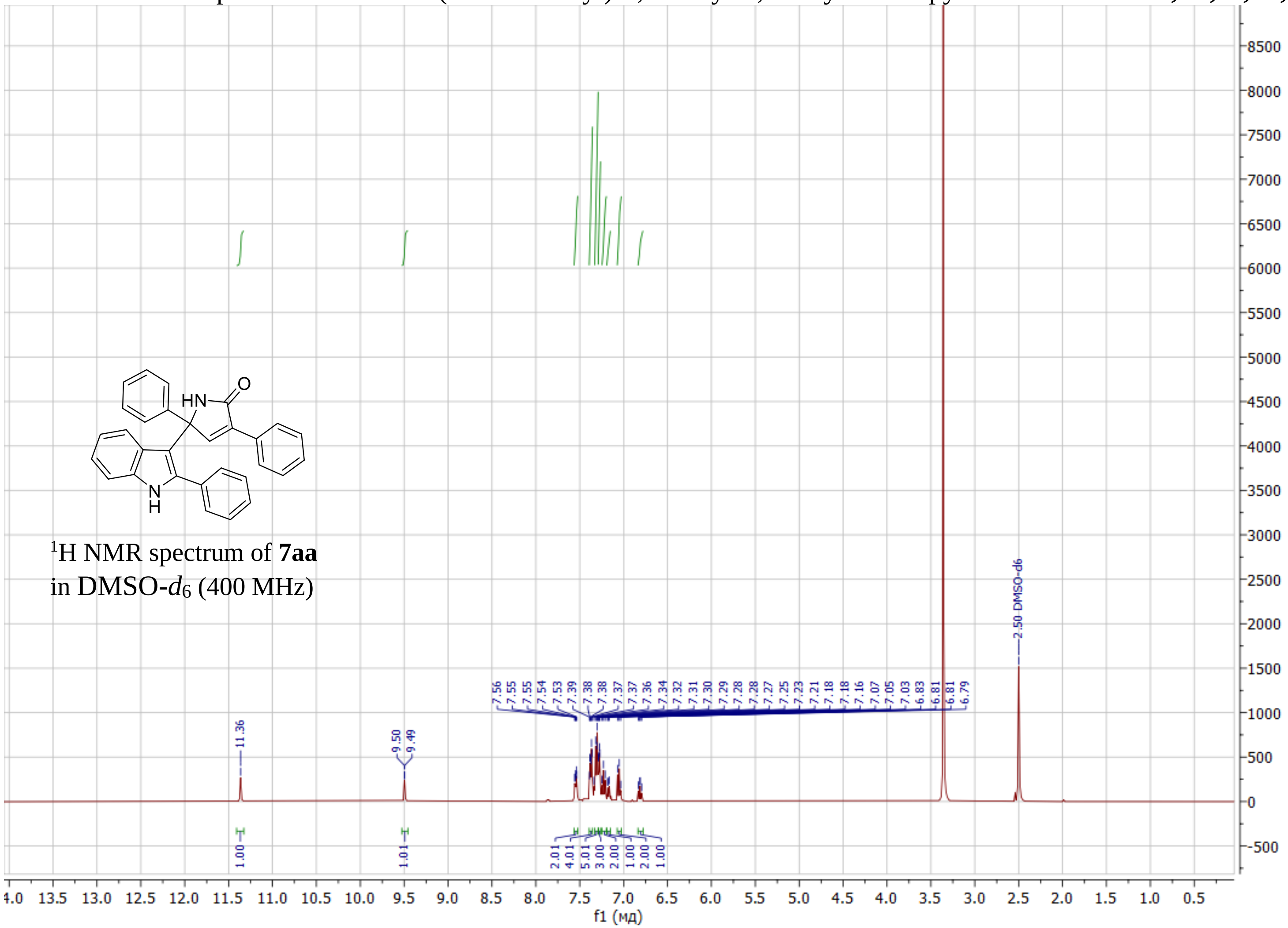

Figure S19. ^{1}H NMR spectrum of **7aa** in DMSO-d_6 (400 MHz)

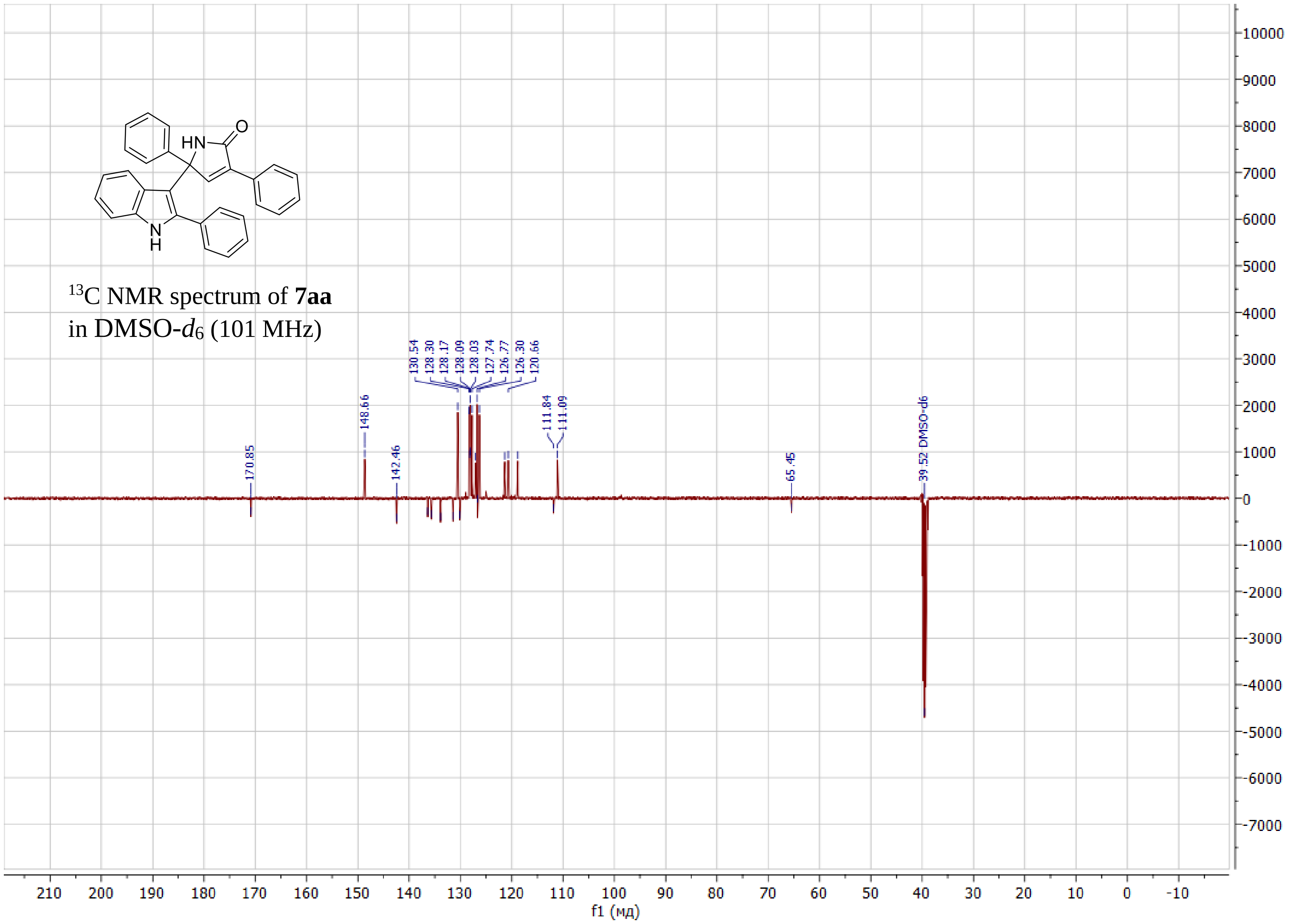

Figure S20. ^{13}C NMR spectrum of **7aa** in DMSO-d_6 (101 MHz)

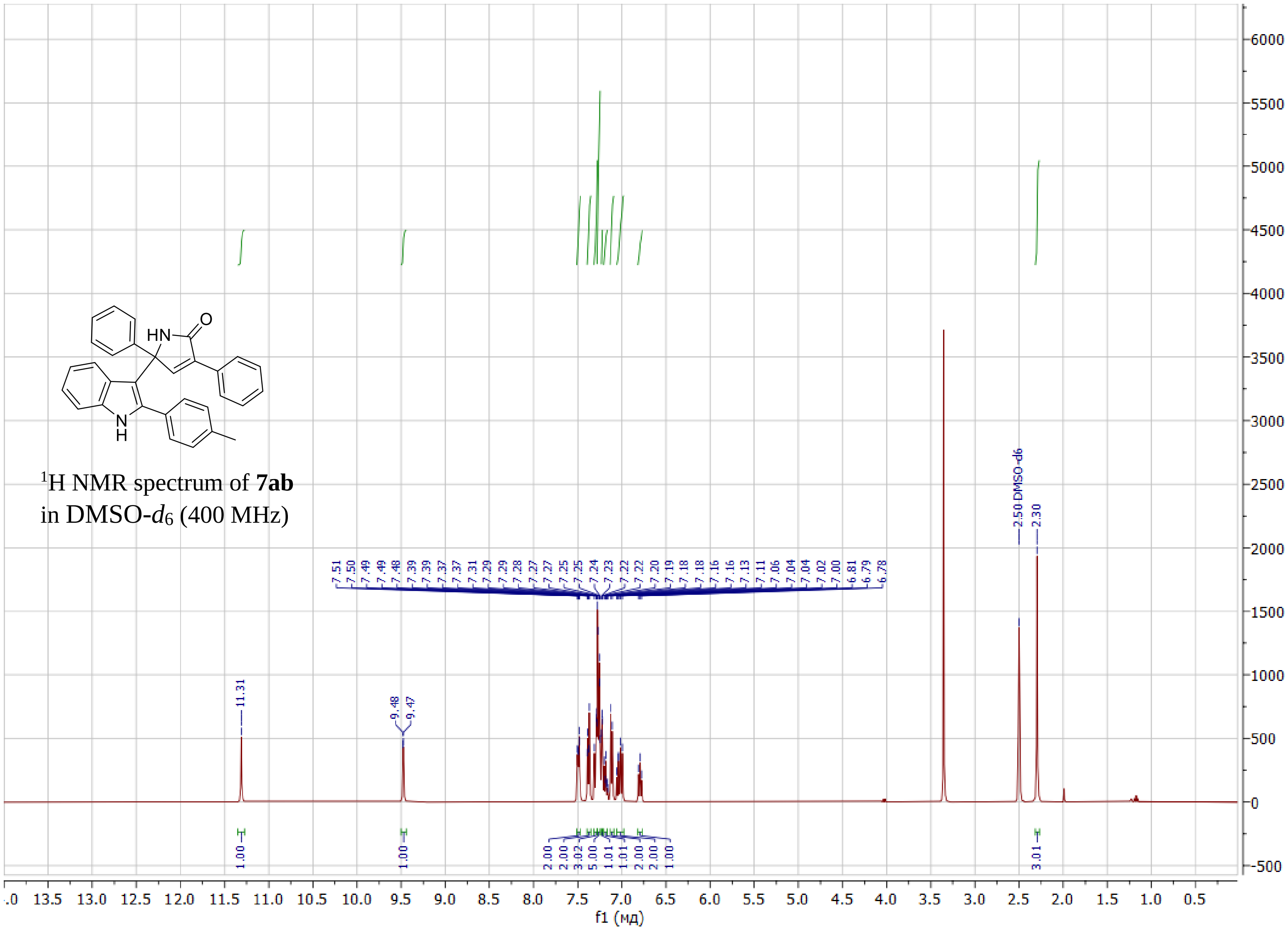

Figure S21. ^{1}H NMR spectrum of **7ab** in DMSO-d_6 (400 MHz)

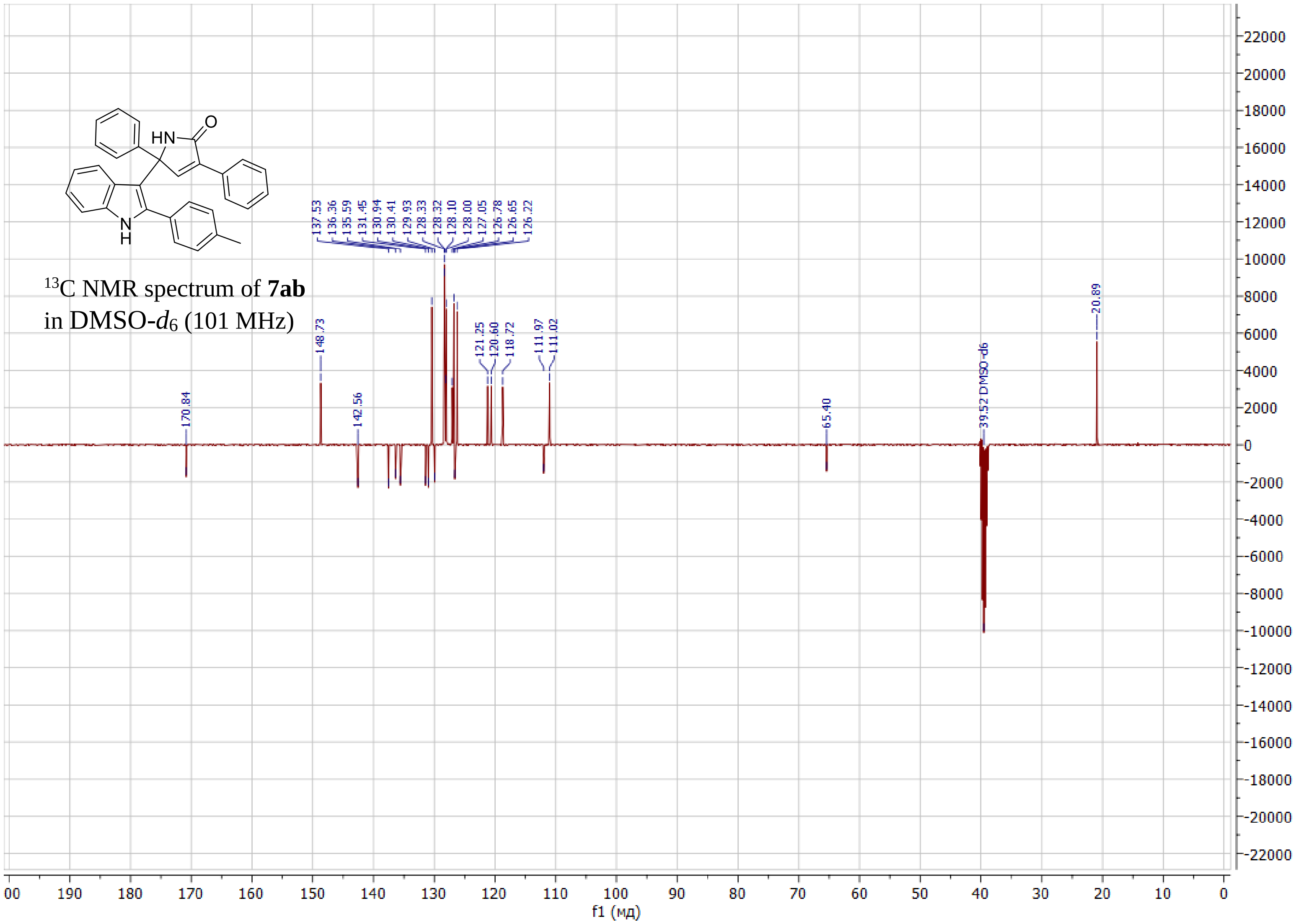

Figure S22. ^{13}C NMR spectrum of **7ab** in DMSO-d_6 (101 MHz)

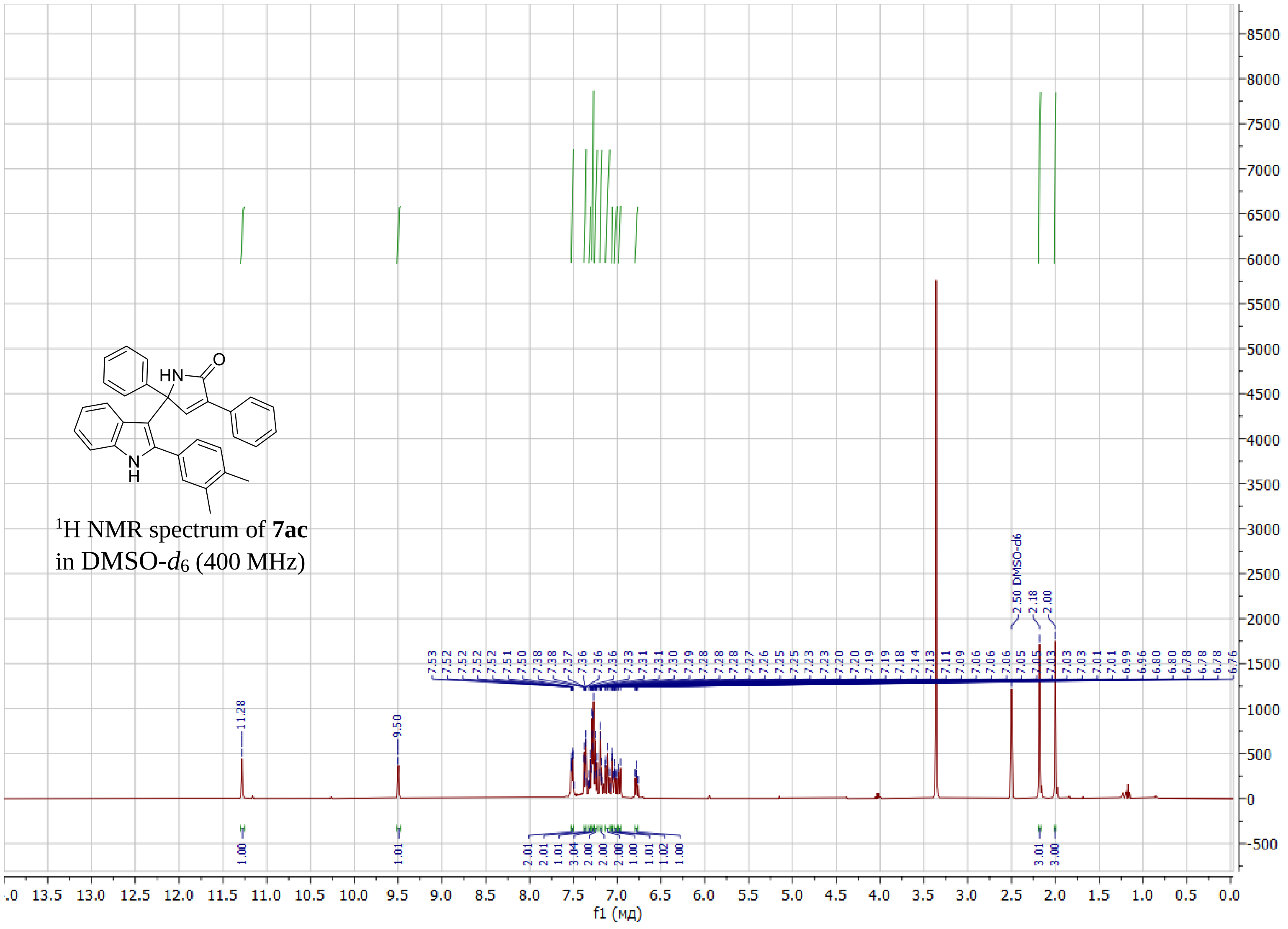

Figure S23. ^{1}H NMR spectrum of **7ac** in DMSO-d_6 (400 MHz)

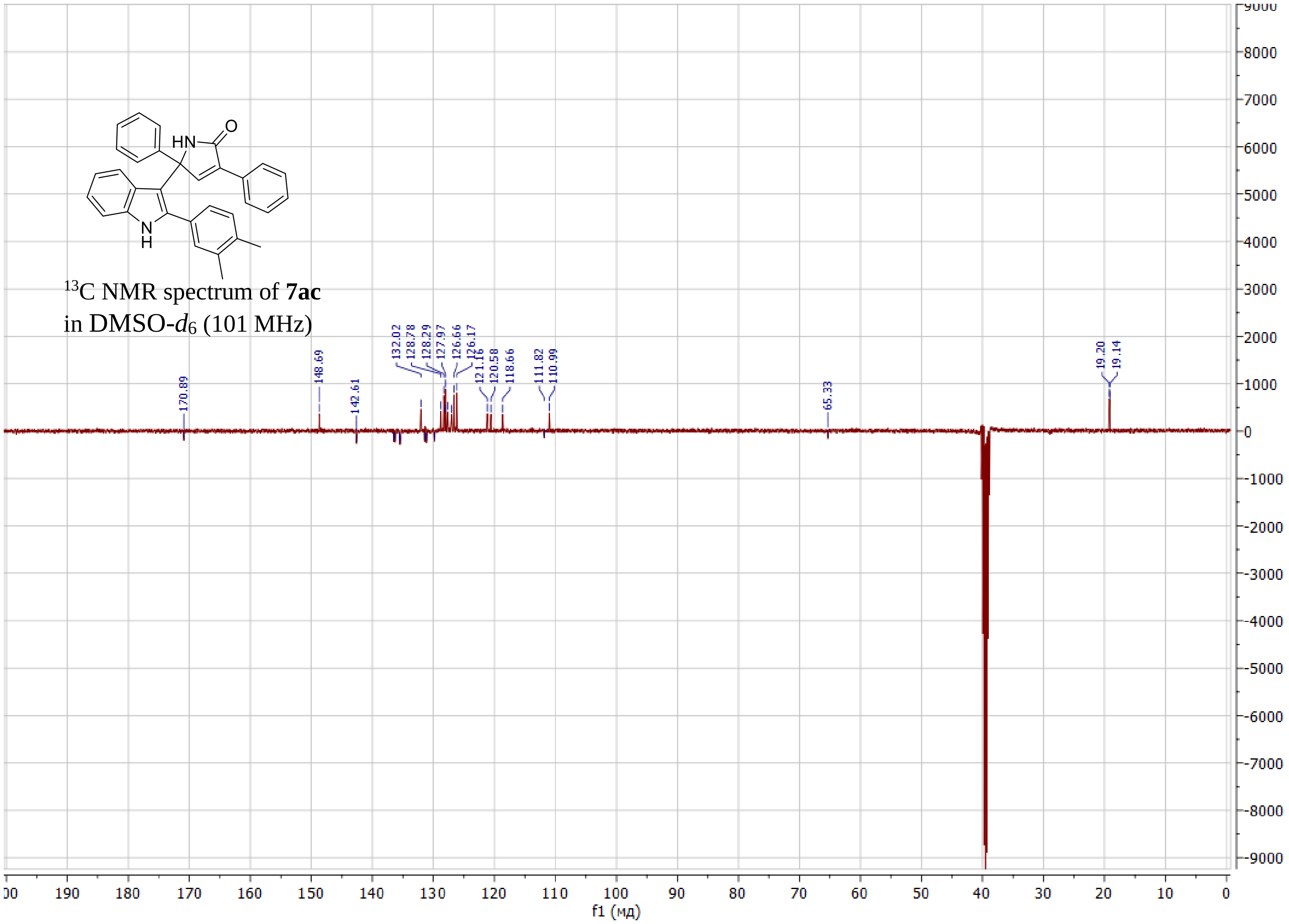

Figure S24. ^{13}C NMR spectrum of **7ac** in DMSO-d_6 (101 MHz)

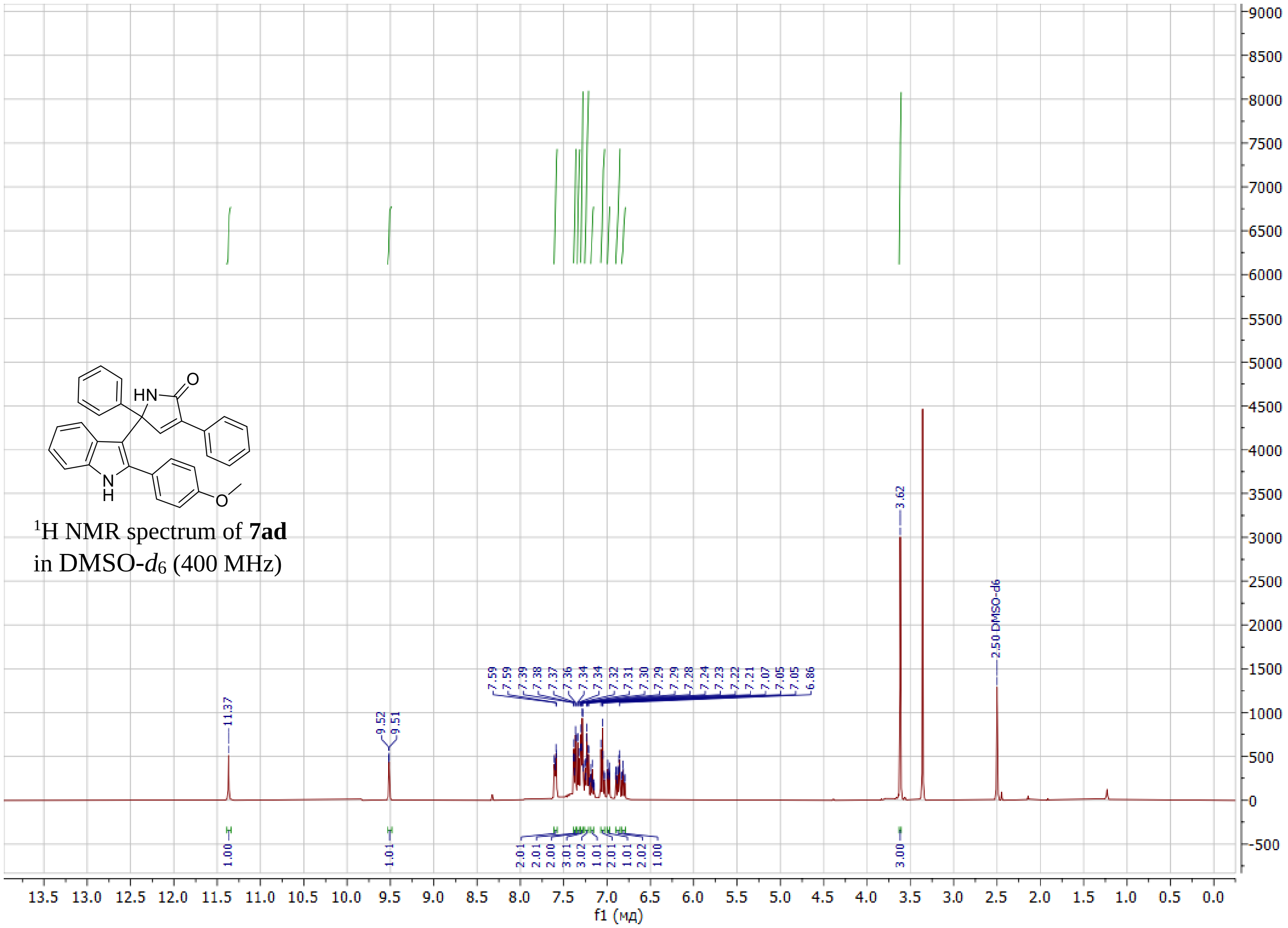

Figure S25. ^{1}H NMR spectrum of **7ad** in DMSO-d_6 (400 MHz)

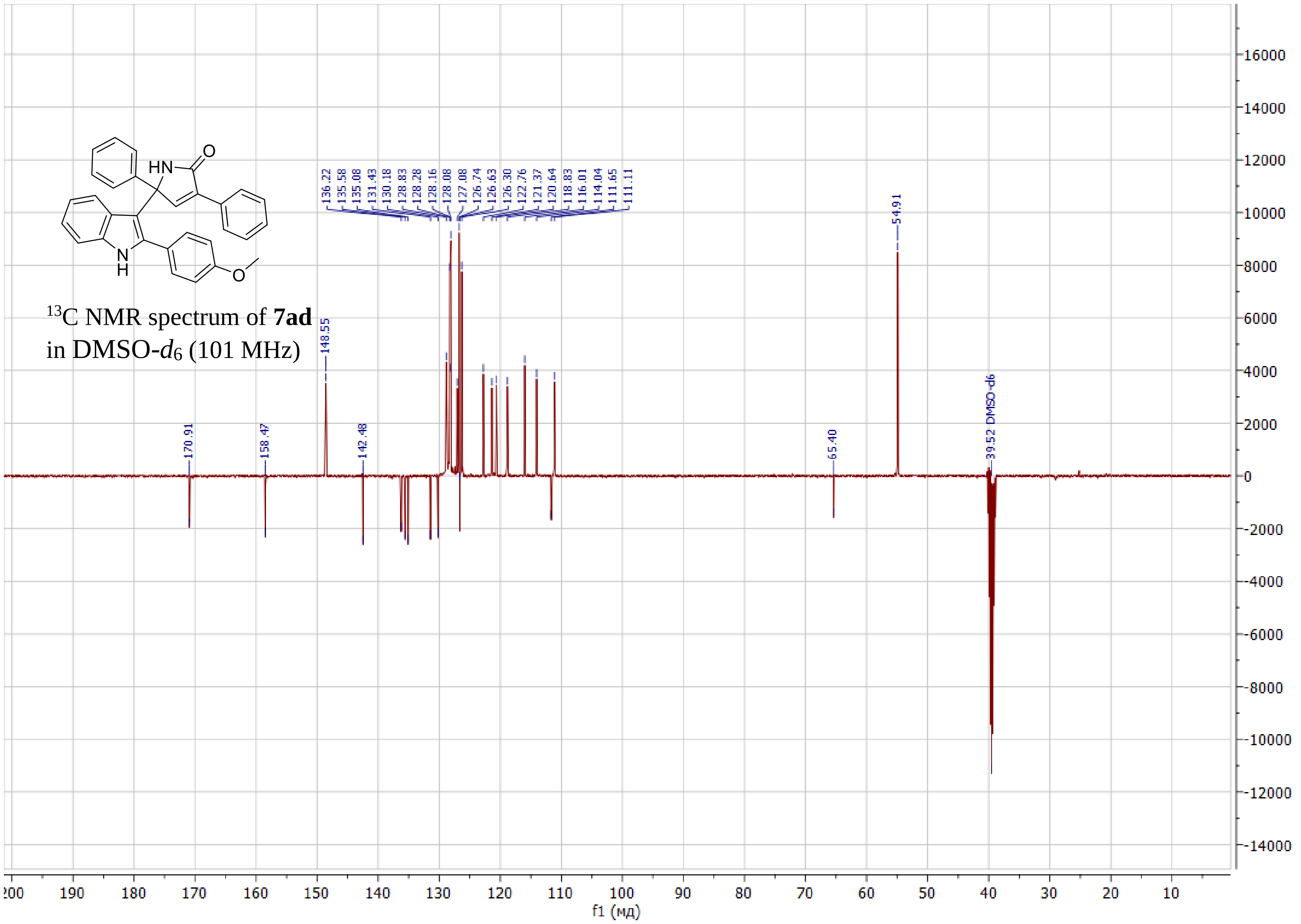

Figure S26. ^{13}C NMR spectrum of **7ad** in DMSO-d_6 (101 MHz)

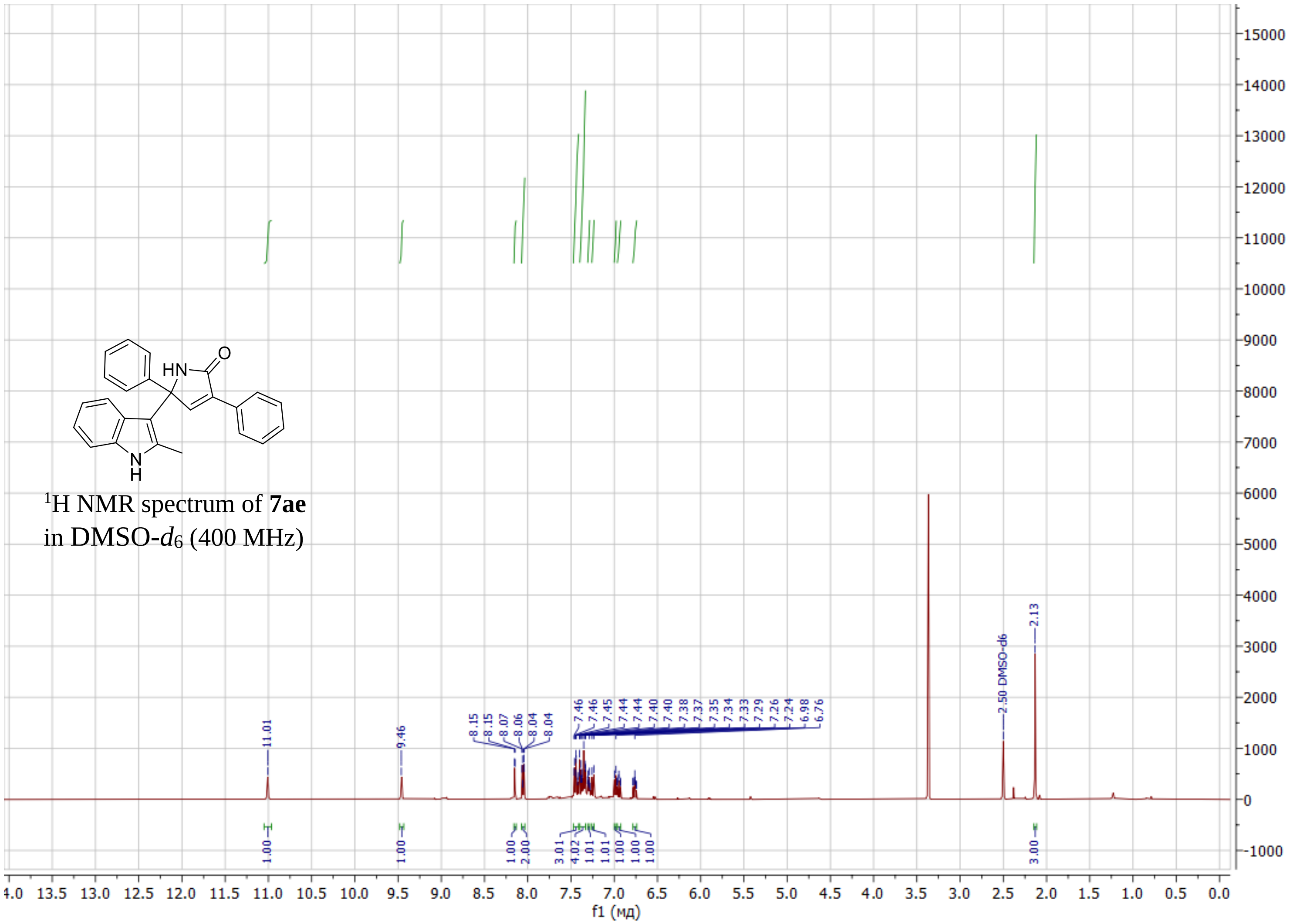

Figure S27. ^{1}H NMR spectrum of **7ae** in DMSO-d_6 (400 MHz)

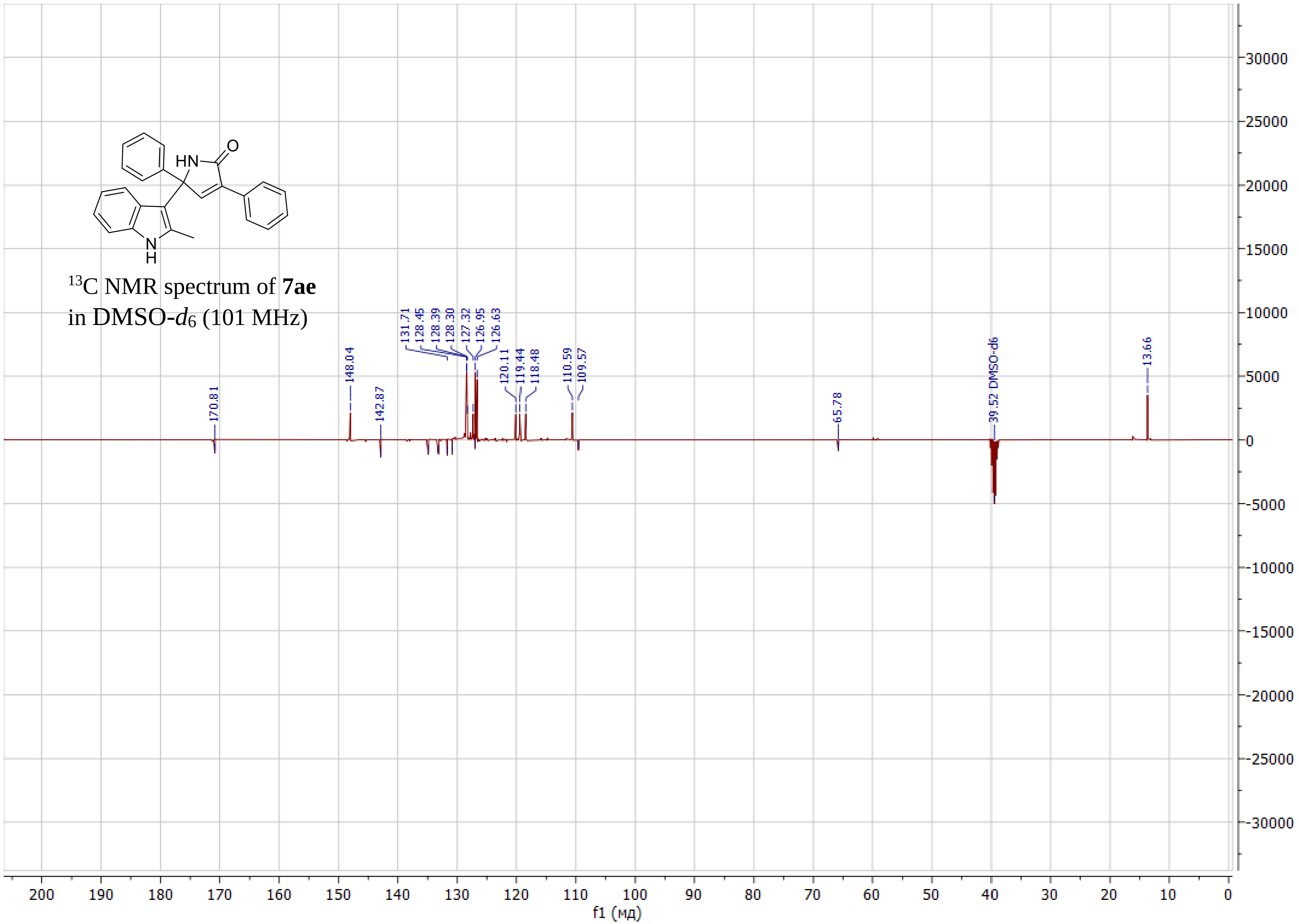

Figure S28. ^{13}C NMR spectrum of **7ae** in DMSO-d_6 (101 MHz)

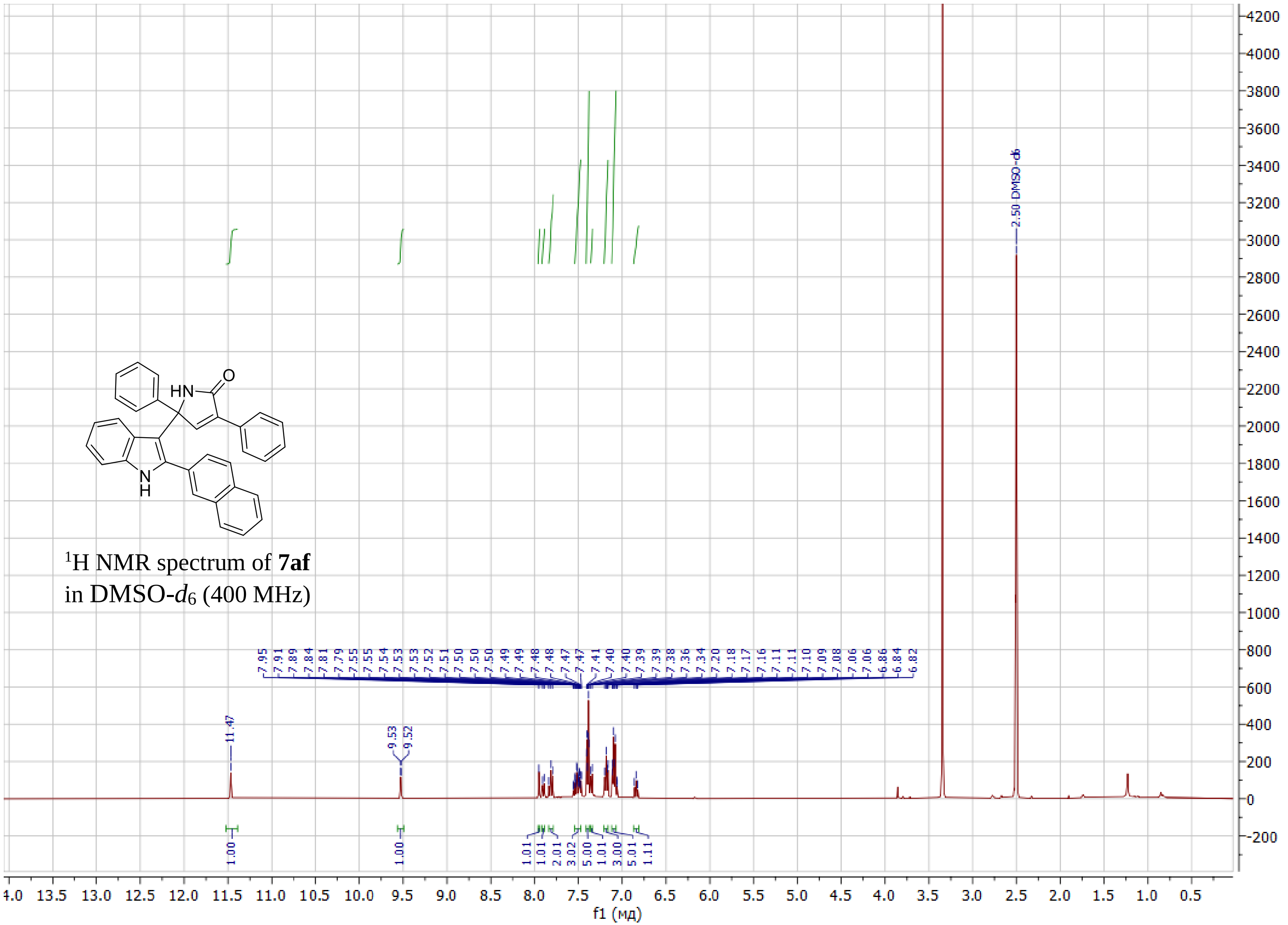

Figure S29. ^{1}H NMR spectrum of **7af** in DMSO-d_6 (400 MHz)

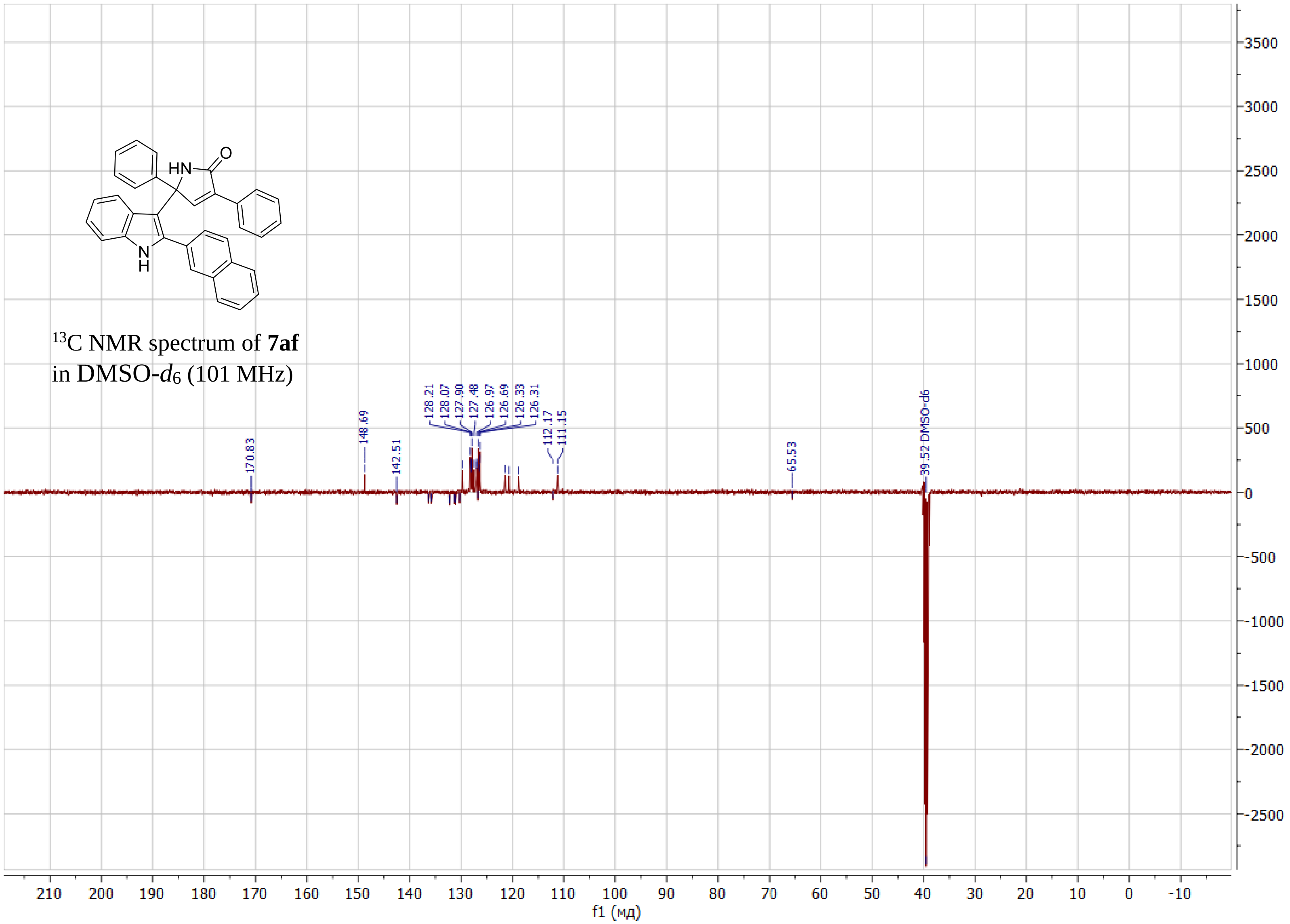

Figure S30. ^{13}C NMR spectrum of **7af** in DMSO-d_6 (101 MHz)

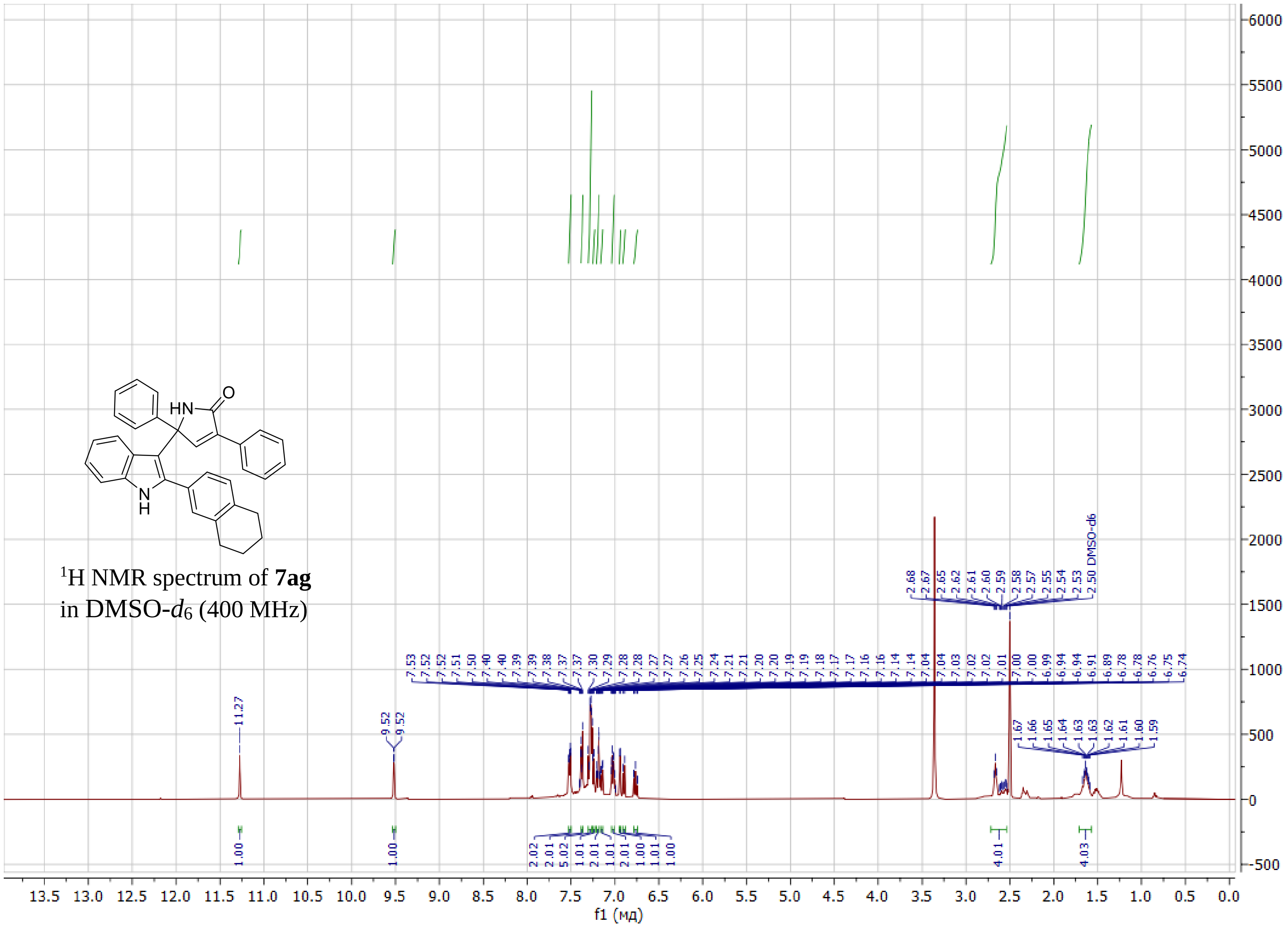

Figure S31. ^{1}H NMR spectrum of **7ag** in DMSO-d_6 (400 MHz)

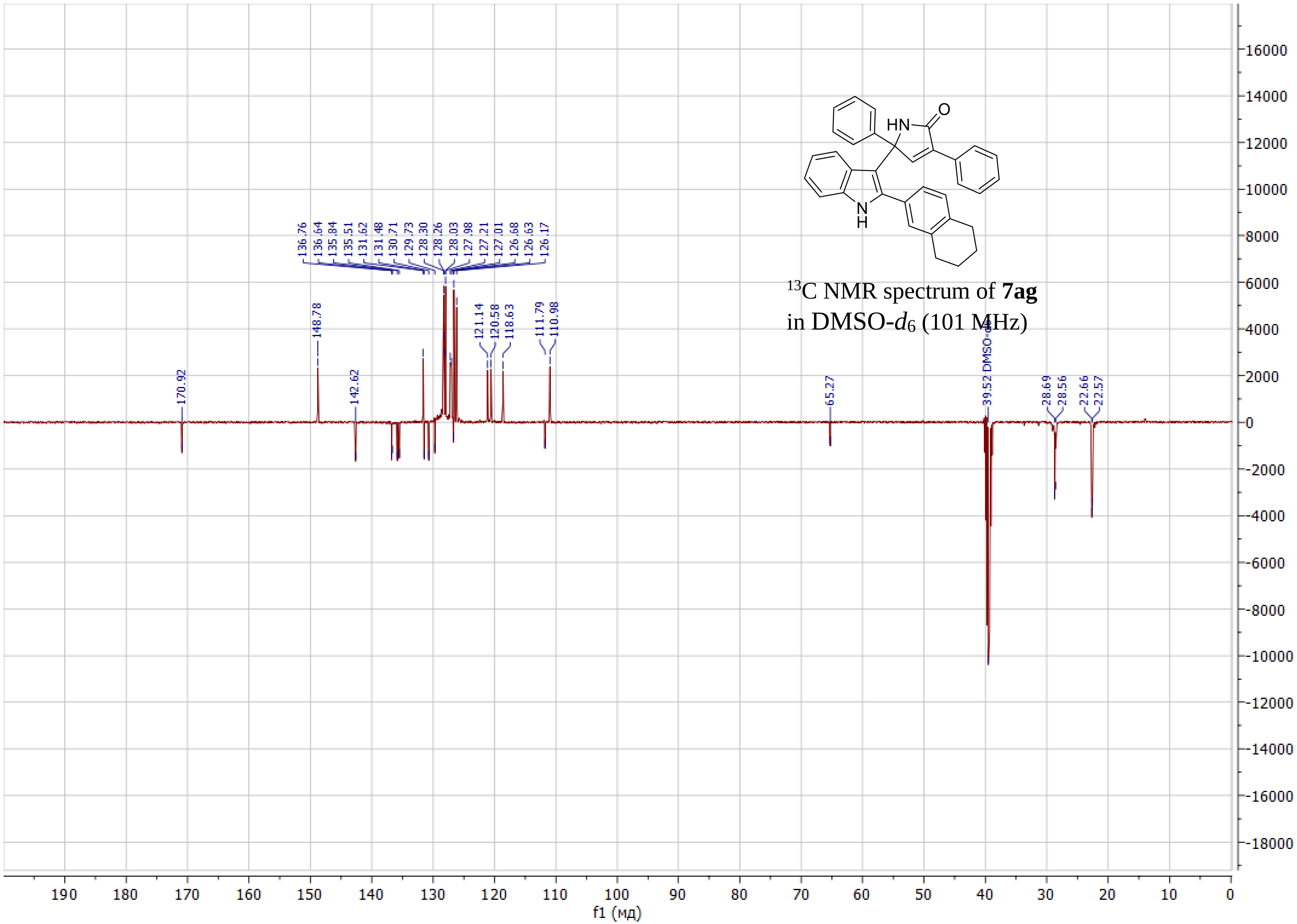

Figure S32. ^{13}C NMR spectrum of **7ag** in DMSO-d_6 (101 MHz)

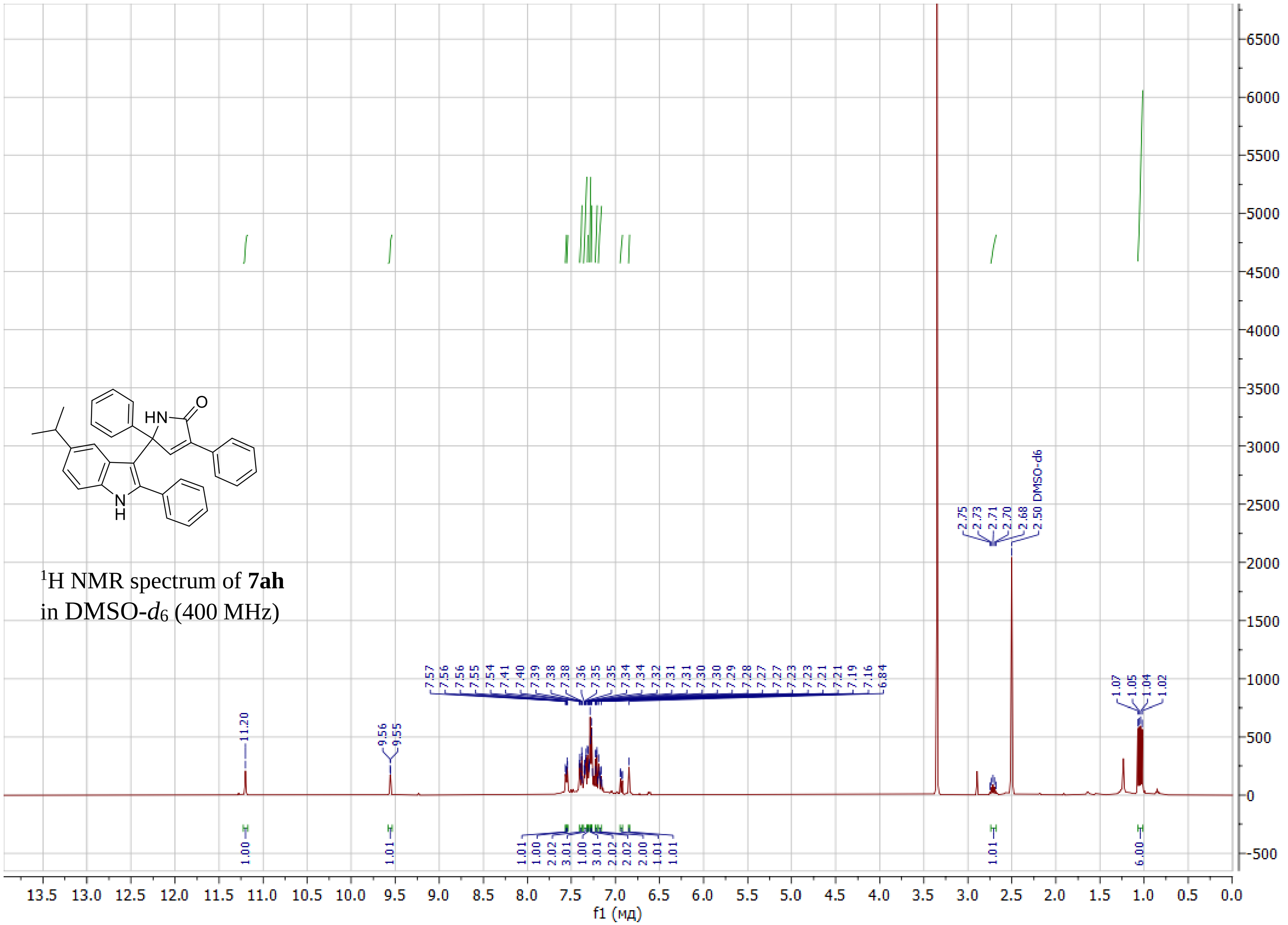

Figure S33. ^{1}H NMR spectrum of **7ah** in DMSO-d_6 (400 MHz)

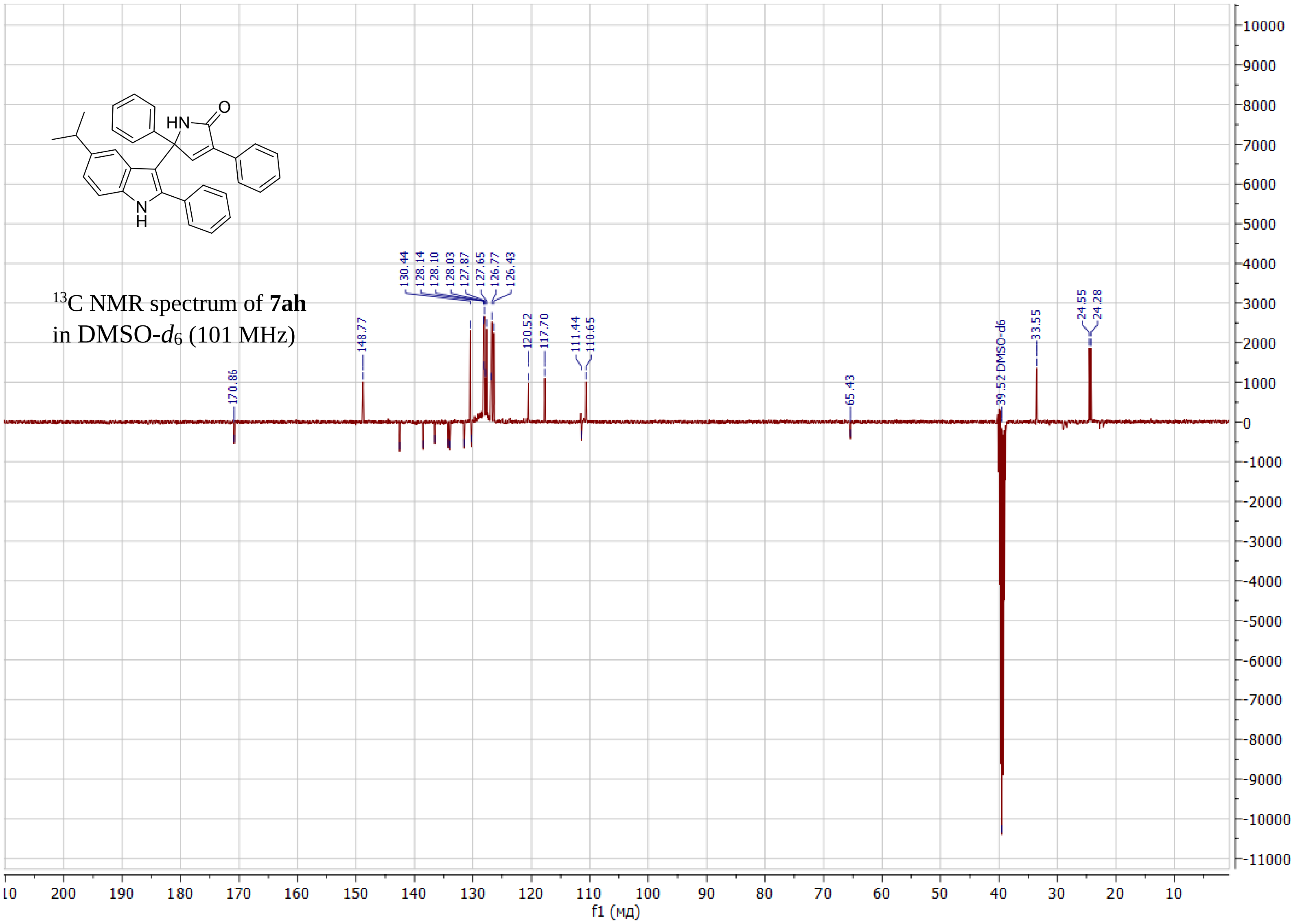

Figure S34. ^{13}C NMR spectrum of **7ah** in DMSO-d_6 (101 MHz)

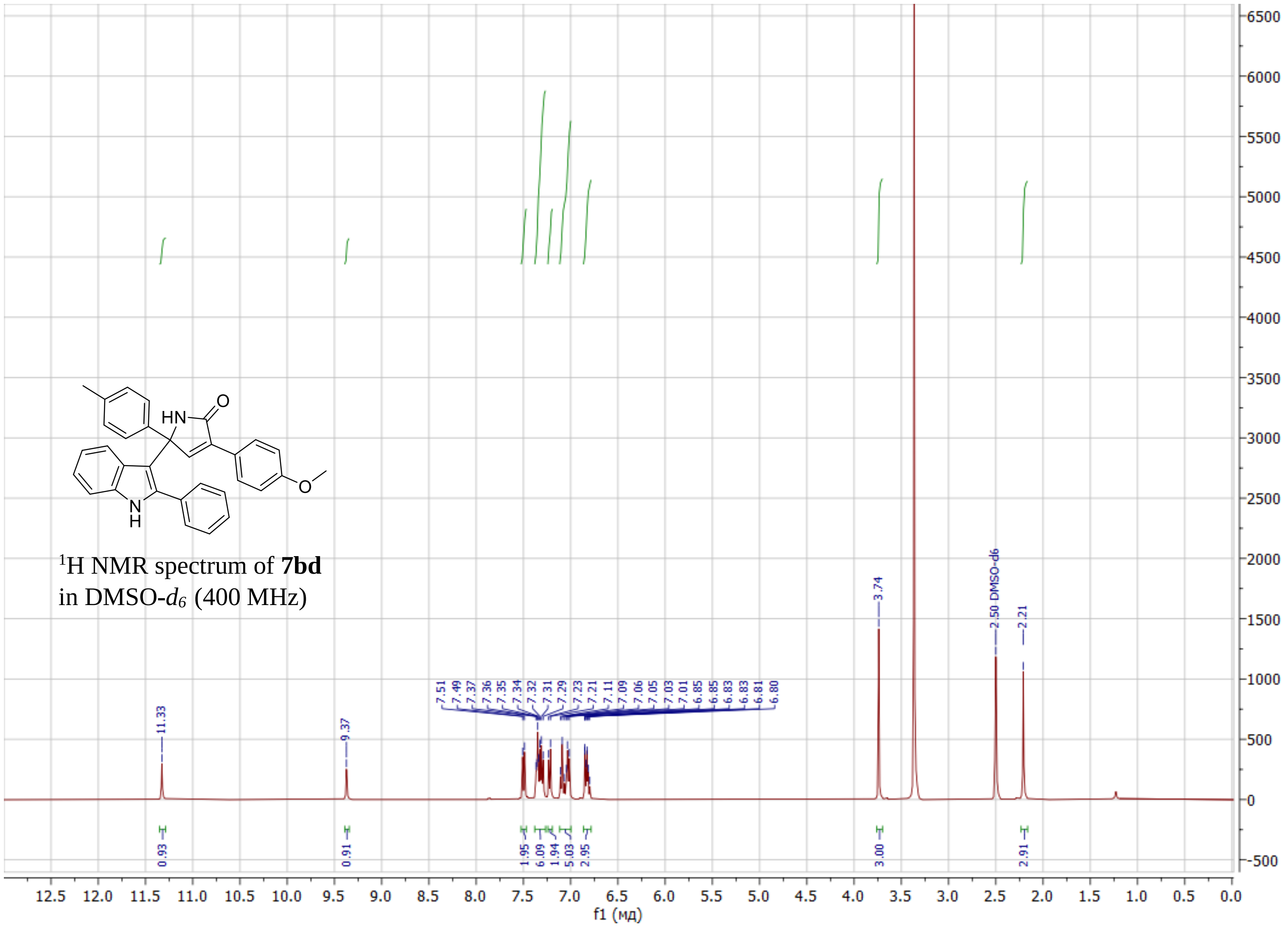

Figure S35. ^{1}H NMR spectrum of **7bd** in DMSO-d_6 (400 MHz)

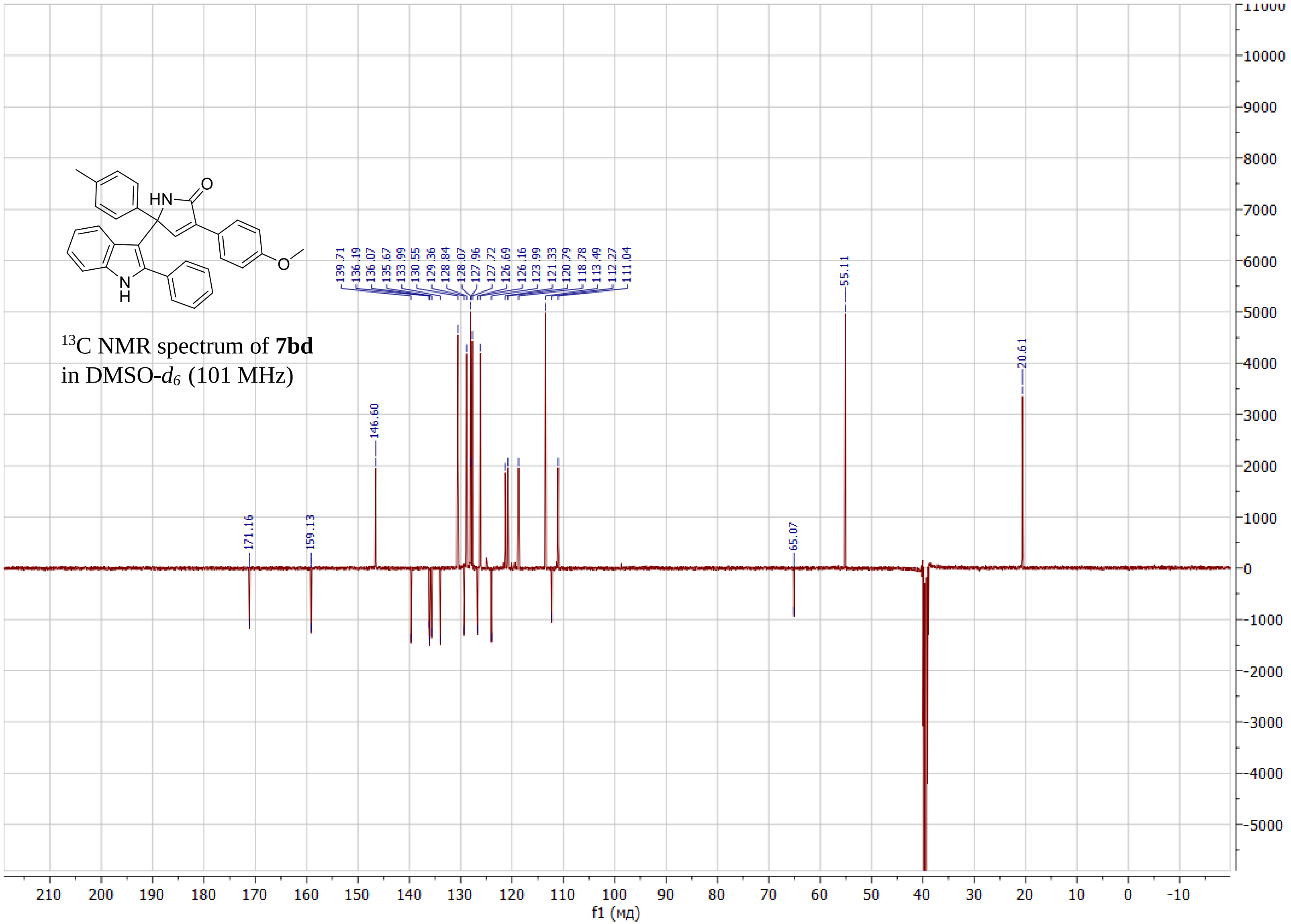

Figure S36. ^{13}C NMR spectrum of **7bd** in DMSO-d_6 (101 MHz)

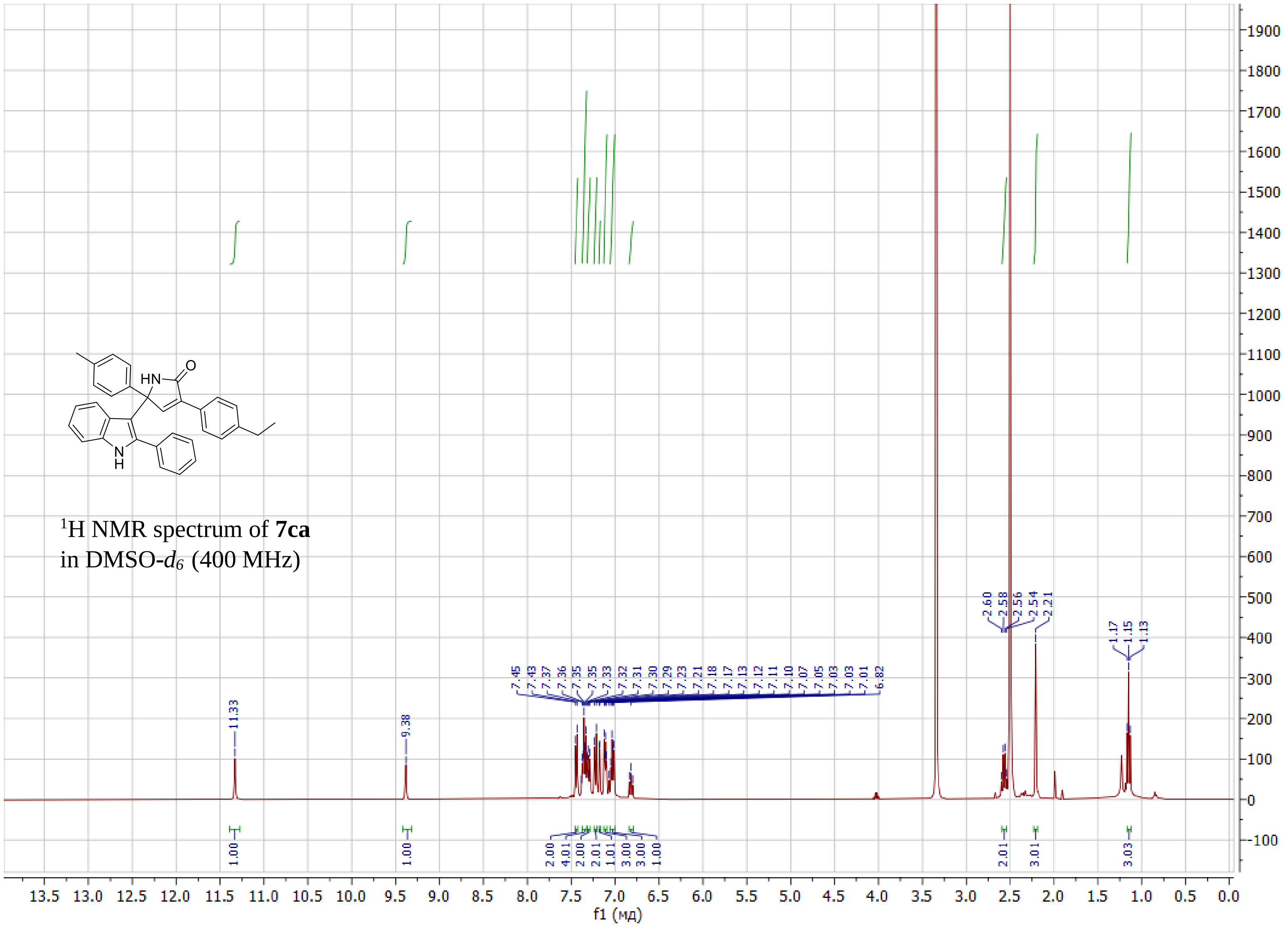

Figure S37. ^{1}H NMR spectrum of **7ca** in DMSO-d_6 (400 MHz)

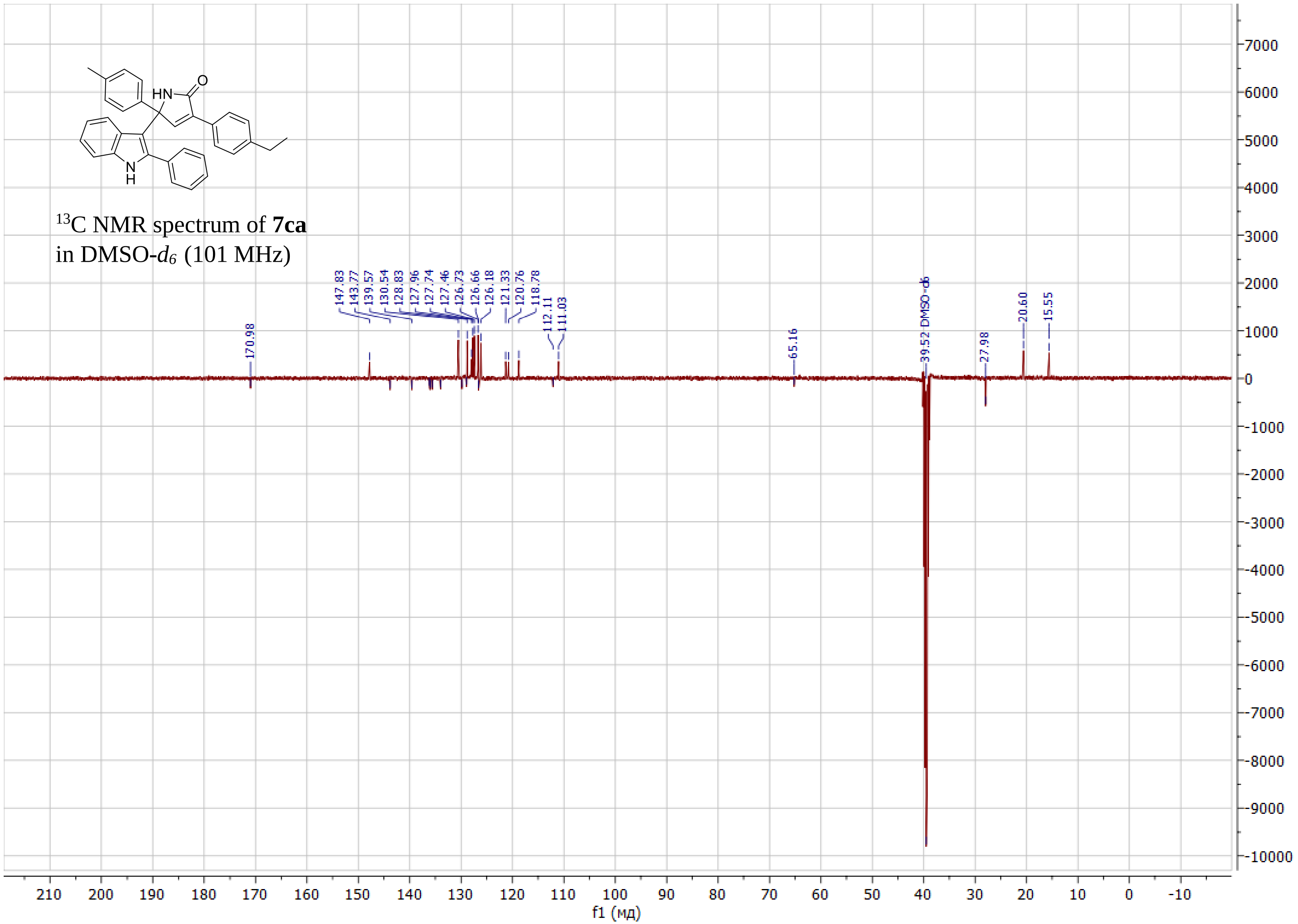

Figure S38. ^{13}C NMR spectrum of **7ca** in DMSO-d_6 (101 MHz)

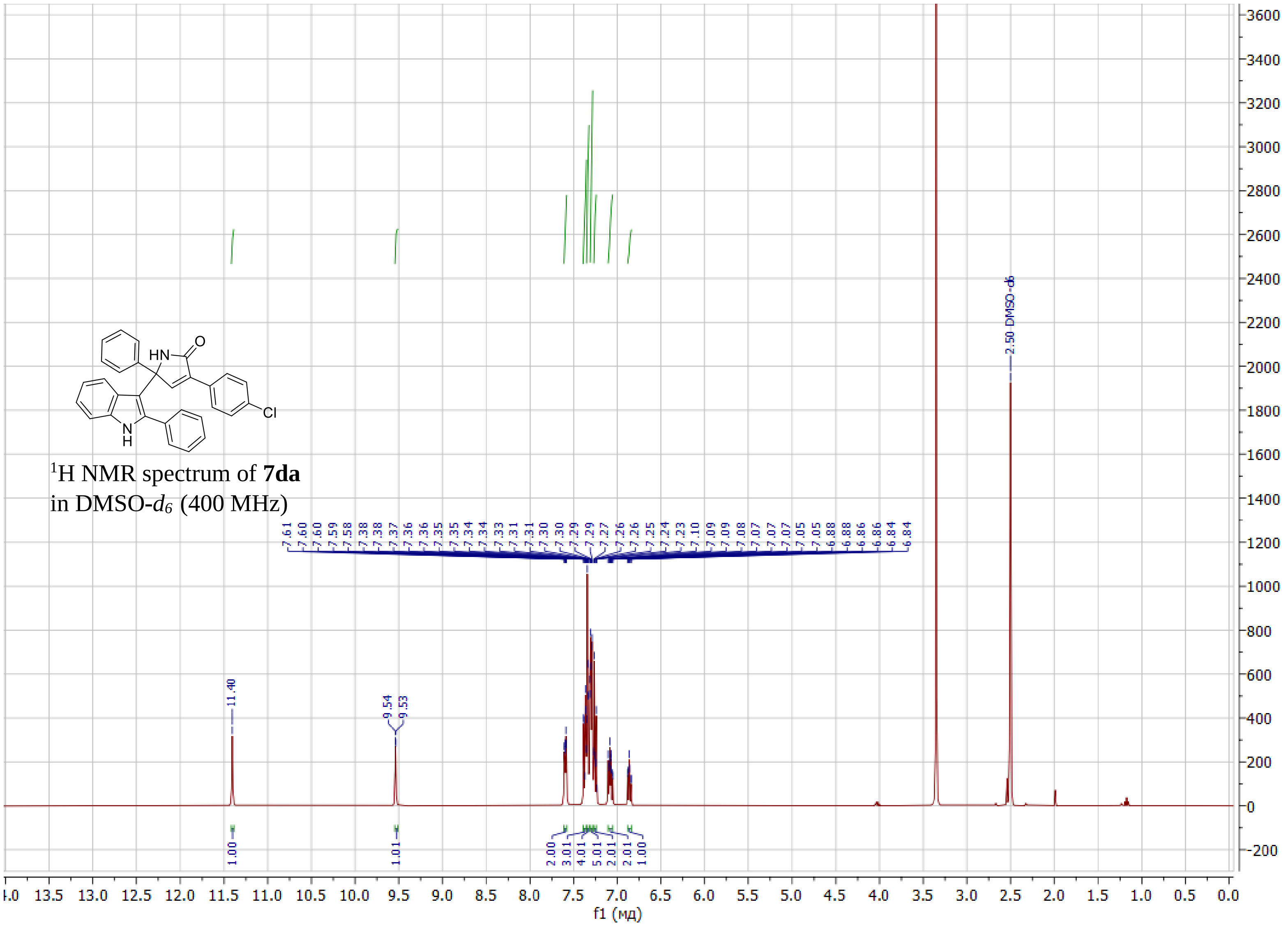

Figure S39. ^{1}H NMR spectrum of **7da** in DMSO-d_6 (400 MHz)

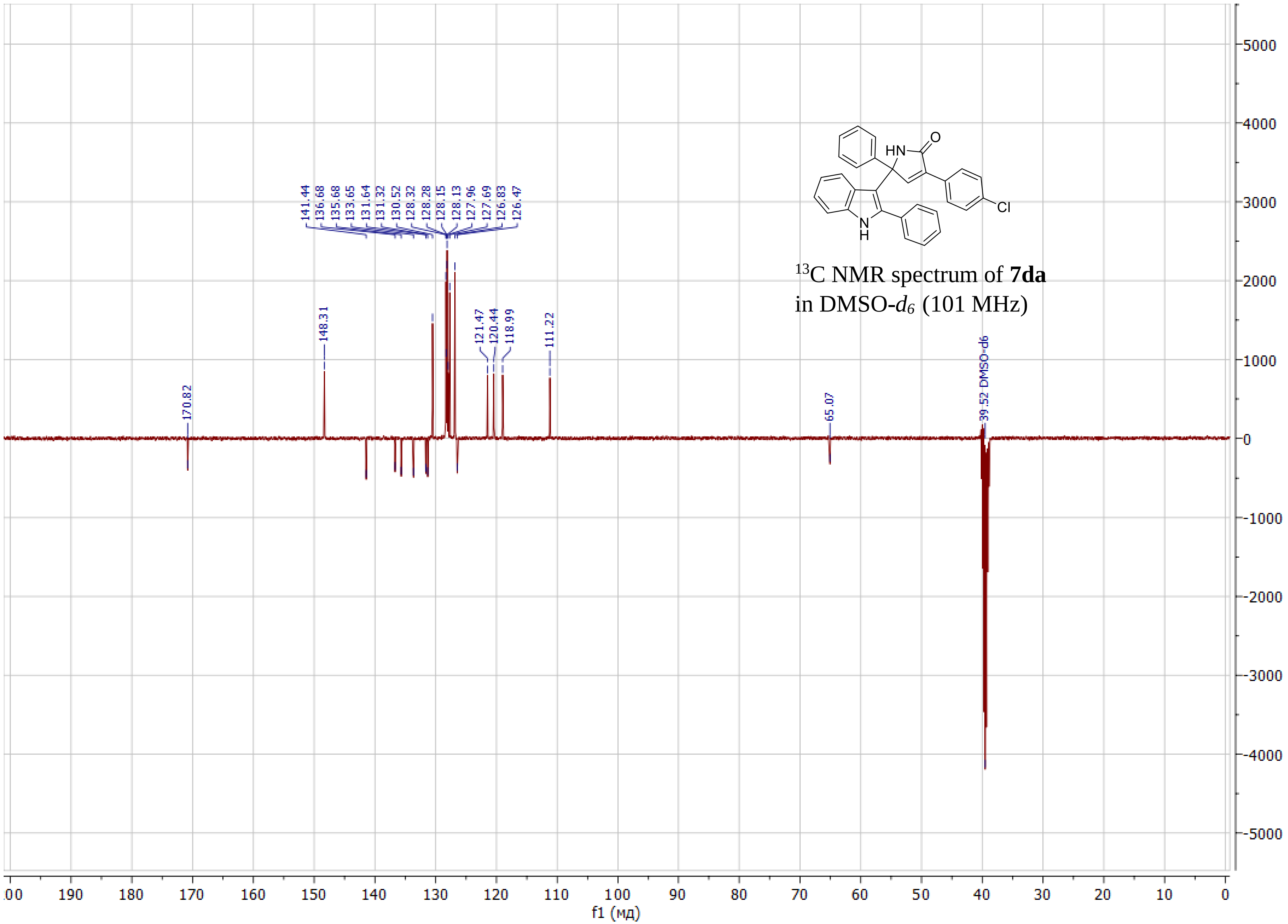

Figure S40 ^{13}C NMR spectrum of **7da** in DMSO-d_6 (101 MHz)

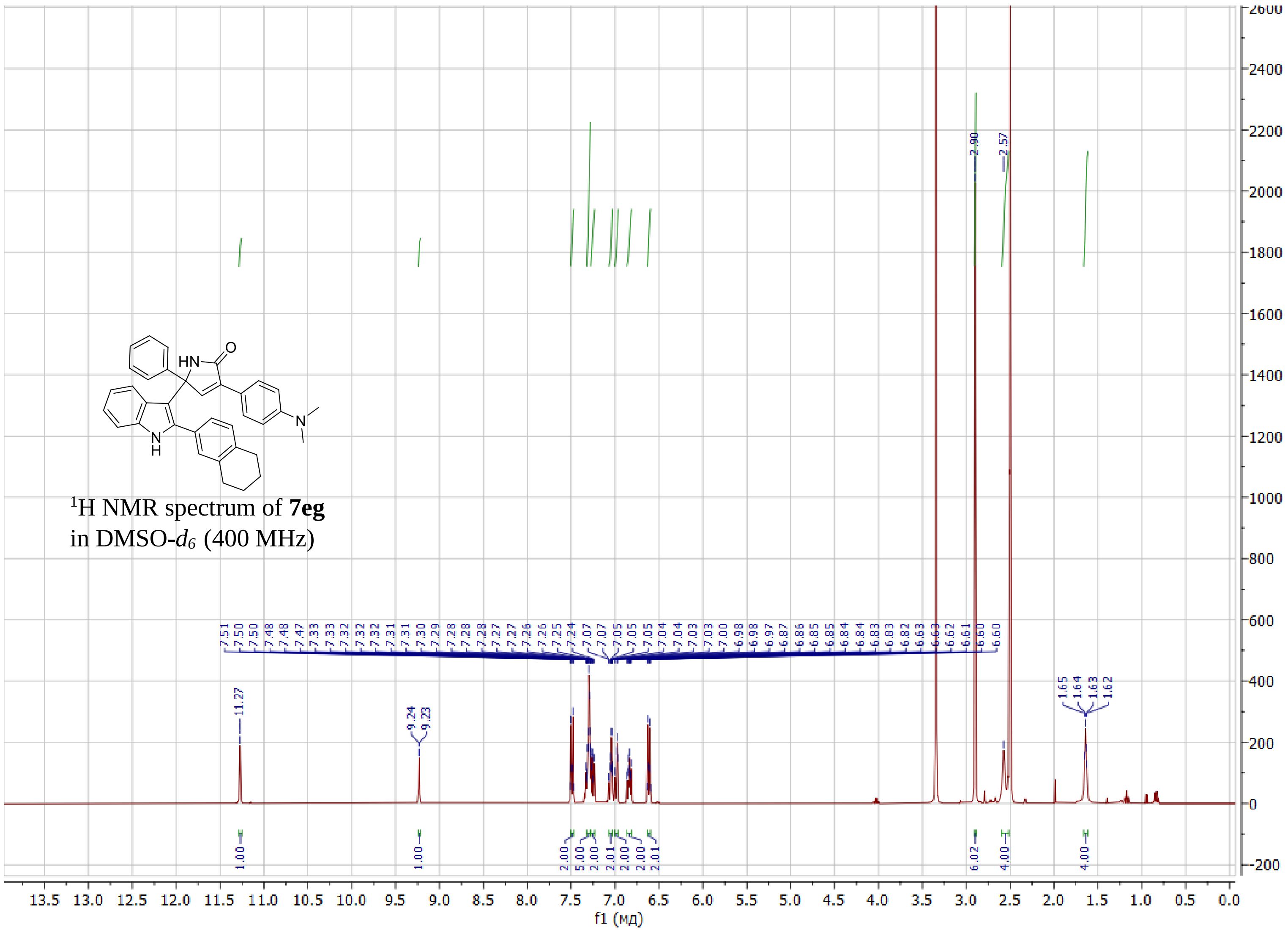

Figure S41. ^{1}H NMR spectrum of **7eg** in DMSO-d_6 (400 MHz)

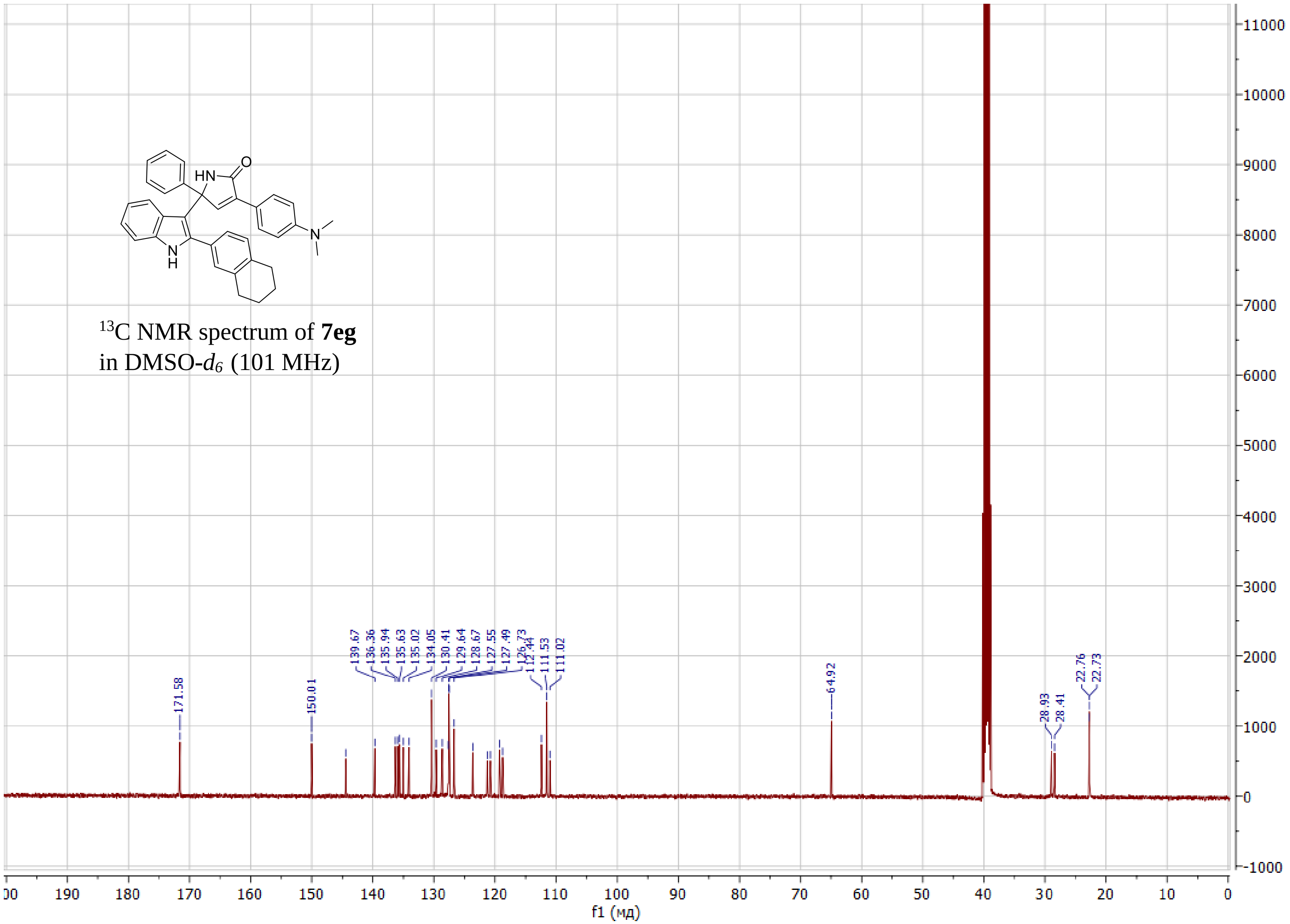

Figure S42. ^{13}C NMR spectrum of **7eg** in DMSO-d_6 (101 MHz)

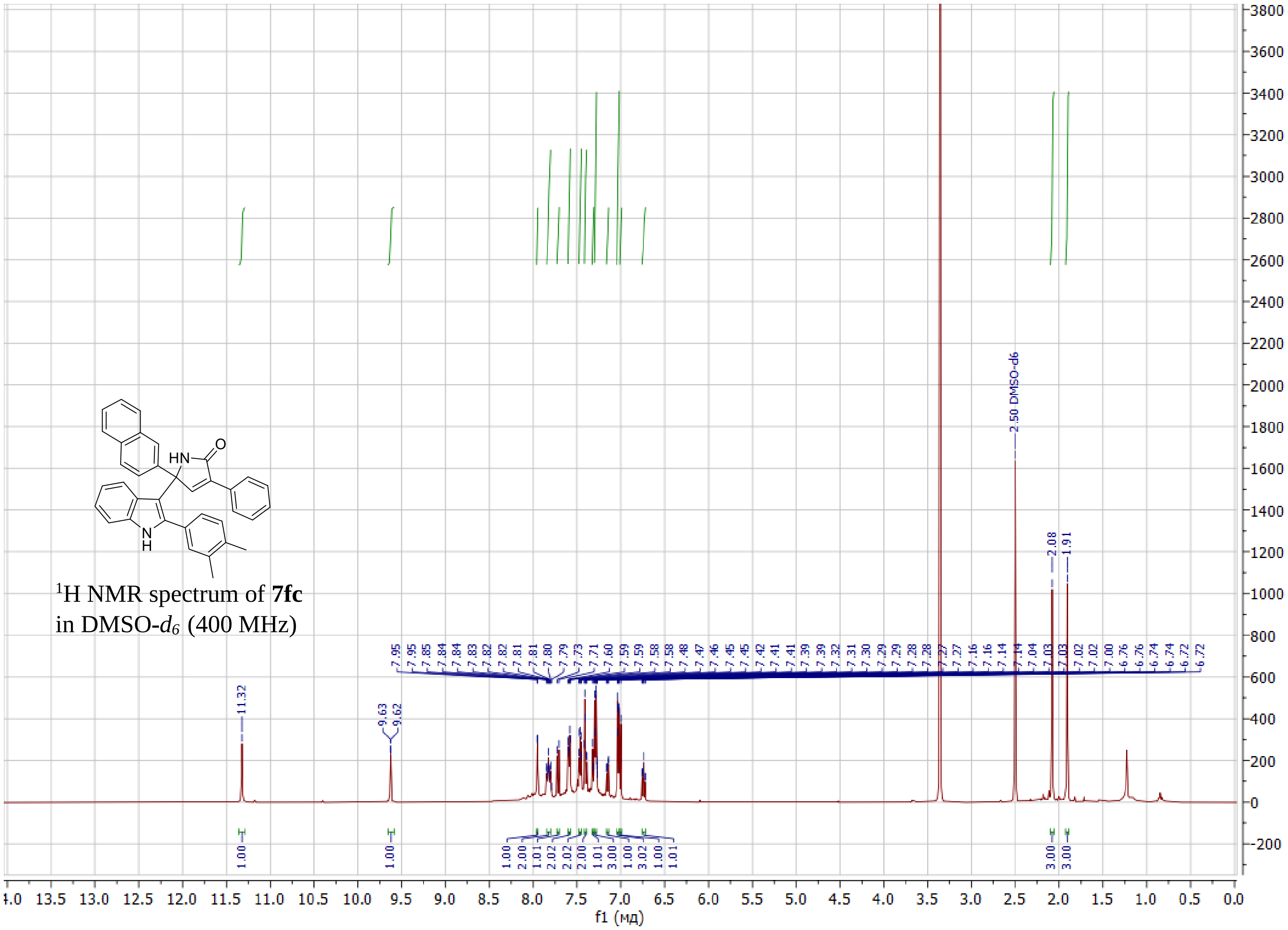

Figure S43. ^{1}H NMR spectrum of **7fc** in DMSO-d_6 (400 MHz)

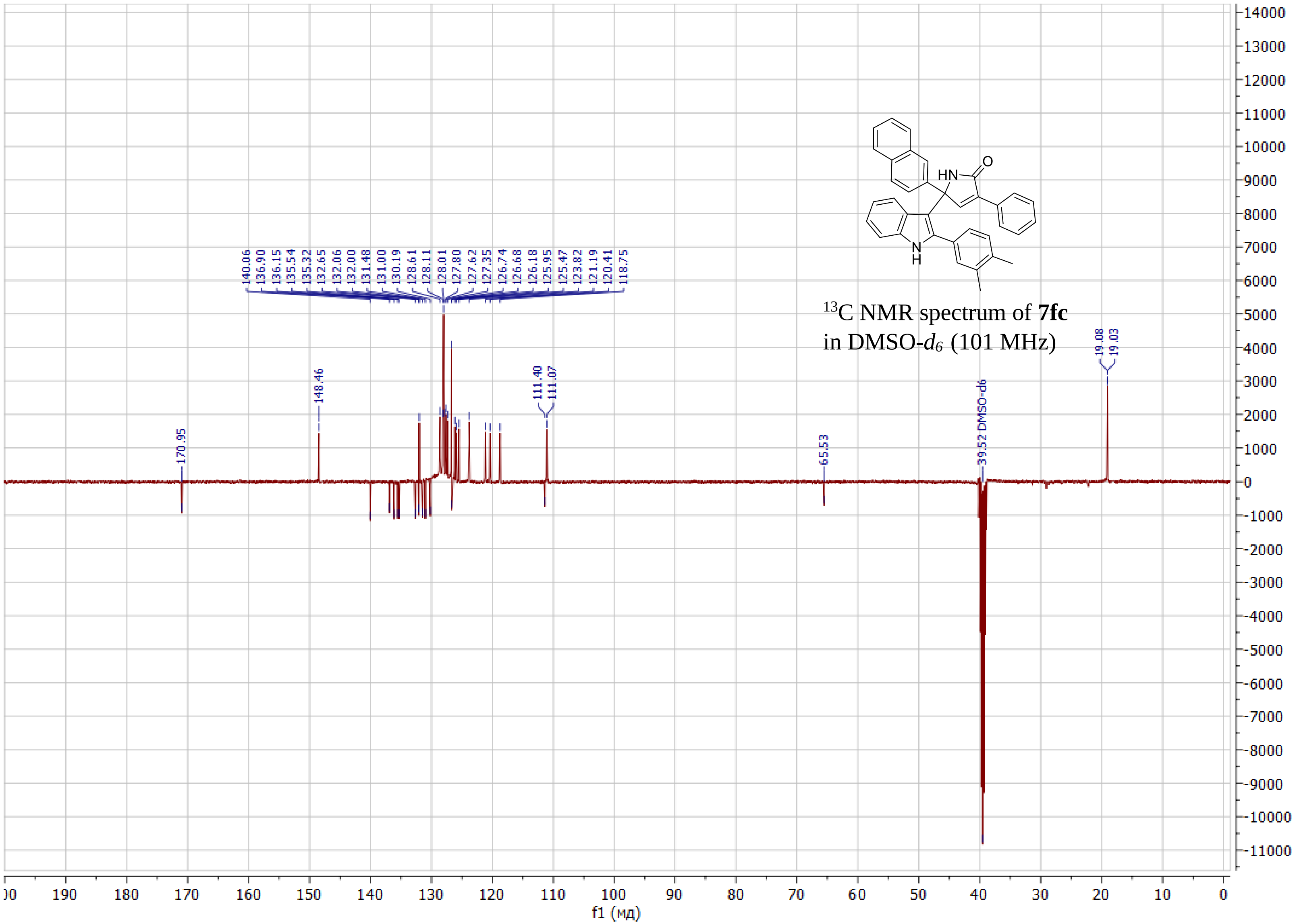

Figure S44. ^{13}C NMR spectrum of **7fc** in DMSO-d_6 (101 MHz)

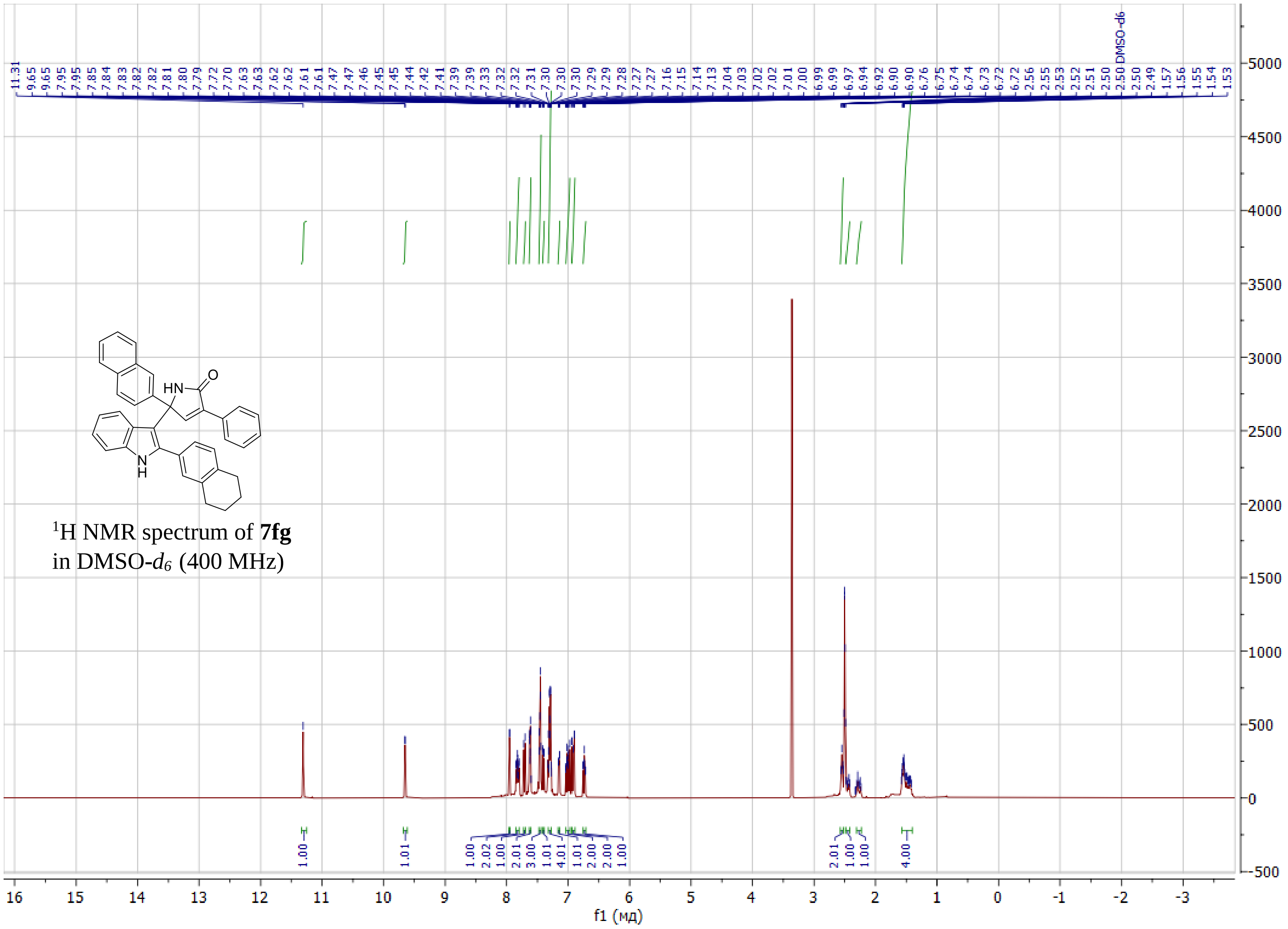

Figure S45. ^{1}H NMR spectrum of **7fg** in DMSO-d_6 (400 MHz)

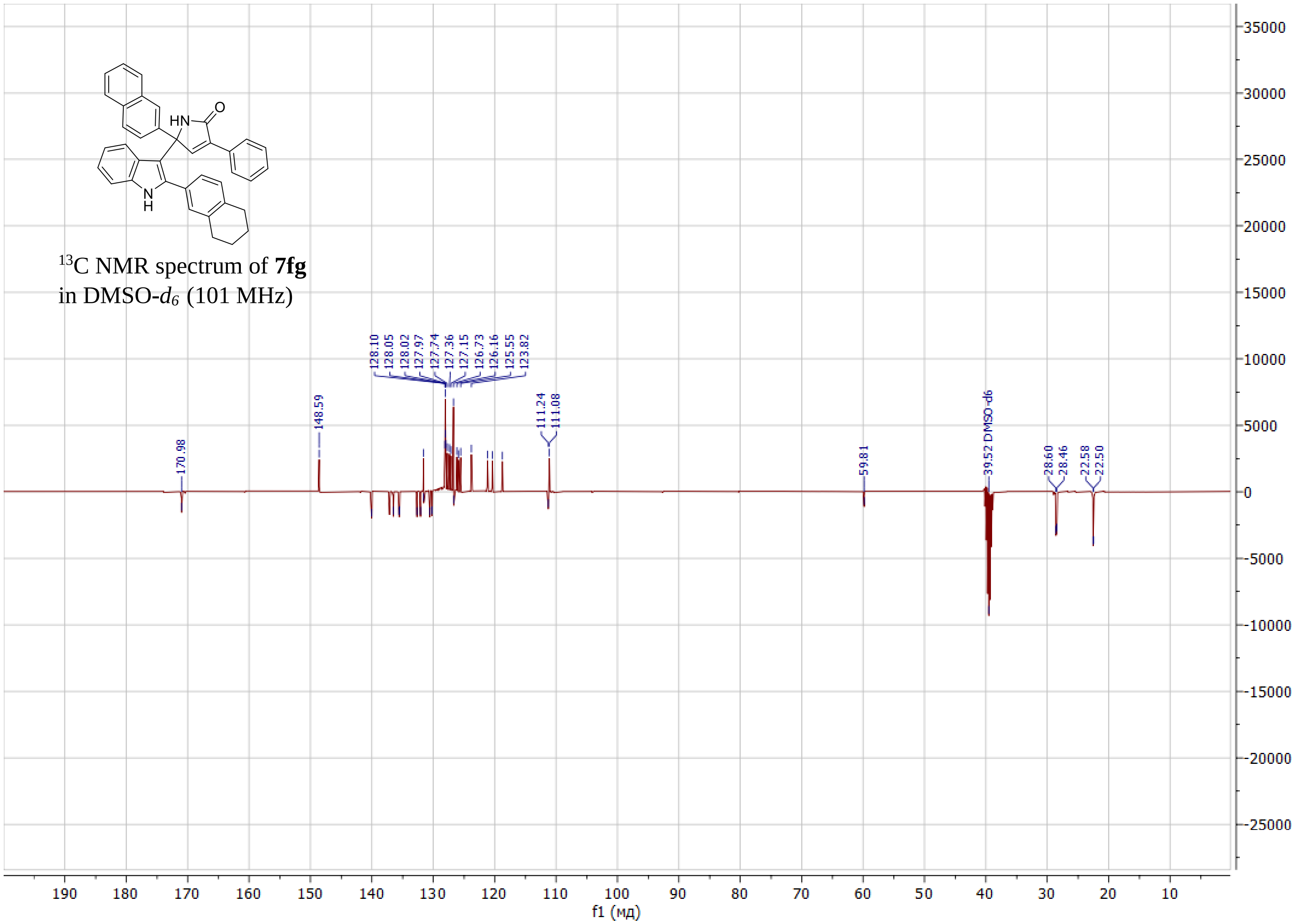

Figure S46. ^{13}C NMR spectrum of **7fg** in DMSO-d_6 (101 MHz)

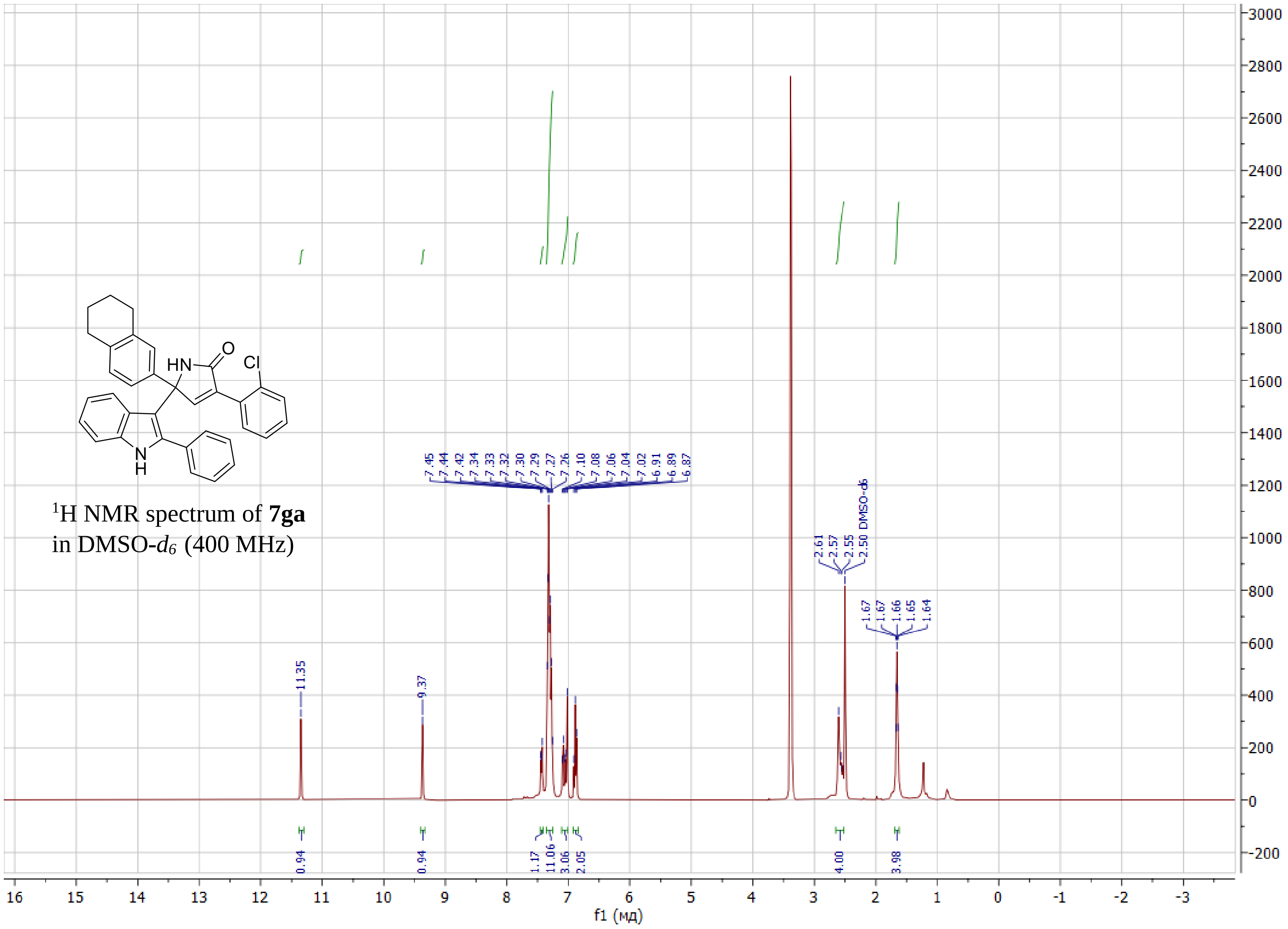

Figure S47. ^{1}H NMR spectrum of **7ga** in DMSO-d_6 (400 MHz)

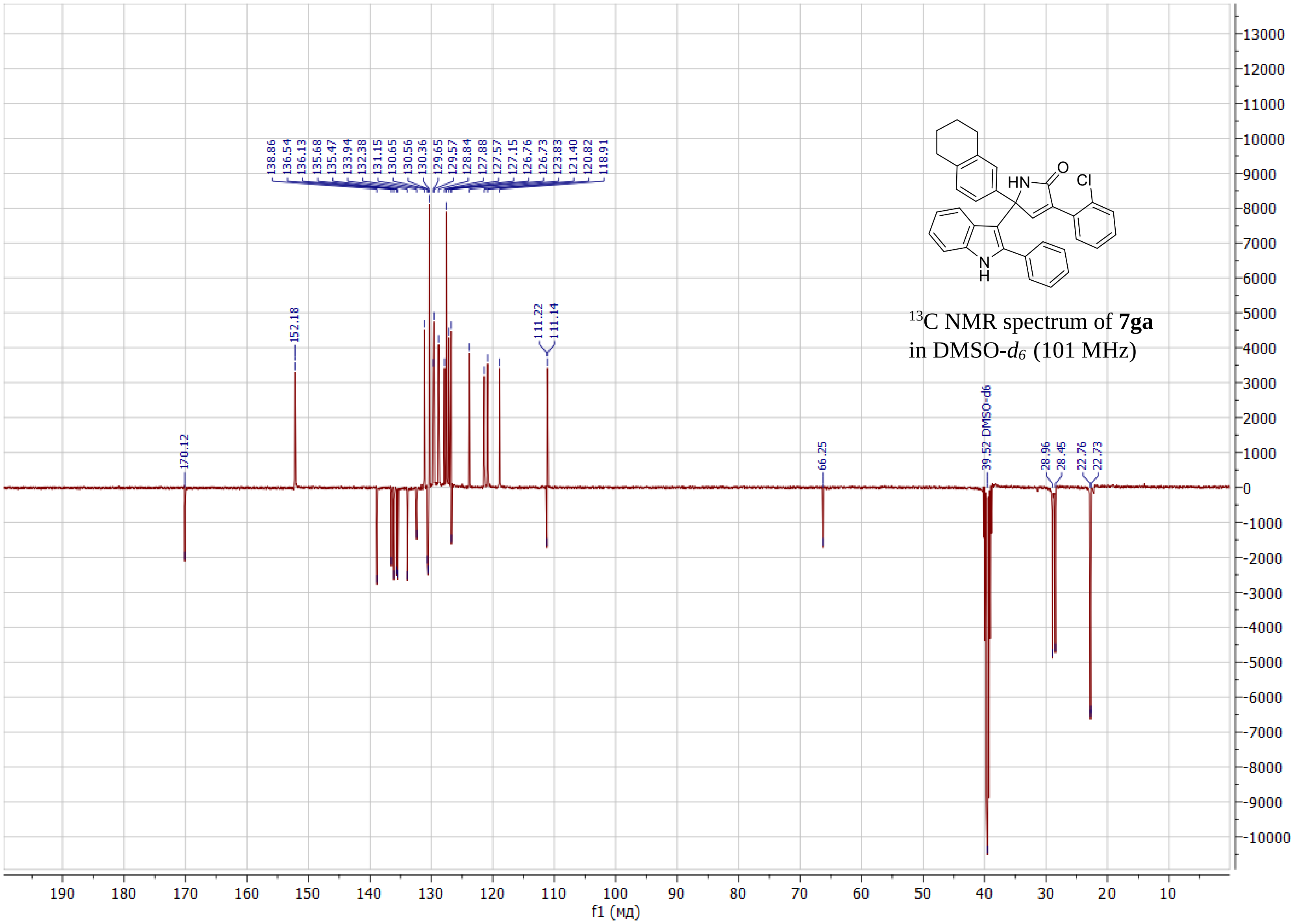

Figure S48. ^{13}C NMR spectrum of **7ga** in DMSO-d_6 (101 MHz)

^{1}H and ^{13}C NMR spectral charts for indolyl hydroxypyrrolone **10a**

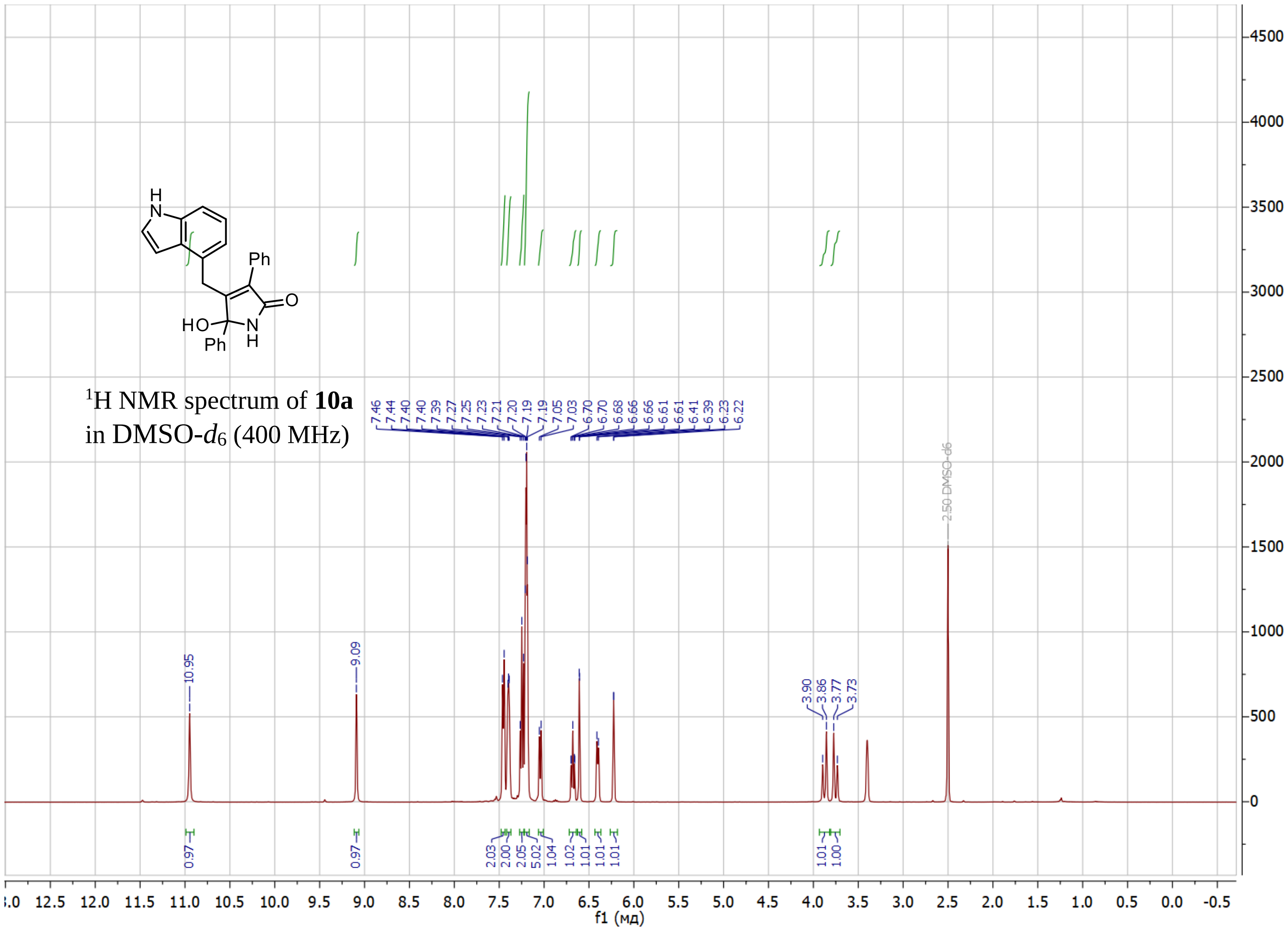

Figure S49. ^{1}H NMR spectrum of **10a** in DMSO-d_6 (400 MHz)

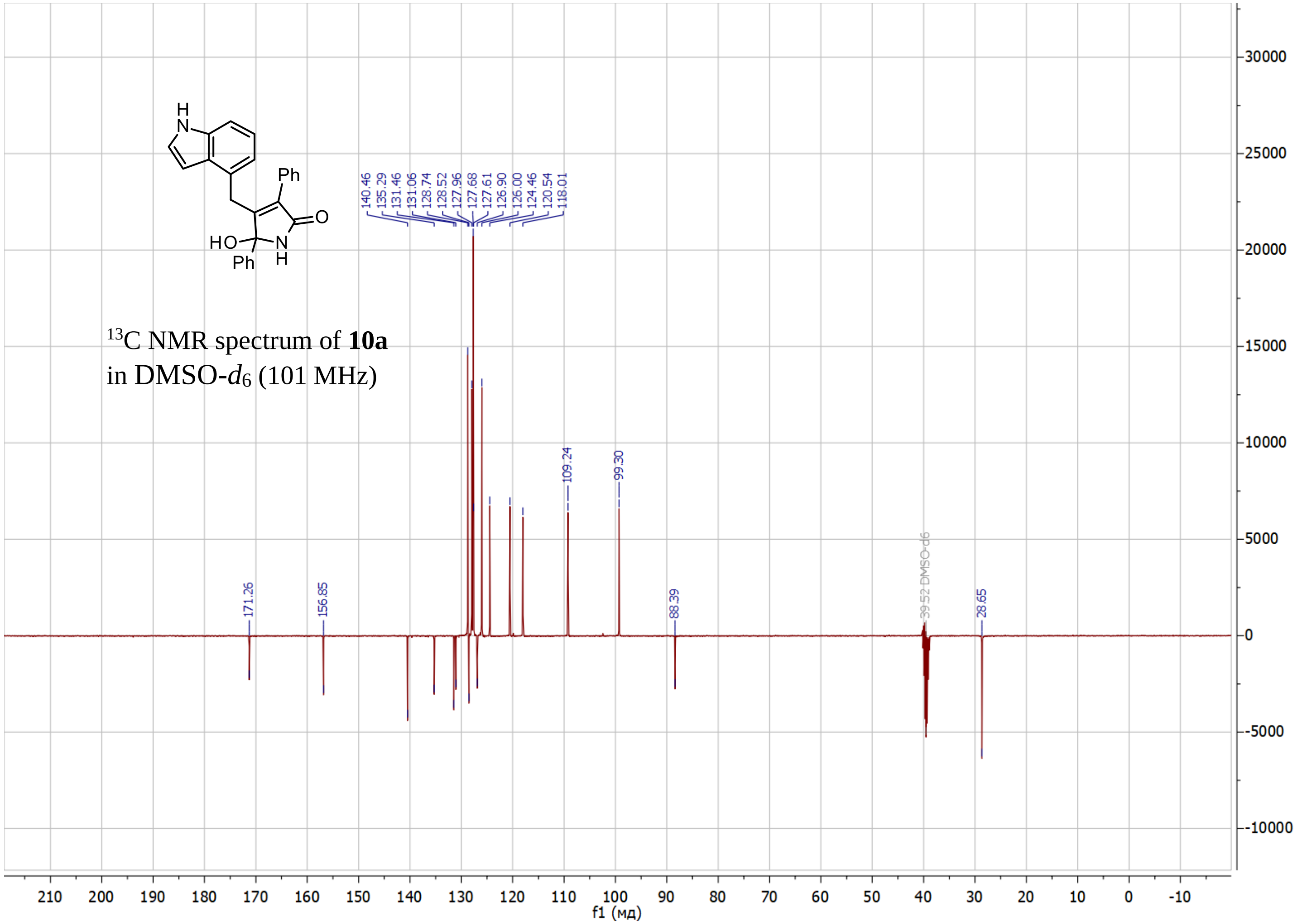

Figure S50. ^{13}C NMR spectrum of **10a** in DMSO-d_6 (101 MHz)
